# Progress in silicon-based reconfigurable and programmable all-optical signal processing chips

**DOI:** 10.1007/s12200-025-00154-6

**Published:** 2025-05-12

**Authors:** Jing Xu, Wenchan Dong, Qingzhong Huang, Yujia Zhang, Yuchen Yin, Zhenyu Zhao, Desheng Zeng, Xiaoyan Gao, Wentao Gu, Zihao Yang, Hanghang Li, Xinjie Han, Yong Geng, Kunpeng Zhai, Bei Chen, Xin Fu, Lei Lei, Xiaojun Wu, Jianji Dong, Yikai Su, Ming Li, Jianguo Liu, Ninghua Zhu, Xuhan Guo, Heng Zhou, Huashun Wen, Kun Qiu, Xinliang Zhang

**Affiliations:** 1 Wuhan National Laboratory for Optoelectronics and School of Optical and Electronic Information, Huazhong University of Science and Technology, Wuhan, 430074 China; 2 Optics Valley Laboratory, Wuhan, 430074 China; 3 State Key Laboratory of Advanced Optical Communication Systems and Networks, Department of Electronic Engineering, Shanghai Jiao Tong University, Shanghai, 200240 China; 4 Key Lab of Optical Fiber Sensing and Communication Networks, University of Electronic Science and Technology of China, Chengdu, 611731 China; 5 Institute of Intelligent Photonics, Nankai University, Tianjin, 300071 China; 6 Key Laboratory of Optoelectronic Materials and Devices, Institute of Semiconductors, Chinese Academy of Sciences, Beijing, 100083 China; 7 College of Physics and Optoelectronic Engineering, Shenzhen University, Shenzhen, 518060 China; 8 School of Electronic and Information Engineering, Beihang University, Beijing, 100191 China

**Keywords:** All-optical signal processing (AOSP), Optical nonlinearity, Low-loss silicon waveguides, Reconfigurable optical filters, Programmable optical logic array, Optical regeneration, High-density optoelectronic packaging

## Abstract

Taking the advantage of ultrafast optical linear and nonlinear effects, all-optical signal processing (AOSP) enables manipulation, regeneration, and computing of information directly in optical domain without resorting to electronics. As a promising photonic integration platform, silicon-on-insulator (SOI) has the advantage of complementary metal oxide semiconductor (CMOS) compatibility, low-loss, compact size as well as large optical nonlinearities. In this paper, we review the recent progress in the project granted to develop silicon-based reconfigurable AOSP chips, which aims to combine the merits of AOSP and silicon photonics to solve the unsustainable cost and energy challenges in future communication and big data applications. Three key challenges are identified in this project: (1) how to finely manipulate and reconfigure optical fields, (2) how to achieve ultra-low loss integrated silicon waveguides and significant enhancement of nonlinear effects, (3) how to mitigate crosstalk between optical, electrical and thermal components. By focusing on these key issues, the following major achievements are realized during the project. First, ultra-low loss silicon-based waveguides as well as ultra-high quality microresonators are developed by advancing key fabrication technologies as well as device structures. Integrated photonic filters with bandwidth and free spectral range reconfigurable in a wide range were realized to finely manipulate and select input light fields with a high degree of freedom. Second, several mechanisms and new designs that aim at nonlinear enhancement have been proposed, including optical ridge waveguides with reverse biased PIN junction, slot waveguides, multimode waveguides and parity-time symmetry coupled microresonators. Advanced AOSP operations are verified with these novel designs. Logical computations at 100 Gbit/s were demonstrated with self-developed, monolithic integrated programmable optical logic array. High-dimensional multi-value logic operations based on the four-wave mixing effect are realized. Multi-channel all-optical amplitude and phase regeneration technology is developed, and a multi-channel, multi-format, reconfigurable all-optical regeneration chip is realized. Expanding regeneration capacity via spatial dimension is also verified. Third, the crosstalk from optical as well as thermal coupling due to high-density integration are mitigated by developing novel optical designs and advanced packaging technologies, enabling high-density, small size, multi-channel and multi-functional operation with low power consumption. Finally, four programmable AOSP chips are developed, i.e., programmable photonic filter chip, programmable photonic logic operation chip, multi-dimensional all-optical regeneration chip, and multi-channel and multi-functional AOSP chip with packaging. The major achievements developed in this project pave the way toward ultra-low loss, high-speed, high-efficient, high-density information processing in future classical and non-classical communication and computing applications.

**Graphical Abstract:**

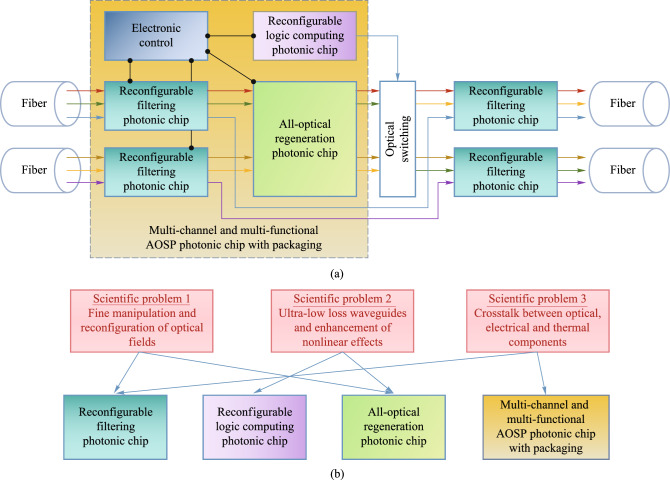

## Introduction

### Background

The advent of big data era raising significant challenges in information processing, especially in the aspect of capacity and power consumption. Situations become even worse when we consider the fact that over 90% of data information is transmitted through light while most of the information processing is carried out in the electrical domain. Two different strategies may be adopted to solve this problem. One strategy is to perform the optical-electrical-optical (O-E-O) conversion, while the other one is to process optical information directly in the optical domain, termed all-optical information processing (AOSP). AOSP may allow much more favorable scaling with the system complexity, cost as well as power consumption compared to O-E-O conversion due to several reasons. First, the difference between the speed of optical data transmission and electronic processing may go up to three orders of magnitude. In case of O-E-O conversion, information has thus to be demultiplexed and processed by a number of parallel channels. Therefore, the number of optical to electrical conversion devices (or vice versa) scales with the information that needs to be processed. In addition, transparency with data speed, modulation format, and wavelength is an important figure of merit for optical signal processing, which sets challenging requirements to O-E-O conversion as well as electronic processers. In contrast, AOSP may be able to process data as a whole without resorting to data de-aggregation, and support full transparency with respect to all three aspects when proper nonlinear process is chosen. While AOSP have been widely explored as early as 1980s with bulk nonlinear devices, a rapid growth of relevant research toward AOSP integration has been witnessed recently. Among various integration platform, silicon-based photonic integration turns out to be a promising platform for the development of advanced AOSP devices and functionalities. The functionalities of AOSP are rather diverse, which strongly related to the structure of optical networks. Future optical networks require performance of 3T (format transparency, wavelength transparency, bandwidth transparency), 3M (multi-function, multi-channel, multi-network), and 3S (self-perceiving, self-learning, self-adopting) [1]. Consequently, reconfigurability and flexibility are critical to future optical networks as well as AOSP on top of ultra-large capacity.

**Fig. 1:**
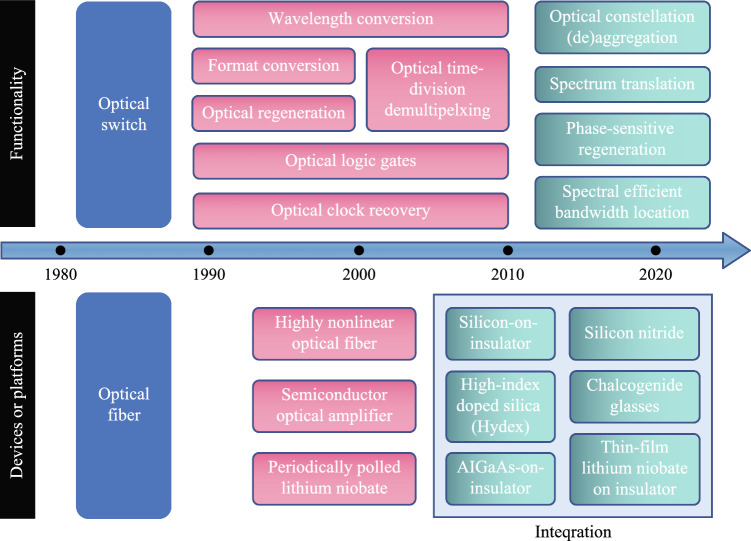
Timeline for the development of all-optical signal processing

The development of AOSP is progressing steadily over past 40 years with advances in both performances and functionalities (Fig. 1). Ultrafast optical switches have been investigated in the early days of fiber-optic communications when wavelength-division multiplexing (WDM) has not been widely explored due to lack of optical amplifiers. Operations focusing on optical control and routing are developed later, including wavelength conversion, optical time-division demultiplexing, all-optical logic operation, format conversion, and all-optical clock recovery [2]. All-optical regeneration has also been widely explored to improve the reach of optical links without going back to electronics. The fast growth of internet traffic as well as advanced digital signal processing (DSP) drives the development fiber-optic communication networks toward nonlinear Shannon limit with the full access to amplitude, phase, polarization and wavelength of an optical field [3]. Therefore, AOSP evolves toward more flexibility and advanced functionalities for transparent optical networks, such as optical constellation aggregation or de-aggregation [4, 5], conversion between different modulation formats [6, 7], phase-sensitive regeneration [8–10], and spectral efficient bandwidth location [11]. In parallel, the ultrafast characteristics of optical Kerr nonlinearities has also been explored to develop advanced optical signal processing for applications in other areas, including microwave photonics [12, 13], optical computing [14–17], ultrafast optics [18, 19], as well as quantum information processing [20–23]. Apart from functionalities, the performance of AOSP has been continuously improved by looking into different devices or platforms. A variety of bulk optical nonlinear devices have been explored first, with highly nonlinear optical fibers (HNLFs) [24], individual semiconductor optical amplifiers (SOAs) [25], and periodically polled lithium niobate (PPLN) [26, 27] among the most popular explored devices. With the rapid progress in nano-fabrication technology for photonic integration over the past 20 years, a large number of nonlinear integrated AOSP devices emerge from different material platforms. The mainstream of the third-order nonlinear platforms includes silicon-on-insulator [28], silicon nitride [29], high-index doped silica (Hydex) [30], chalcogenide glasses [31], AlGaAs-on-insulator [32–34], while thin-film lithium niobate on insulator recently stands out as a promising platform for providing the second-order nonlinearities [35, 36].

### Challenges and solutions

The goal of this project is to develop reconfigurable and programmable silicon-based AOSP chips by taking the prominent advantage of silicon platform, including complementary metal oxide semiconductor (CMOS) compatibility, low loss, compact size as well as large optical nonlinearities. To satisfy the requirement of speed, modulation format and wavelength transparency, the third-order nonlinearity from anharmonic electron response has been mainly explored, offering ultrafast response up to femtoseconds. However, the main challenge of such kind of nonlinearity is also related to the ultrafast response. In general, the third-order nonlinearity is weak, requiring up to Watt level of pump power for observable nonlinear effects to occur. Therefore, the key issue to harness the third-order nonlinearity for practical applications is to enhance light-matter interaction, which can be categorized into two trends: new materials and new structures. The development in two trends can be combined as well.

In the trends of new material and integration platforms, silicon-on-insulator (SOI) platform has been most widely explored, due to its compatibility with the CMOS technology [37–39]. Due to its high refractive index, the light confinement in silicon-based integrated devices is very strong, enabling very compact size and high nonlinearity. The most challenging issue of the SOI platform for AOSP is the carrier effects, including two-photon absorption (TPA) and free-carrier absorption (FCA), which limit the highest power that can be used in nonlinearity processes [28]. Since third-order nonlinearity exists in most kinds of materials, various material platforms with low transmission loss in telecom spectrum window have been investigated. According to Miller’s rule [40], the nonlinear refractive index of materials increases exponentially with the linear refractive index of the materials. Therefore, materials with high refractive index are highly desired for AOSP. One successful example is the ternary compounds semiconductors such as AlGaAs, which has turns out to be a very promising material for constructing integrated AOSP devices [32–34]. Nevertheless, it should be remembered that materials with higher refractive index usually have smaller bandgaps and stronger nonlinearities, making them more vulnerable to multi-photon absorption. In addition, strong light-confinement due to large refractive index also leads to severe scattering losses, hindering the development of low-loss integrated optical waveguides. High losses, no matter with linear or nonlinear origin, are detrimental to the efficiency of optical nonlinearity. As a good balance, material platforms such as silicon nitride, high-index doped silica (Hydex), and chalcogenide glasses have been widely explored, with their linear refractive indexes of about 2–3. Ultralow loss optical waveguides can thus to be made (down to a few decibels per meter) while the optical nonlinearities are relatively strong. Thin-film lithium niobate on insulator (LNOI) has also attracted a lot of interests in recent years for AOPS since the cascaded second-order nonlinearity has similar effects as the third-order nonlinearity [41]. Periodically polled structures have also been successfully implemented on-chip to avoid phase mismatch between the fundamental and harmonic frequencies.

On top of material revolution, novel structures that enable enhanced light-matter interaction are developed. Intuitively, light-matter interaction can be greatly boosted by enhancing interaction length or time. Long integrated optical waveguides may enhance nonlinear efficiency given that the loss of the waveguides can be made sufficiently low. Taking the wavelength conversion for example, the conversion efficiency of four-wave mixing (FWM), defined as the power of the newly generated idler wave divided by the input signal wave, may go beyond − 10 dB when the optical waveguides are made centimeters long based on SOI platform [42]. Positive conversion efficiency, which means that for the signal waves net gain can be achieved, may be acquired based on silicon nitride platform with spiral waveguides of meter-level length [29, 43]. However, ultralow loss long waveguides are in general challenging to fabricate and occupy large footprint. On the other hand, resonators have much smaller footprint and can be used to enhance the light-matter interaction time. Recently developed high-quality (*Q*) factor microresonators have triggered great nonlinear applications such as optical frequency combs [34, 44]. Nevertheless, resonant devices are often bandwidth limited, which means they are not fully transparent to operation speed [45]. In addition, light-matter interaction can also be made by slowing down light speed using the so called slow-light photonic devices [46], or squeezing the mode volume using slot-waveguides [47] or surface plasmonic structures [48, 49]. However, these schemes facing trade-offs among fabrication complexity, low-loss, as well as efficiency. There are also interesting combinations of both material and structure innovations, e.g., slot waveguides or surface plasmonic structures combined with high-nonlinear polymer materials [50, 51]. In addition to develop nonlinear devices that perform core operation of AOSP, advanced filtering functions are highly desirable to AOSP chips to perform reconfigurable and programmable tasks. To increase the capacity of AOSP, a large number of nonlinear devices are densely packed, raising severe challenges in the crosstalk between optical, electrical and thermal components.

Therefore, three key scientific problems have been identified in this project, i.e., to finely manipulate and reconfigure optical fields, to fabricate ultra-low loss integrated silicon waveguides and significant enhance nonlinear effects, to greatly relief the crosstalk among optical, electrical and thermal components. Driven by solving these problems, the following major advances are achieved during the project. Firstly, we developed ultra- low-loss silicon-based waveguides, microresonators with ultra-high *Q*, as well as integrated photonic filters with bandwidth and free spectral range reconfigurable in a wide range, by developing key fabrication technologies as well as novel device structures. Secondly, several mechanisms and new designs that aim at nonlinear enhancement have been proposed and verified in optical logic and regeneration operations, including optical ridge waveguides with reverse biased PIN junction to sweep the generated carriers away, slot waveguides and multimode waveguides, coupled microresonators with parity-time symmetry. Thirdly, monolithic integration of programmable optical logic array is realized, and the high-dimensional multi-value logic operation devices based on the four-wave mixing effect are realized. Fourthly, multi-channel all-optical amplitude and phase regeneration technology is developed, and the multi-channel, multi- format, reconfigurable all-optical regeneration chip is realized. All-optical regeneration is also combined with mode-division multiplexing technique to increase the regeneration capacity of a single waveguide. Fifthly, progresses are made in integrated AOSP chips and packaging, featuring high-density, small size, multi-channel and multi-functional operation with low power consumption by optimizing the crosstalk from optical as well as thermal coupling due to high-density integration.

### Structures

**Fig. 2:**
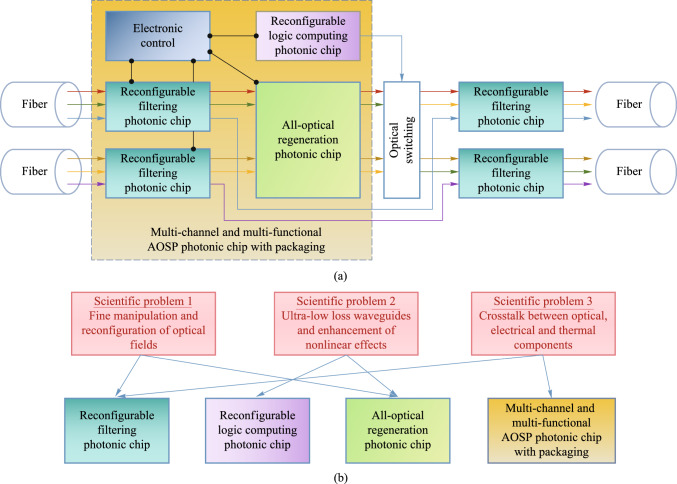
**a** Role of each photonic chip developed in this project in the scenario of flexible optical networks. **b** Relationship between chips and key scientific problems

This project is divided into four sub-projects: reconfigurable filtering photonic chip, reconfigurable all-optical logic operation photonic chip, multi-dimensional all-optical regeneration photonic chip, multi-channel and multi-functional AOSP photonic chip with packaging. The relation among four photonic chips in the scenario of flexible optical networks is shown in Fig. 2a. The relationship between each chip and scientific problem is shown in Fig. 2b. In the following, Sects. 2, 3, 4, 5 are devoted to the introduction of each photonic chip developed in this project. A final conclusion is drawn in Sect. 6.

## Reconfigurable filtering photonic chip

*Yujia Zhang, Yuchen Yin, Desheng Zeng, Qingzhong Huang, Xin Fu, Xuhan Guo, Yikai Su*.

### Current status

Optical filters are basic building blocks for integrated photonics, which can be developed on SOI platforms very compactly. Among various structures, microring resonators (MRRs) and Mach–Zehnder interferometers (MZIs) have been most widely explored for constructing optical filters on silicon photonic chips. The performances of optical filters are related to a large number of parameters, among which the *Q*-factor, bandwidth and free spectral range (FSR) are of great importance. The* Q*-factor is a parameter that represents the number of field oscillations before the circulating energy depleted to $1/e$ of the initial energy. Intuitively, *Q*-factor is highly related to the transmission loss of the optical waveguide that used to compose the optical resonator, where the less loss creates higher *Q*-factor. Therefore, the *Q*-factor is an indicator for the quality of the resonator and highly relevant to the linear and nonlinear performances. In addition, elastic optical networks (EON) allocate resources and services in a dynamic and intelligent manner. The reconfigurability of an optical filter, especially the tunability of the bandwidth and the FSR, is highly required by the need for dynamic channel allocation to increase the spectrum usage efficiency and the transmission capacity.

The *Q*-factor of a silicon MRR is limited as a result of scattering loss caused by roughness of the waveguide sidewall due to fabrication imperfections as well as bending loss [52]. The *Q*-factor can be improved via geometric innovation or improvement in the fabrication processes. Geometric design includes the usage of multimode waveguides and Euler bends. The fundamental modes in multimode waveguides in MRRs or racetrack resonators are often used to reduce mode overlap with the vertical sidewalls [53–55]. Guillén-Torres et al. obtained a *Q*-factor of 1.7 × 10^6^ by using low-loss 3-μm-wide multimode straight rib waveguides and single mode strip waveguides in the bend regions [56]. In addition, a silicon racetrack resonator, composed of multimode long straight waveguides and single mode arc waveguides, has achieved a high-*Q* value of 1.14 × 10^6^ and a wider FSR of 0.325 nm [55]. A silicon racetrack resonator based on the Euler multimode racetrack waveguide has demonstrated a *Q* value of approximately 1.3 × 10^6^ and an FSR of 0.9 nm. The use of Euler multimode waveguides can eliminate the need for tapered waveguides between single and multimode waveguides while suppressing the bending loss of circular waveguides [57]. The waveguide sidewalls can be smoothed with effective processing techniques, such as hydrogen annealing, laser burnishing, chemical–mechanical polishing, reflowing photoresist (RP), and oxidation smoothing [58, 59], to further minimize the scattering loss. Takahashi et al. obtained smooth waveguides with root mean square (RMS) roughness of 5–10, 3–5, 2–3, and 1–2 nm by oxidizing silicon waveguides at 1000 °C for 0, 18, 75, and 165 min, respectively [60]. An AlGaAs-on-insulator nanowaveguide has been used to achieve an ultrahigh-*Q* MRR with a *Q* value of 3.52 × 10^6^ using RP and heterogeneous wafer bonding methods [61]. Hung et al. utilized a KrF excimer laser to illuminate the surface of silicon waveguides, making the silicon material molten [62]. As a result, the waveguides became smoother under surface tension and they succeeded in reducing the RMS of the waveguide roughness from 14 to 0.24 nm. However, laser irradiation is not easy to perform and requires expensive equipment. RP and oxidation smoothing techniques are simple and effective methods for achieving an ultrahigh-*Q* MRR on SOI platform. Note that apart from optical filters [63–65], the applications of MRRs also include optical frequency combs [34, 66, 67], optical buffers [68, 69], optical switches [70], and biosensors [71]. As already mentioned, MZI structure is also used to construct optical filters, as reported in Ref. [72].

EON has been regarded as a promising solution for the explosive growth of traffic in optical communication networks [73, 74]. EON architecture can vastly enhance the network efficiency by tearing down the traditional rigid wavelength granularity and adaptively assigning spectrum resources to satisfy actual traffic demands. The flexible and even programmable optical filters are indispensable building blocks in the development of highly functional circuits for such agile optical networks. The tunability of central wavelength, bandwidth and FSR are of core importance to the reconfigurable optical filter in EON. In recent years, the demand for flexible optical filters with multiple functions, such as simultaneously tunable wavelength and bandwidth has been increasing. To meet this need, a variety of silicon filtering configurations with multiple basic components have been developed, including the cascaded MRRs [75–77], loop mirrors or MRRs assisted MZIs [78–80] and MZIs [81], cascaded Bragg-grating-assisted contra-directional (DC) couplers [82], and the loop of multimode Bragg gratings [83]. In Ref. [75], two fifth-order MRRs are cascaded, showing the tuning of 3 dB bandwidth from 12 to 125 GHz and a high extinction ratio of 100 dB. In Ref. [78], two serially coupled MRRs are equipped with three thermally tuned MZIs, which can realize wide-bandwidth tunability from 9 to 103 GHz and full FSR central wavelength tuning with an efficiency of 0.297 mW/GHz. Moreover, an on-chip programmable optical filter based on four-tap MZIs demonstrates a flexible tunability in central wavelength, bandwidth, and passband shape [81]. By cascading a pair of contra-DCs, an integrated band-pass filter with continuously bandwidth tuned over 670 GHz (117–788 GHz) and a 4 nm central wavelength tuning range is obtained in Ref. [82]. Besides, the scheme based on a loop of multimode waveguide Bragg grating is proposed with a wide 3 dB bandwidth tunability of about 11.64 nm and flat-top responses [83]. Despite significant progress in integrated photonic filters, the flexible filter with a reconfigurable FSR has not yet been demonstrated, with only a few studies based on conventional optical fibers [84, 85].

### Challenges and solutions

The design of optical filters is still facing several challenges, including achieving ultra-high *Q*-factors, the large reconfigurability of bandwidth and FSR.

As mentioned before, the *Q*-factor of optical filters is limited by scattering loss due to fabrication imperfections as well as bending loss [52]. The scattering loss is caused by the roughness on the sidewall of the waveguide, which can be reduced by several processing techniques including chemical–mechanical polishing and oxidation smoothing [58, 59]. However, it is difficult to maintain the stable performance using such processing techniques in large-scale photonic integrated chips. Hence, the geometric designs of the waveguide are commonly used in reducing the scattering loss and the bending loss. Fundamental mode in a multimode waveguide has a lower scattering loss compared to that in a single mode waveguide, but has a higher bending loss when the multimode waveguide is used in MRRs due to the mode mismatch effect. Several geometric designs are used in the bending region of the multimode waveguide MRRs to minimize the scattering loss and bending loss. We propose a micro-racetrack resonator with the Euler bend multimode waveguides which achieves a high *Q*. The Euler bend waveguides can suppress the mode mismatch effect between the bending and the straight waveguide, and two concentric circular arc waveguides satisfying the mode matching condition in the bus waveguide and resonator coupling region are used to avoid the coupling between higher-order modes and fundamental mode.

As for the reconfigurabilities of the bandwidth and the FSR, they are limited by device designs and fabrication imperfections. Filters with widely tunable bandwidth and on-chip FSR reconfigurability are of great importance but still challenging to obtain. Optical filters often require high extinction ratios, which can be realized by biasing the coupling region of a single MRR at the critical coupling condition. Bandwidth tunability can be realized by changing the coupling condition, which changes the extinction ratio as well. In general, the bandwidth tunability of MRRs facing the trade-off with the intrinsic *Q* of the resonator. We propose MZIs-assisted MRRs with shallow-etched multimode waveguides and an optical switch to realize a large tunable bandwidth. The shallow-etched multimode waveguides reduce the scattering loss and achieve a high *Q* in the filter. The optical switch is used to realize a large bandwidth tuning range by switching between two filters featuring distinct intrinsic *Q*.

The lack of on chip FSR reconfigurability is resulting from difficulties in the complex device design and tuning range. The FSR of an MRR is in general fixed once the device is fabricated and tunability is often achieved by cascading more devices. Achieving more than ten times FSR reconfigurability is difficult in the on chip optical filters. We propose an FSR-reconfigurable filter based on five cascaded MZIs and five optical switches. The optical switches change the overall length difference between the two MZIs arms, realizing a large range and discrete reconfigurable FSR.

### Progress

#### Ultra-low-loss silicon-based waveguides and MRRs with ultra-high *Q* factor

Ultra-high *Q* MRRs have broad application prospects in narrow-band optical filtering, optical signal processing, and nonlinear optical applications. Ultra-low-loss silicon-based waveguides are fundamental elements to achieve ultra-high *Q* MRRs. There are many factors that restrict the MRR to achieve an ultra-high-*Q*, the most critical of which is the waveguide loss. The loss mainly comes from the following aspects: (1) the scattering loss caused by the rough side wall of the waveguide; (2) the absorption loss caused by the dangling bonds and defects on the waveguide surface; (3) the bending radiation loss caused by the inconsistency of the angular velocity of the inner and outer light waves in the bending waveguide; (4) the coupling radiation loss caused by the change of the spacing of the waveguide coupling region.

Therefore, we reduce the waveguide loss from both design and process. First, multimode ridge waveguides are used as the resonator waveguides, leading to a smaller overlap between the mode and the waveguide sidewall and thus reducing the scattering loss. Second, the reflowing photoresist on a hotplate at a typical temperature is carried out after the development. The pattern edge becomes smoother under the action of liquid surface tension. Third, the optical waveguide can be thermally oxidized to eliminate the surface dangling bonds. A thin oxide layer will grow on the surface of the waveguide to compensate the surface dangling bonds and reduce the absorption. Moreover, since the tiny protruding part of the surface is oxidized quickly and the concave part is oxidized slowly, the surface roughness of the waveguide can also be reduced. To reduce the radiation loss caused by the inconsistency of the angular velocity of light field in the inner and outer sides of the bending waveguides, the bending radius is increased. For the loss in the coupling region, it can be reduced by reducing the coupling efficiency. Additionally, the reduction of the coupling efficiency enables the device to resonate in the under-coupling state, which is helpful to obtain a larger *Q* value.

**Fig. 3:**
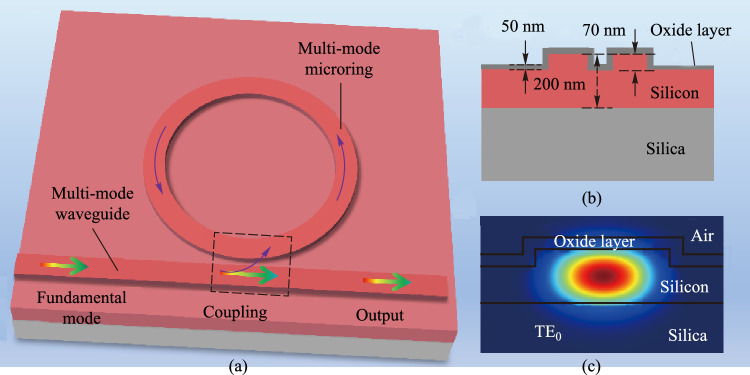
**a** Schematic diagram of the 3D structure of the proposed multi-mode MRR. **b** Cross section of the coupling area. **c** Fundamental mode in ridge waveguide

**Fig. 4:**
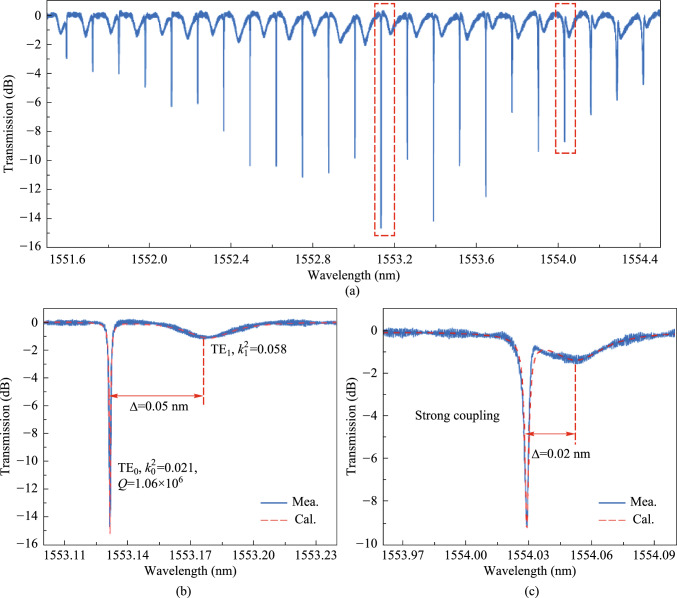
**a** Typical transmission spectrum of the resonant cavity. **b** and **c** show the spectral response at the resonance peaks of weak coupling and strong coupling, respectively

According to the above analysis, we demonstrate an ultra-high *Q* MRR. As shown in Fig. 3, MRRs with ridge waveguide widths of 1.2 μm and 1.5 μm were fabricated by an optimized process. The transmission spectrum of an all-pass MRR with a ridge width of 1.2 μm is shown in Fig. 4a. Two different modes are identified based on the measured resonance peaks in the MRR cavity, as shown in Fig. 4b and c. By analyzing the FSRs of the two modes, it can be confirmed that the fundamental mode and the first-order mode are oscillating in the MRR. Moreover, the distance between the two resonance peaks also changes periodically due to the Vernier effect, and there is an obvious fluctuation envelope between the two resonance peaks.

**Fig. 5:**
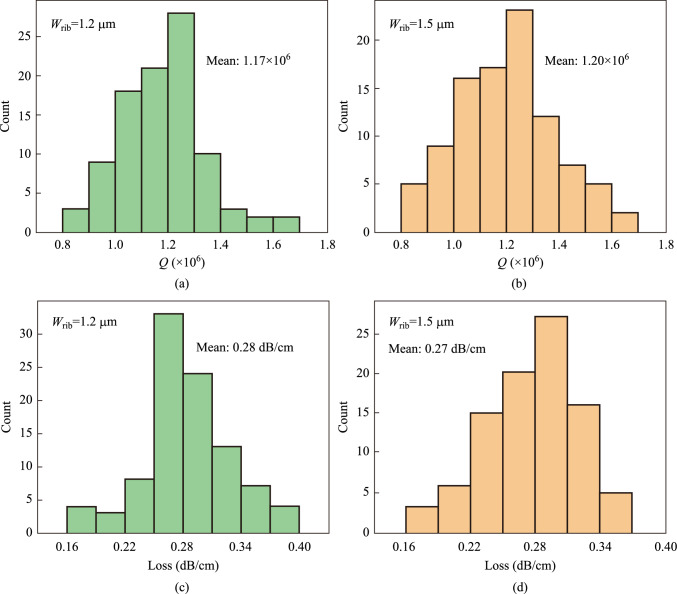
*Q*-factors and waveguide loss statistics of ultra-high *Q* MRRs in the wavelength range of 1500–1580 nm

To increase the accuracy and reliability of the test results, the *Q* value of the resonance peak and the corresponding waveguide loss in the range of 1500–1580 nm are calculated. As shown in Fig. 5, for the MRR with two waveguide widths, the average *Q* values are 1.17 × 10^6^ and 1.20 × 10^6^, respectively, and the average losses are 0.28 dB/cm and 0.27 dB/cm, respectively.

**Fig. 6:**
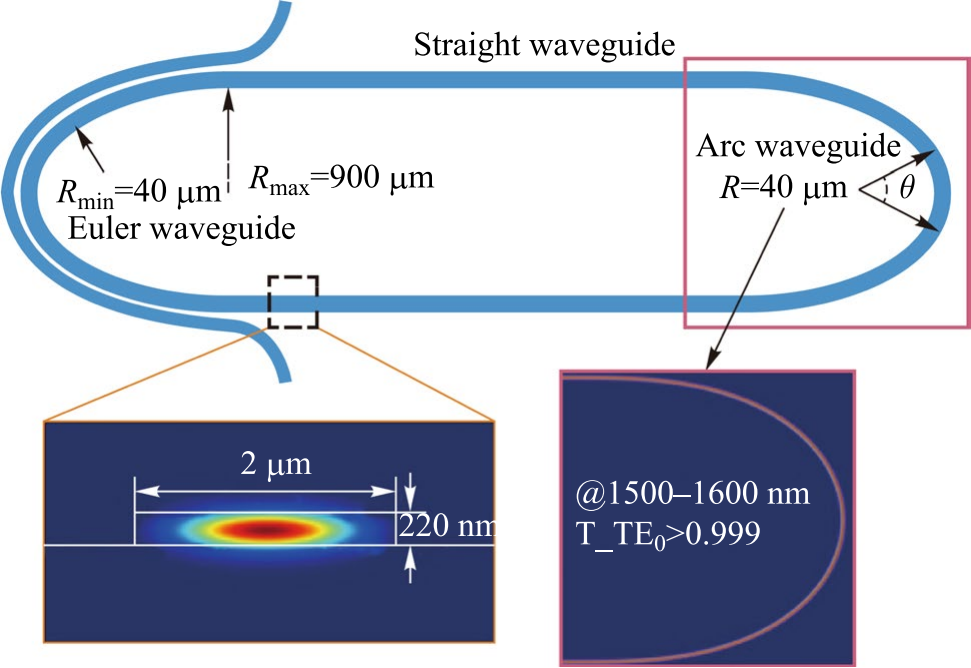
Micro-racetrack resonator using Euler waveguide as transition waveguide

To improve the practicability of the MRR, reducing the size of the device and suppressing the excitation of high-order modes are necessary. We designed a micro-racetrack resonator. The waveguide width of the ring is 2.0 μm and the length of the straight waveguide is 500 μm. The straight waveguide and the arc waveguide cannot be connected directly, which will lead to the mode mismatch effect due to the different curvature. To suppress this effect, we used a part of the Euler linear waveguide, whose radius varies from 900 to 40 μm, as the transition between the straight waveguide and the arc waveguide. The device size is 683 μm × 112 μm, which is 1/25 of the size of the previous ring resonator. The simulation results show that for the wavelength range of 1500–1600 nm the fundamental mode is well-guided over the straight waveguide and the bending waveguide, as shown in Fig. 6. Meanwhile, to suppress the excitation of higher-order modes in the coupling region, two concentric circular arc waveguides satisfying the mode matching are used to avoid the coupling between higher-order modes and fundamental mode.

**Fig. 7:**
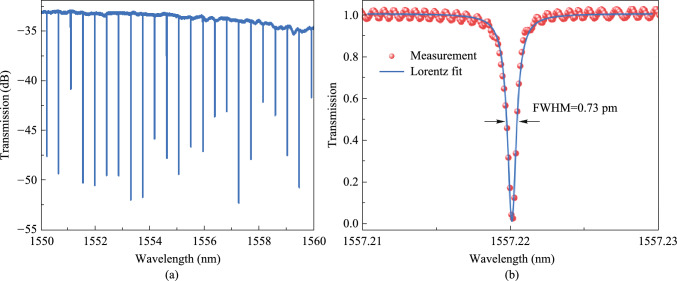
**a** Transmission spectrum of the ultra-high *Q* micro-racetrack resonator. **b** Resonance peak spectrum and fitting curve near 1557.22 nm

The transmission spectrum of the micro-racetrack resonator is shown in Fig. 7a. The device has only the fundamental mode resonance peak, while the high-order mode is not excited. Figure 7b shows a resonant peak located in the C-band, with an extinction ratio of 20 dB, a full width at half maximum of 0.73 pm, a loaded *Q* of 2.1 × 10^6^, an intrinsic *Q* of 3.9 × 10^6^, and a waveguide loss of 0.17 dB/cm.

Table 1 shows the comparison between the optimized ultra-high *Q* MRRs and state-of-art research results. Based on the comprehensive comparison of *Q* factors, extinction ratio, FSR and waveguide loss, the performance of the ultra-high *Q* micro-racetrack resonator based on the multimode waveguide is excellent. The ultra-high *Q* filter output is achieved with a large extinction ratio and a large FSR.

**Table 1:** Parameters comparison of ultra-high *Q* MRRs

Refs.	*Q* (× 10^6^)	ER (dB)	FSR (nm)	Loss (dB/cm)
[56]	1.70	20	0.17	N.A
[86]	1.16	9.0	0.21	0.21
[57]	1.27	4	0.9	0.3
[87]	9.4	1.48	0.33	0.065
[88]	1.40	20	0.24	0.30
This work	2.13	18	0.44	0.17

#### Integrated photonic filters with widely tunable bandwidth and free spectral range

**Fig. 8:**
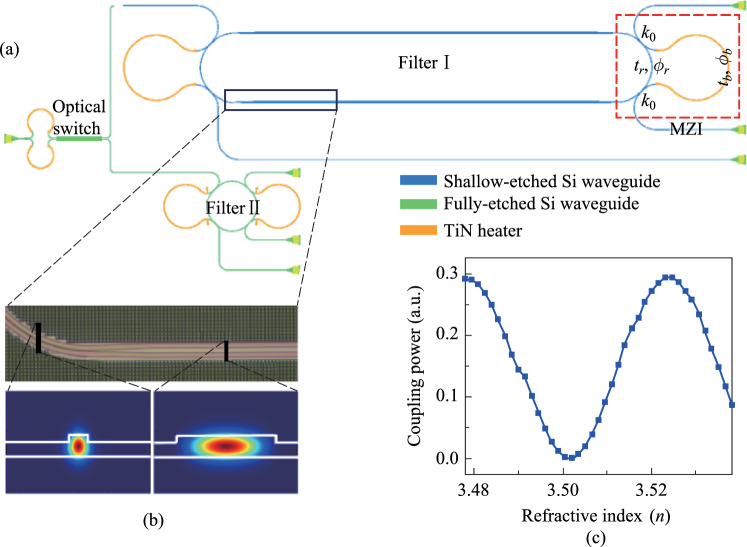
**a** Schematic configuration of the proposed bandwidth-tunable filter. **b** Optical microscopy photo of the taper section connecting single-mode bend waveguide and racetrack waveguide. Two insets show the simulated electric field distribution of fundamental TE mode at the points indicated by black lines. The width of the waveguide at two points are 650 nm and 4 μm, respectively. **c** Simulation results for the coupling power as a function of refractive index of MZI arm, which indicates the variation of the effective coupling coefficient

As illustrated in Fig. 8a, the wide range bandwidth reconfigurable function is achieved through two individual devices with different bandwidth tuning ranges, i.e., Filter I and Filter II. Two devices are connected by an optical switch to switch the optical path between them. The optical switch consists of a 1 × 2 multimode interferometer (MMI), a 2 × 2 MMI, and a pair of MZI arms with equal lengths connecting these two MMIs. The optical energy harvested from the two output ports of the optical switch can be continuously regulated by thermally tuning the optical phase of MZI arms through thermo-optics (TO) effect. Filter I realizes a continuous bandwidth tuning range from about 100 MHz to 2 GHz, while Filter II achieves a tuning range from around 2 GHz to more than 80 GHz. Filter I is based on an ultra-high-*Q* silicon racetrack resonator and Filter II is based on a regular silicon MRR. MZI-assisted schemes are implemented into Filter I and II to achieve the bandwidth tunable function. That is, the straight bus waveguides in the traditional add-drop MRRs are substituted by a pair of MZI arms which yields unequal lengths with the MRR loop section between the two coupling points and further build a pair of MZI structure.

Via the TO effect, the effective coupling coefficient the MZI structure (i.e., red dashed region shown in Fig. 8a) can be continuously tuned by changing the phase difference between the two MZI arms. The effective coupling efficiency *k*, relative phase difference Δ*Φ*, and full width at half-maximum (FWHM) of the resonance spectrum can be calculated as:

$$k^{2} = k_{0}^{2} \left( {1 - k_{0}^{2} } \right)\left( {t_{b} + t_{r} - 2\sqrt {t_{b} t_{r} } \cos \left( {\Delta \Phi } \right)} \right),$$

$$\Delta \Phi = \frac{{2\uppi n_{eff} \Delta L}}{\lambda } = \Phi_{b} - \Phi_{r} ,$$

1 $${\text{FWHM}} = \lambda^{2} \frac{{1 - tt^{\prime}\alpha }}{{\uppi n_{g} L_{rt} \sqrt {tt^{\prime}\alpha } }} = \frac{\lambda }{Q},$$

where *k*_0_ represents the amplitude coupling coefficient of the coupling region; *t*, *t′*, and *k*, *k′* are the bar/cross-coupling coefficients between the two bus waveguides and the MRR, respectively (*k*^2^ + *t*^2^ = 1, *k′*^2^ + *t′*^2^ = 1); ΔΦ is the relative phase difference between the MZI arm and MRR loop; *λ* is the wavelength of light; *n*_*eff*_ and *n*_*g*_ are the effective index and group index of MRR loop, respectively; Δ*L* is the length difference between the two interfering arms of the MZI; and *t*_*b*_, *t*_*r*_, and *ϕ*_*b*_, *ϕ*_*r*_ are the transmission coefficient and phase of the MZI arm and MRR arm, respectively. As ΔΦ is changed from 0 to π, the effective coupling efficiency *k* is continuously changed accordingly from 0 to 4*k*_0_(1 − *k*_0_), which means the *Q*-factor can be continuously tuned from intrinsic* Q*-factor to loaded-*Q* factor, thus realizing the bandwidth tunability [89–91]. We simulated the efficiency of the MZI structure with the change of the refractive index of the MZI arm. The variation of the effective coupling coefficient with the change of the MZI arm refractive index is simulated in 3D FDTD solutions and shown in Fig. 8c. The effective coupling efficiency can be changed dynamically with tunable interferometric couplers and is approximately a sine function with the varied refractive index of the MZI arm. Notably, near-zero coupling efficiency can be obtained through thermal tuning, which means that MZI schemes can be used to approach the intrinsic *Q*-factors of MRR. Furthermore, considering the fact that the effective coupling efficiency *k* is positively linearly correlated with *k*_0_, *k* is pursued to be large enough to provide low loaded *Q*-factors thus large bandwidth tuning range.

Single-mode silicon waveguides have been applied to the bending region of the MRR to ensure purely fundamental modes transmitting, thus avoiding multimode interference and crosstalk. For the racetrack resonator in Filter I, the radius of the bending section is designed to be 100 μm, in order to minimize the mode mismatch loss to a negligible level when the width is set to be 650 nm. To obtain ultra-high-*Q* MRRs, multimode waveguides with a width of 4 μm are introduced into the MRR loop’s racetrack. Based on the electric field distribution of single-mode waveguide and multimode waveguide, as shown in Fig. 8b, the optical mode of the multimode waveguide is more tightly confined in the middle of the waveguide than that of the single-mode waveguide. In the multimode waveguide structure, less overlap area between the optical mode and the sidewall of the waveguide has been realized. Furthermore, the device is fabricated on a shallow-etched 70 nm silicon waveguide, which contributed to the optical mode partially locating in the etch-less slab waveguide region. Both ways can significantly reduce the Rayleigh scattering loss, which is the primary contributor to the cavity round-trip loss in an MRR, caused by the rough sidewall during fabrication. The length of the multimode waveguide racetrack is set as 3500 μm to effectively reduce the overall round-trip loss of the MRR filter and significantly improve the *Q*-factor. To provide adiabatic transmission, linear tapers are used to connect the racetracks and bend sections with a length of 100 μm. The gap between the bending region and the MZI arm is designed to be 400 nm, and the coupling length of the formed coupler is set as 8 μm to achieve a large bandwidth tunable range. For the regular MRR in Filter II, the radius of MRR is set as 50 μm and is fabricated in fully-etched silicon platform for the release of high-*Q* performance. The gap between the bending and the MZI arm is designed to be 180 nm, and the coupling length of the formed coupler is set as 15 μm to achieve a bandwidth tunable range as large as possible. For both Filter I and II, a pair of TiN heaters are positioned on both arms of the MZI with a width of 2 μm to precisely control the phase difference. It is worth noting that another MZI is used to maintain a high extinction ratio (ER) during the bandwidth turning for the filter. This is done by thermally changing the coupling coefficients of two MZI arms simultaneously to maintain the critical-coupling condition.

**Fig. 9:**
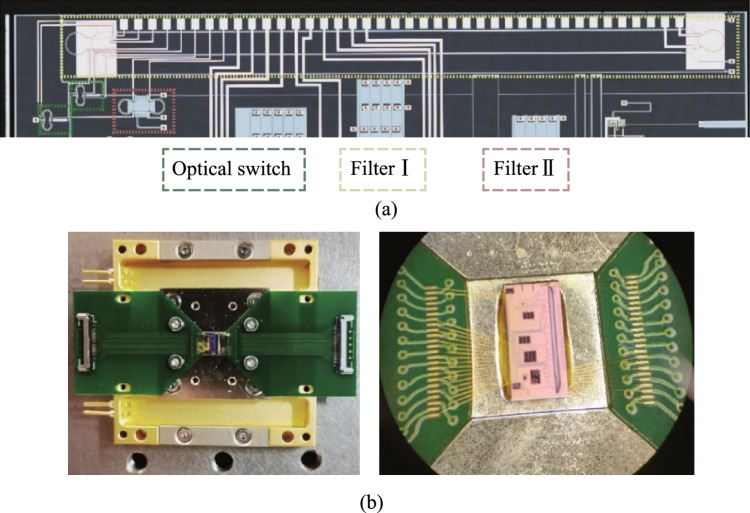
**a** Micrograph of the whole chip. **b** Chip after electrical packaging

**Fig. 10:**
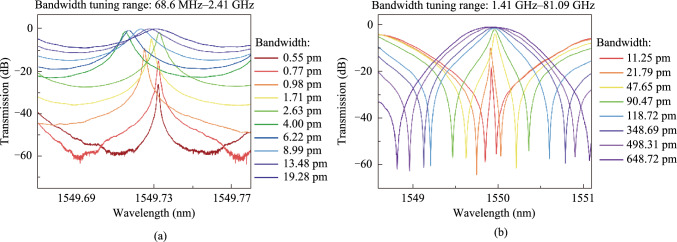
Transmission spectra with different bandwidth of **a** Filter I, and **b** Filter II

The device is fabricated in Chongqing United Microelectronic Center (CUMEC) using a standard Multi-project Wafer (MPW) process based on a 220 nm silicon layer and 2 μm cladding silica layer. Figure 9a shows the microscope picture of the fabricated tunable filter. The chip is electrically packaged as shown in Fig. 9b. The scanning electron microscope images of the device details are not available due to the thick silica cladding. We then experimentally characterized the bandwidth tunability of the fabricated device. A vertically designed grating coupler was used to inject the input light from a tunable laser (Santec TSL 770) after it had been properly polarized into a TE polarization. A power meter (Santec MPM 210) collected the optical transmission spectrum from the output port. Employing probe stations and metal pads, two source meters (Keithley 2400) were used to power the heaters on top of the two MZI arms. We first adjust the optical switch to the optical path of Filter I to measure the transmission spectra. Due to the ultra-high *Q*-factor of the silicon racetrack resonator, the wavelength step of the input light is set to be 0.1 pm, limited by the resolution of the tunable laser source. We then tune the coupling coefficient by applying independent voltages to the heaters on two MZI arms to realize bandwidth tunability. Two source meters driving the two heaters are regulated simultaneously to maintain critical-coupling conditions. Figure 10a shows the transmission spectra with different bandwidths (FWHM) under different regulating voltages, with a tuning range from 0.55 pm (68.6 MHz) to 19.28 pm (2.41 GHz). Heating voltages, extinction ratios, and insertion losses of some transmission spectra with different bandwidths are summarized in Table 2. We then adjust the optical switch to the optical path of Filter II to characterize its bandwidth tunability, and the transmission spectra are shown in Fig. 10b with different bandwidths. Our fabricated Filter II shows a bandwidth tuning range from 11.25 pm (1.41 GHz) to 648.72 pm (81.09 GHz) under different regulating voltages. Details for some transmission spectra can be found in Table 3. There exists about 1 GHz bandwidth overlap between Filter I and Filter II. In summary, the bandwidth of proposed filter can be continuously tuned from 0.55 pm (68.6 MHz) to 648.72 pm (81.09 GHz).

**Table 2:** Details of typical transmission spectra of Filter I

Heating voltage of two MZIs	Bandwidth (pm)	Bandwidth	Extinction ratio (dB)	Insertion loss (dB)
5.90 V, 13.63 V	0.55	68.64 MHz	31.3	26.7
6.10 V, 13.54 V	0.77	96.10 MHz	44.3	15.7
2.80 V, 8.30 V	13.48	1.68 GHz	11.16	0.48

**Table 3:** Details of typical transmission spectra of Filter II

Heating voltage of two MZIs	Bandwidth (pm)	Bandwidth (GHz)	Extinction ratio (dB)	Insertion loss (dB)
3.10 V, 2.71 V	11.25	1.40	40.7	18.3
3.40 V, 2.12 V	21.79	2.72	50.0	10.0
2.80 V, 8.30 V	648.72	80.96	60.0	0.2

#### FSR programmable photonic filter chip

**Fig. 11:**
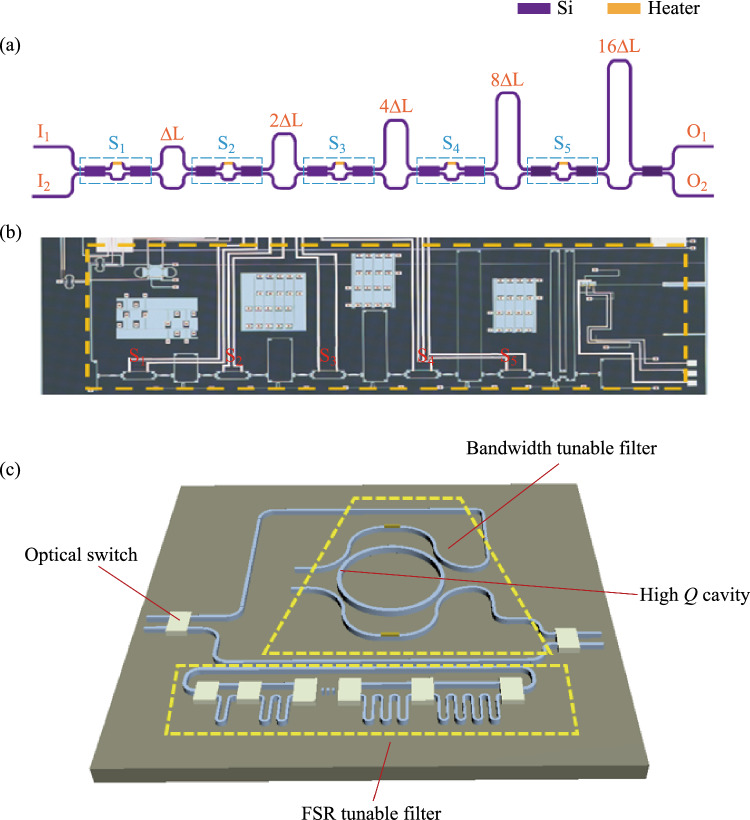
**a** Schematic diagram of the FSR programmable photonic filter. **b** Optical microscope image of the fabricated device. **c** Schematic diagram for the whole chip including high-*Q* MRR, bandwidth tunable filter and FSR tunable filter

We propose an FSR programmable photonic filter chip on SOI platform in Fig. 11a, which can realize FSR-reconfigurable function. It consists of five MZIs with different arm length differences and five optical switches (S_1_, S_2_, S_3_, S_4_, S_5_). The arm length differences of the interference structures are ΔL, 2ΔL, 4ΔL, 8ΔL, and 16ΔL from left to right, respectively. This filter can be regarded as a large Mach–Zehnder interference structure with variable overall length differences. In this case, the FSR of the filter can be expressed as

2 $${\text{FSR}} = \frac{{\lambda^{2} }}{{n_{g} \Delta L_{total} }},$$

where *λ* is the center wavelength of interest, ∆*L*_*total*_ is the overall length difference between long and short path of the filter and *n*_*g*_ is the group index of the designed waveguide around the center wavelength. For a SOI waveguide with cross section of 220 nm × 450 nm, *n*_*g*_ is about 4.28 at C band. When we set the basic arm length difference ΔL = 301.75 μm, the free spectral range of the corresponding MZI-type filter is 1.86 nm. In practice, each optical switch has three states: the “Cross” state (C), the “Bar” state (B), and the “Middle” state (M). The so-called “Middle” state is the situation in which the power of the two ports of the optical switch is balanced, that is, 50% each. There is an auxiliary test port for each optical switch that taps a small amount of light for calibrating the state of the optical switch. Figure 11b shows the microscope image of the FSR programmable photonic filter, which is incorporated on the same chip with the previously bandwidth-tunable filter.

The combinations of optical switching states can generate 31 discrete FSR values, as illustrated in Table 4. According to Eq. (2), the FSR value is inversely proportional to the total arm length difference. When the total arm length difference is the smallest value ΔL, the free spectral range is the greatest value FSR_0_, and when the total arm length difference is the greatest value 31ΔL, the free spectral range is the smallest value FSR_0_/31. We adopt the transmission matrix method to compute the spectral responses of the filter.

**Table 4:** States of optical switches for 31 FSR reconfigurable values of the integrated photonics filter (B: “Bar” state; C: “Cross” state; M: “Middle” state)

No.	S_1_	S_2_	S_3_	S_4_	S_5_	Overall length difference
1	M	B	B	B	C	ΔL
2	C	M	B	B	C	2ΔL
3	M	C	B	B	C	3ΔL
4	C	B	M	B	C	4ΔL
5	M	C	C	B	C	5ΔL
6	C	M	C	B	C	6ΔL
7	M	B	C	B	C	7ΔL
8	C	B	B	M	C	8ΔL
9	M	B	C	C	C	9ΔL
10	C	M	C	C	C	10ΔL
11	M	C	C	C	C	11ΔL
12	C	B	M	C	C	12ΔL
13	M	C	B	C	C	13ΔL
14	C	M	B	C	C	14ΔL
15	M	B	B	C	C	15ΔL
16	C	B	B	B	M	16ΔL
17	M	B	B	C	B	17ΔL
18	C	M	B	C	B	18ΔL
19	M	C	B	C	B	19ΔL
20	C	B	M	C	B	20ΔL
21	M	C	C	C	B	21ΔL
22	C	M	C	C	B	22ΔL
23	M	B	C	C	B	23ΔL
24	C	B	B	M	B	24ΔL
25	M	B	C	B	B	25ΔL
26	C	M	C	B	B	26ΔL
27	M	C	C	B	B	27ΔL
28	C	B	M	B	B	28ΔL
29	M	C	B	B	B	29ΔL
30	C	M	B	B	B	30ΔL
31	M	B	B	B	B	31ΔL

**Fig. 12:**
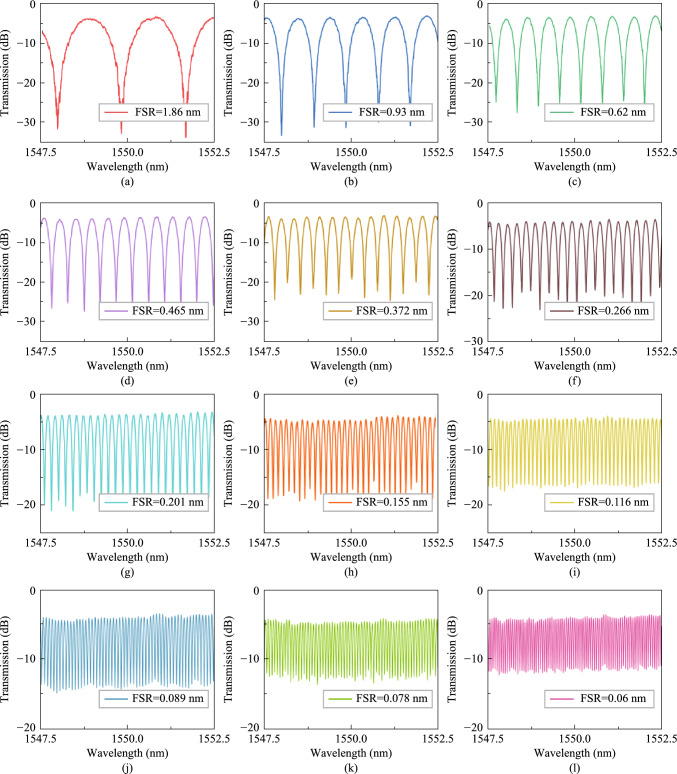
Measured transmission spectra of the tunable and reconfigurable optical notch filters based on MZIs when **a** FSR = 1.86 nm, **b** FSR = 0.93 nm, **c** FSR = 0.62 nm, **d** FSR = 0.465 nm, **e** FSR = 0.372 nm, **f** FSR = 0.266 nm, **g** FSR = 0.201 nm, **h** FSR = 0.155 nm, **i** FSR = 0.116 nm, **j** FSR = 0.089 nm, **k** FSR = 0.078 nm, and **l** FSR = 0.06 nm, respectively

In the experiments, the broad-spectrum light generated from the amplified spontaneous emission (ASE) source is coupled into the device through grating couplers and the output light is fed into a high-resolution optical spectrum analyzer (OSA). The grating couplers are designed for TE polarization with a central wavelength around 1550 nm. The measured spectral responses are normalized concerning the transmission of the reference grating couplers on the same chip. The optical switches in the filter are first calibrated one by one. Then we perform a series of spectral responses tests. Figure 12 shows the measured transmission spectra of the integrated photonic filter under different combinations of optical switching states. Each state is adjusted according to Table 4 as light enters the input and exits the output. Following this, the spectra corresponding to Fig. 12a can be acquired at the output, and the spectra of all the 31 scenarios can be obtained in the same manner. For the ease of demonstration, we select 12 representative spectral lines from 31 states. The FSR values achieved with this filter shown in Fig. 12a–l are 1.86 nm, 0.93 nm, 0.62 nm, 0.465 nm, 0.372 nm, 0.266 nm, 0.201 nm, 0.155 nm, 0.116 nm, 0.089 nm, 0.078 nm, 0.06 nm, respectively. As a result, the FSR reconfigurable range is 0.06 nm–1.86 nm, corresponding to a frequency range of 7.5 GHz–232.5 GHz. The experimental reconfigurability results imply that the filter can obtain FSR reconfigurability in frequencies beyond 200 GHz. The device performance will be further enhanced if the waveguide transmission loss and the non-equilibrium of the optical switch can be reduced.

### Conclusion

In conclusion, we have demonstrated a high-*Q* micro-racetrack filter with a loaded *Q* of 2.1 × 10^6^, a bandwidth-tunable MZIs-assisted MRR filter which could be tuned from 0.55 pm (68.6 MHz) to 648.72 pm (81.09 GHz) and a cascaded MZIs filter with an FSR reconfigurable range from 0.06 nm (7.5 GHz) to 1.86 nm (232.5 GHz). The bandwidth tunable filter and the FSR tunable filter are integrated in a single chip with its schematic diagram given in Fig. 11c. Table 5 summarizes the state-of-the-art reconfigurable photonic filters in terms of *Q*, bandwidth, and FSR reconfigurability. As can be seen, our work represents the first demonstration of an integrated and switchable photonic filter with all these functions together.

**Table 5:** Performance of the reconfigurable photonic filters

Refs.	Switchable	*Q* (× 10^6^)	Bandwidth tuning range	FSR tuning range	Integrate function	Technological route	Year
[87]	No	9.4	–	–	High-*Q*	SOI; Racetrack Euler resonator	2022
[92]	No	2.2	–	–	High-*Q*	SOI; Multimode racetrack resonator	2023
[91]	No	–	240 MHz–1.375 GHz		Bandwidth tunable	SOI; MZI assisted multimode racetrack resonator	2021
[75]	No	–	11.6 GHz–125 GHz		Bandwidth tunable	SOI; MRRs	2013
[93]	No	–	–	2.5 GHz–15 GHz	FSR tunable filter	III–V laser; Comb injection locked filter	2020
This work	Yes	2.1	68.64 MHz–80.96 GHz	7.5 GHz–232.5 GHz	High-*Q*, bandwidth tunable, FSR tunable	SOI; Multimode racetrack resonator and cascaded MZIs	2023

## Reconfigurable logic operation photonic chip

*Xiaoyan Gao, Wentao Gu, Wenchan Dong, Lei Lei, Xinliang Zhang*.

### Current status

In recent years, high-performance optical computing has been widely noticed as a hotspot at the forefront of science and technology in the world, and has been rapidly developed in the field of artificial intelligence, such as neural networks and deep learning [94]. Digital signal-oriented optical logic operation is one of the difficulties in optical computing [95]. Optical logic operation is mainly divided into three research directions: electro-optical directed logic, all-optical logic and linear interference logic. The ultimate goal is to realize a low-power, high-speed, programmable logic operation chip.

Optical computing scheme based on electro-optical logic has gradually gained attention since Lin Yang’s group at the Institute of Semiconductors, Chinese Academy of Sciences, had an experimental study in 2010 [96], and researchers have begun to further study the realization of programmable integrated optical computing with complex functions based on the realization of directed logics such as XOR, XNOR, etc. based on the use of micro-ring resonator (MRR) or micro-disks resonator (MDR) as optical switches [97]. In 2015 Tian et al. from Lanzhou University proposed and experimentally demonstrated a scheme to realize multiple electro-optical logic using wavelength-division multiplexing (WDM) based on MRR optical switch arrays. The scheme extended single-wavelength continuous light into multi-wavelength continuous light, and combined multiple micro-rings to modulate different wavelengths of light respectively to produce different combinations of logic minterms, thus realizing reconfigurable logic functions [98, 99]. To reduce the power consumption of the electro-optical logic switch, Ray Chen’s group at the University of Texas at Austin, USA, conducted a detailed comparison between MRR-based and MDR-based switches in terms of size, power consumption, and fabrication tolerances, and concluded that MDR-based switch arrays have a significant advantage over MRR-based switches in logic computation in terms of footprint and power consumption [100]. The group proposed to complete complex logic operations in the electrical domain and load the calculated logic signals onto light for transmission through electro-optical modulators to realize different logic functions. In 2018 the group experimentally demonstrated 2.5 Kb/s full adder using a silicon-based micro-disk modulator in a single wavelength [101], and subsequently implemented 20 Gb/s 4-bit digital logic operator [102], three-input switching network [103], and optical comparator functionality [104] by introducing WDM and optimizing the modulation rate of the modulator, respectively. This electro-optical logic scheme utilizes only light as the transmission medium and has good scalability, which is mainly limited by the operation rate of the electro-optical modulator. On the one hand, the electro-optical logic schemes can play an auxiliary role in schemes based on nonlinear effects, and on the other hand, they can also take advantage of the multi-dimensional parallelism of optical signals to increase the computational capacity of optical logic computing systems.

The programmable optical computing scheme based on all-optical logic is to carry out inter-signal interactions in the optical domain directly, and utilizes control light with higher power to change the refractive index of the nonlinear medium, thus changing the information carried by the signal light and output logic operations. All-optical logic operation can avoid the additional energy consumption and bottlenecks of operation speed that exist in electro-optical logic schemes. The core idea of programmable optical logic operation scheme is to take the full set of logic minterms as the basic building blocks (BBBs), and any complex logic function can be obtained based on the BBBs [105, 106]. The programmable optical computing system mainly includes a minterm circuit and a switching circuit. The former is used to generate a full set of minterms, the latter is used to realize the programmable control of the minterms, so as to diversify the output logic results to meet the user’s needs. In 2010 Lawrence R. Chen team from McGill University proposed to realize 10 Gb/s reconfigurable logic gates based on the cross phase modulation (XPM) effect in highly nonlinear fiber (HNLF) [107]. In 2013, Wada et al. from Heriot-Watt University, experimentally demonstrated the simultaneous utilization of cross-gain modulation (XGM) and four-wave mixing (FWM) effects in semiconductor optical amplifier (SOA) to realize several canonical logic units (CLUs) and then constructed half-adder and half-subtractor based on them [108]. In the text, CLUs is referring to logic minterms. Chen et al. from Tsinghua University proposed and experimentally demonstrated several CLUs based on the FWM, XGM, and XPM effects in single SOA [109]. In 2020, the scheme of constructing an all-optical two-bit multiplier based on different types of CLUs using multi-channel FWM was proposed [110], and then a three-input programmable logic array (PLA) based on a full set of CLUs in a single nonlinear device was experimentally demonstrated [111]. These schemes fully show the rich nonlinear effects available in nonlinear devices such as SOA and HNLF. Taking advantage of the parallelism of light and enriching the functionality of a single logic device by using various nonlinear effects to achieve the expansion of the computational capacity of the logic system, which shows the most significant advantage of using optical signals instead of electrical signals to realize logic functions.

Regarding to the integrated all-optical logic schemes, Hou et al. from Huazhong University of Science and Technology (HUST) generated two-input CLUs based on FWM in silicon-based waveguide [112]. To improve the integration of programmable optical computing systems, a 40 Gb/s monolithic integrated programmable logic scheme including minterm circuit and switching circuit based on SOAs in indium phosphide platform was proposed [113]. The scheme introduces on-chip programmable control, heralding a big step closer to the integration of smart computing with flexible and programmable all-optical computing schemes. However, limited by the carrier recovery time of SOA, the scheme is designed not to generate the full set of four minterms simultaneously, making it difficult to realize arbitrary function outputs. The key issue of the current all-optical logic integrated scheme is to enhance the nonlinear effects of the integrated devices, which can enrich the computing function of a single device and reduce the required power consumption of the system.

Linear interference-based programmable logic aims at designing general-purpose photonic networks to realize various basic and complex functions in many application areas. Notable properties of programmable photonic networks include flexibility to reshape limited resources, robustness and resilience, rapid system creation through time-sharing, and unlimited resources [94]. Basically, a programmable photonic network is formed by cascading a large number of 2 × 2 BBBs, including Mach–Zehnder interferometer (MZI), MRR, directional coupler (DC), optical switch based on three waveguides with central nanobeam [114, 115]. Prof. Shamir has proposed a generalized programmable optical network, for performing reconfigurable and reversible logic operations [116]. The unidirectional transportability of this network allows the results can be obtained only on one side, thus limiting the simple progressive setup of the forward only mesh network. It allows the system to minimize or maximize the power of the photodetectors, or in some cases, to self-configure or self-stabilize for specific problems [117, 118]. 2017 MIT researchers Shen et al. present a programmable nanoscale processor that utilizes programmable 56 MZIs in silicon photonic integrated circuits to implement a new architecture for all-optical neural network and demonstrate recognition of vowel sounds [119]. This programmable integrated optical operation, which mainly processes analog signals, flexibly realizes different linear operations by adjusting the parameters of each component through external algorithms.

### Challenges and solutions

As mentioned previously, the operation rate of electro-optical logic is limited by the bandwidth of electro-optical conversion, and linear interference logic mainly processes analog signals, it is difficult for these two solutions to realize high-speed computing based on digital signals. The all-optical logic system has advantages of high-speed and parallelism. However, the current schemes with simple functions and insufficient re-configurability cannot meet the needs of optical computing and optical processing systems. Specifically, all-optical logic devices face the following challenges.

Firstly, all-optical logic devices have simple functionality and insufficient reconfigurability. Single-function optical logic gates have been fully proposed and characterized at high rates. Meanwhile, some schemes have been proposed for realizing reconfigurable logic gates, such as using different nonlinearities in the single device to realize different logic gates. However, the lack of consistency in the logic results produced by the different nonlinear effects degrades the quality of power-coupled arbitrary logic functions based on these logic results. Besides, limited by the system structure based on nonlinearities, it is difficult to realize a full set of CLUs to combine arbitrary logic function.

For the realization of optical arbitrary logic functions, programmable logic array based on canonical logic units (CLUs-PLA) is proposed. The CLUs-PLA includes three parts, input circuit, CLUs array, and coupling array [120]. Input circuit consist of delay interferometer (DI) to generate optical signals with complementary codes. CLUs array is used to generate a full set of CLUs simultaneously, which is the BBBs to construct arbitrary logic function. The coupling array is used to couple these CLUs selectively according to users’ need. Thus, the scheme of CLUs-PLA can output programmable logic operations based on the simultaneous generation of a full set of CLUs.

Secondly, the nonlinearity of integrated devices is insufficient. On the one hand, the limited number of logic results based on a single integrated device will limit the computational capacity of the computing system; on the other hand, the lack of nonlinearity will deteriorate the signal quality of the output logic results, hindering the computing performance of the system at high modulation rate. Therefore, it is necessary to improve the nonlinearity of the integrated devices.

To generate high-speed CLUs results with better quality, the nonlinearity of integrated devices needs to be further enhanced, thus various schemes based on different materials and structures have been proposed. The basic solution is to optimize the phase matching condition by design the width and the height of the cross-section of the optical waveguide. The broadband wavelength conversion above 50 nm at 40 Gb/s based on optimized silicon waveguide is demonstrated, showing the improvement of conversion bandwidth by dispersion optimization [121]. Further, when the input optical power in the nonlinear device is increased, the signal quality of CLUs results could be improved to some extent. However, the nonlinear absorptions such as two-photon absorption (TPA) and free-carrier absorption (FCA) are serious limiting factors for optical power. Responding to this issue, sweeping free carriers through a reverse biased PIN junction is proposed, realizing 7-channel all-optical reconfigurable CLUs multicasting at 40 Gb/s [122]. On the other hand, constructing a surface plasmon waveguide or a slot waveguide to enhance the intensity of the optical field in a specific region is also an option, which improves the conversion efficiency of FWM by more than 12 dB and implements the all-optical CLUs at 40 Gb/s [51].

In summary, various schemes are proposed to enhance nonlinearity, but suffer from different limitations, such as the bandwidth limitation brought by resonant devices, the cost increases brought by silicon waveguide with reverse-biased PIN junctions, special processes required by surface plasmon waveguide. How to utilize nonlinear enhancement devices to build high-complexity and highly reconfigurable logic arrays is an urgent problem to be solved.

### Progress

To solve the challenges of insufficient reconfigurability, single function, and low overall integration of functional modules in all-optical programmable logic arrays, we optimize the nonlinear waveguide structure and proposes a variety of nonlinearity-enhanced waveguides for different application scenarios based on theoretical analysis of the standard all-optical programmable logic array model. Based on the designed waveguide, the logic operation function of 100 Gb/s is realized, and the combinational logic functions based on CLUs are demonstrated. Experiments show that the logic conversion bandwidth is greater than 16.1 nm. The logic levels are clearly identified, and the eye diagram opens significantly. Based on the nonlinearity-enhanced chip, the all-optical multi-valued logic operations of 80 Gb/s and 100 Gb/s QPSK signals are realized, and the constellation diagram of logic signals is clear and the logic sequences are correct. An ultra-narrow slot waveguide with 50 nm slot width filled with highly nonlinear polymer MEH-PPV chip was developed. Compared with the structure without polymer at the same length and the traditional silicon-based striped waveguide, the FWM conversion efficiency of this chip has been improved by more than 10 dB and 5 dB, and combined with the pre-coding technique, a full set of CLUs under 40 Gb/s RZ-DPSK signals were realized. Error-free operations are achieved for all logic results (bit error rates below 10^−9^).

#### Monolithic integration of programmable optical logic array

**Fig. 13:**
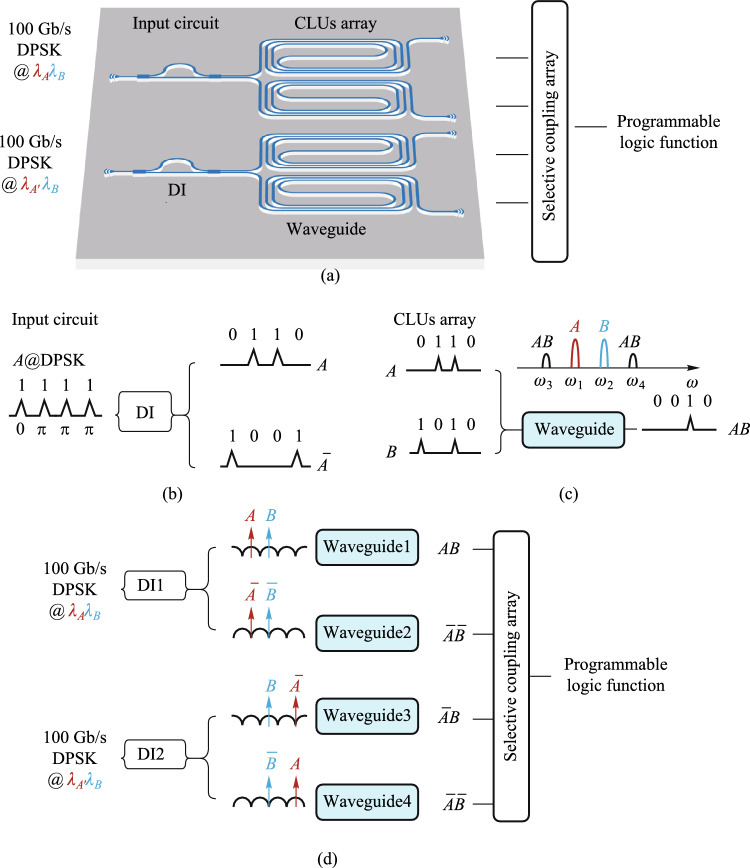
Schematic diagram of **a** the programmable logic array chip based on the full set of CLUs; **b** the input circuit based on DI; **c** the CLUs array based on FWM effect; **d** the implementation of the full set of CLUs

Monolithic integration of programmable optical logic array is proposed based on linear pre-coding and nonlinear four-wave mixing in silicon-based nonlinearity-enhanced chip. The schematic diagram of the PLA chip based on the full set of CLUs is shown in Fig. 13a. The chip consists of the input circuit and the CLUs array. The input circuit includes two delay interferometers (DIs), which is used to demodulate two 100 Gb/s differential phase-shift keying (DPSK) signals to four couples of on–off keying (OOK) signals with complementary codes. The CLUs array includes four nonlinearity-enhanced ridge waveguides to generate CLUs through FWM effect. The principle of input circuit is shown in Fig. 13b. DI demodulates DPSK signals to complementary OOK signals. For the CLU array, two input signals $A$ and $B$ are used as pump signals, and two converted signals carrying the amplitude information of $AB$ are generated through FWM effect in the silicon waveguide, which is shown in Fig. 13c. The principle of the simultaneous generation of the full set of CLUs is described in Fig. 13d. For the upper device, two DPSK signals at wavelengths *λ*_*A*_ and *λ*_*B*_ are demodulated to $A$, $B$ and $\overline{A }$, $\overline{B }$ into two waveguides, generating the AND logic signals of $AB$ and $\overline{A }\overline{B }$. For the lower device, due to signal $A$ has a wavelength shift of *λ*_FSR_/2 (*λ*_FSR_ is the free spectral range (FSR) of the DI), signals of $\overline{A }$, $B$ and $A$, $\overline{B }$ are demodulated into two waveguides, generating $\overline{A}B$ and $A\overline{B }$. Based on the simultaneous implemented of full set CLUs, arbitrary logic functions can be realized by combination of CLUs through optical couplers.

**Fig. 14:**
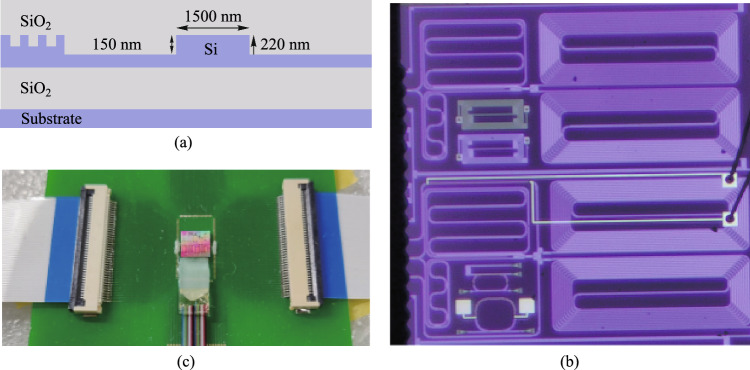
**a** Schematic diagram of the waveguide cross-section. **b** Microscopic image of the fabricated PLA chip. **c** Photo of the encapsulated module

Silicon-based nonlinearity-enhanced waveguides are designed as the ridge waveguides with width of 1.5 µm, etching depth of 150 nm and length of 3 cm. The schematic diagram of the waveguide cross-section is shown in Fig. 14a. The ridge waveguides with large widths have stronger nonlinearities benefiting from their lower linear sidewall scattering loss and lower nonlinear losses due to large effective model area, which enables higher power to participate in nonlinear effects. The linear loss is experimentally measured as 0.33 dB/cm. Euler bending has been introduced to reduce the bending radius of the device, and the phase thermal tuning of DI is introduced to facilitate the experimental test. The PLA chip was fabricated in Chongqing United Microelectronics Center (CUMEC), and the microscopic image of the chip is shown in Fig. 14b. Figure 14c shows the PLA module after packaging the vertically coupled grating array and electrodes, which is used in the 100 Gb/s full set of CLUs generation experiment.

**Fig. 15:**
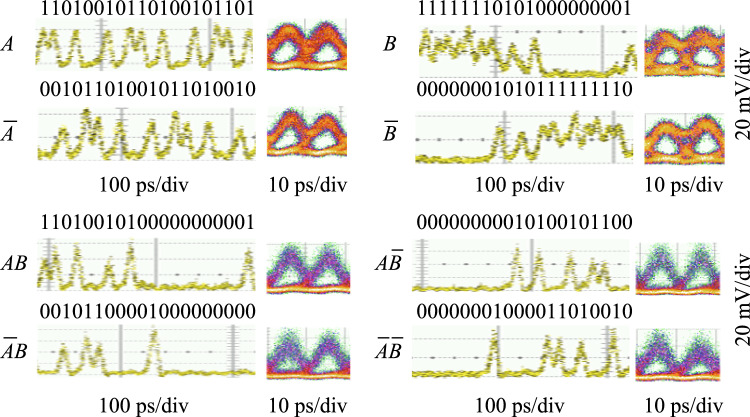
Time-domain waveforms and eye diagrams of the results of PLA chip at 100 Gb/s, including original demodulated signals, $A$, $\overline{A },$ $B$, $\overline{B }$, and CLUs signals, $AB,$ $\overline{A }\overline{B },$ $\overline{A}B,$ and $A\overline{B }$

The time-domain waveforms and eye diagrams of the original demodulated signals and the CLUs signals are given in Fig. 15. $A$ and $\overline{A }$ are a pair of original demodulated signals with complementary code streams; $B$ and $\overline{B }$ are another pair of demodulated signals.$AB,$ $\overline{A }\overline{B },$ $\overline{A}B,$ and $A\overline{B }$ are the full set of the CLUs signals. The logic levels are clearly identified with wide-open eyes and the logic sequences are correct, which proves the high-speed computing capability of the all-optical PLA chip at 100 Gb/s operation rate. By improving the quality of the original 100 Gb/s DPSK signal and reducing the amplitude jitter, the quality of the CLU signal and further arbitrary logic functions can be improved.

Comparison of this work with other nonlinear logic devices is shown in Table 6. As can be seen from Table 6, it is the first 100 Gb/s silicon-based logic computing device to generate the full set of CLUs simultaneously, pushing the silicon photonic circuits toward high-speed optical signal processing and complex optical computing.

**Table 6:** Performance of nonlinear logic devices

Refs.	Single-channel operation rate (Gb/s)	Device	Reconfigurable	Integrated	Arbitrary logic function
[109]	100	SOA	Yes	Yes	No
[112]	40	Si waveguide	Yes	Yes	Yes
[113]	40	SOA	Yes	Yes	Yes
[110]	40	HNLF	Yes	No	Yes
[123]	4	Silicon microresonator	No	Yes	No
This work	100	Silicon PLA	Yes	Yes	Yes

#### High-dimensional multi-value logic operation devices

With increased data capacity, advanced modulation formats have received significant attention due to their advantages of improved capacity and spectrum utilization efficiency. Quadrature phase-shift keying (QPSK) signals with four constellation points can represent two binary bits and have a natural advantage in performing multivalued logic operations. Here, an all-optical multivalued logic operator device based on QPSK signals is proposed, which realizes all-optical multivalued logic operations at 100 Gb/s. The FWM process can be described by the following equation:

3 $$E_{mnk} = K_{mnk} E_{m} E_{m} E_{K}^{*} = K_{mnk} \left| {E_{m} } \right|\left| {E_{n} } \right|\left| {E_{k} } \right|\text{e}^{{\text{j}\left( {\omega_{m} + \omega_{n} - \omega_{k} } \right) + \left( {\theta_{m} + \theta_{n} - \theta_{k} } \right)}} ,$$

where $\omega_{m} ,\left| {E_{m} } \right|,\;{\text{and}}\;\theta_{m}$ correspond to the angular frequency, electric field strength, and phase of light, respectively. $E_{mnk}$ corresponds to the field of the generated idler light, and $K_{mnk}$ is positively proportional to FWM efficiency. The equation shows that the electric field strength of the newly generated idler frequency light is proportional to the product of the three original optical field strengths, and the phase is equal to the sum of the two pump optical phases minus the signal optical phase. Based on the phase property of FWM, the multi-valued logic calculation can be utilized by the FWM process between two QPSK signals.

**Fig. 16:**
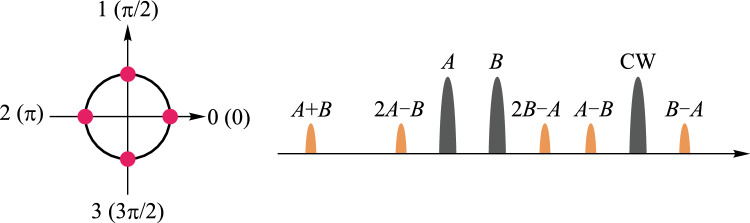
Schematic diagram of all-optical multivalued logic operation based on QPSK signals using FWM

The principle of the all-optical multivalued logic operation of the QPSK signal is illustrated by Fig. 16. The constellation diagram of the QPSK signal is shown on the left side, which is a phase-encoded optical modulation format with four possible phase values for each bit, i.e., 0, π/2, π, and 3π/2, corresponding to “00, 01, 10, 11” in binary, respectively. The right side depicts the phase relationship between the two QPSK signals $A$ and $B$, and the multi-channel idler lights generated by the FWM process follow the previously described phase relationship and carry different operations including $A-B$, $B-A$, and so on.

**Fig. 17:**
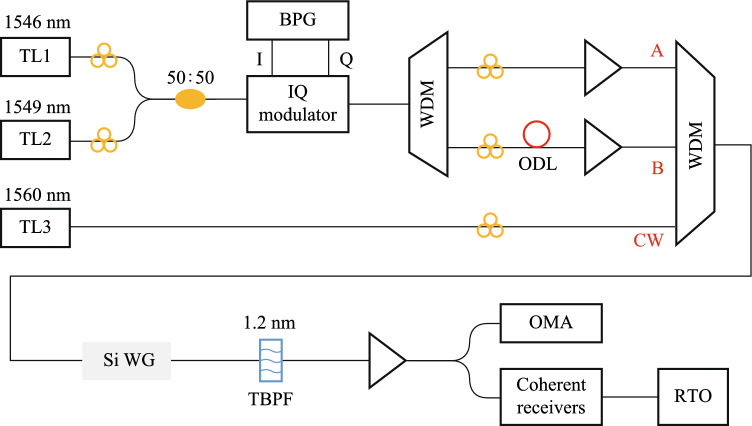
Experimental setup for testing multivalued logic operations

**Fig. 18:**
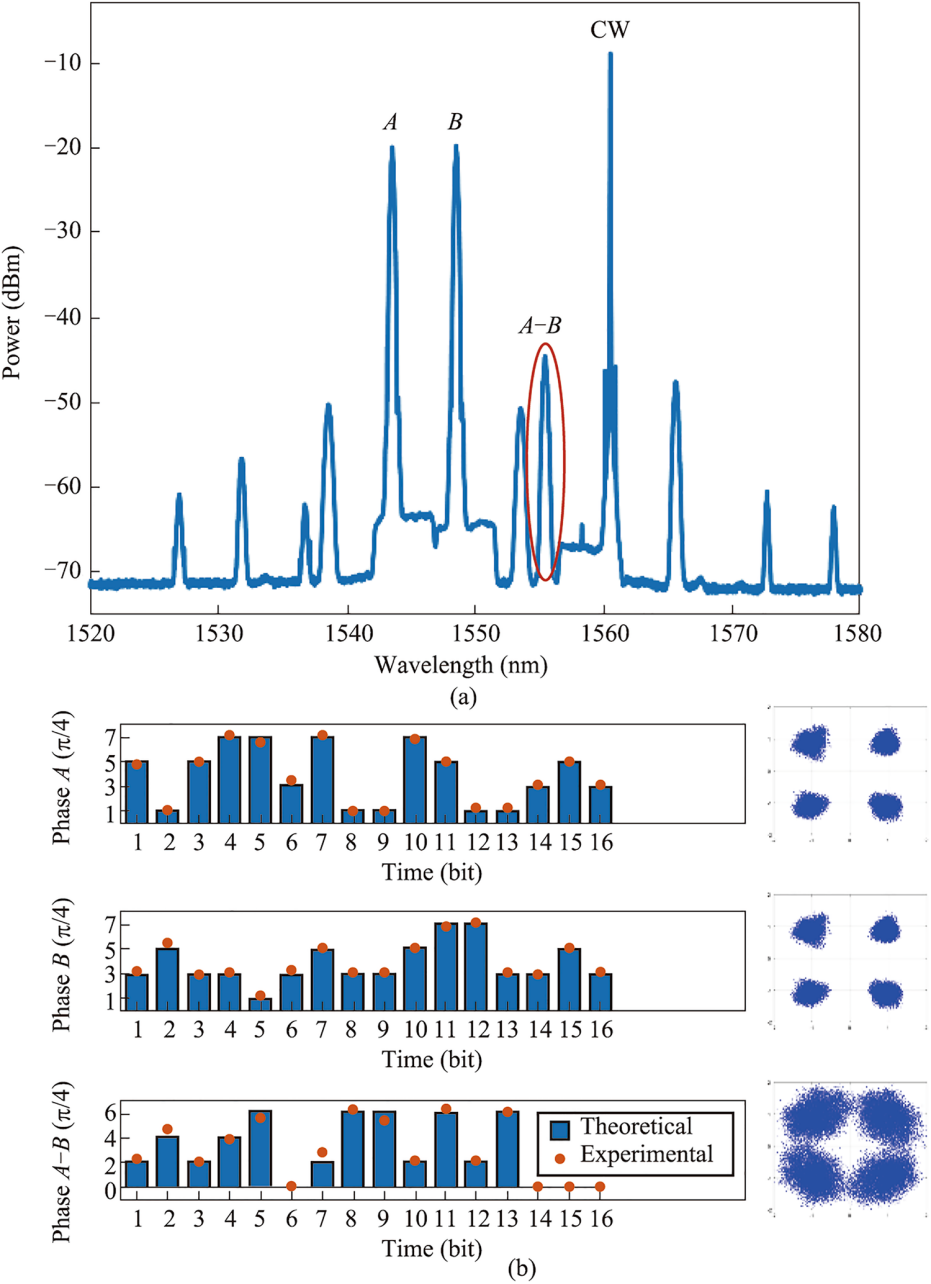
**a** Experimental spectra. **b** Sequences and constellation diagrams of the original signals and the logic signals

The nonlinear medium is selected as the nonlinearity-enhanced waveguide described in the previous section, with width of 1.5 μm and length of 3 cm, as shown in Fig. 14a. The experimental setup for testing multivalued logic operations is shown in Fig. 17. Three lasers emit CW lights with wavelength of 1546 nm, 1549 nm, and 1560 nm, and the first two paths are modulated into QPSK signals by the IQ modulator. After the FWM process in the silicon-based waveguide, idler light is generated at 1557 nm. The two-bit phase subtraction of the original QPSK signal is realized on the idler light. The results of the experiment are displayed in Fig. 18. Figure 18a shows the experimental spectra, and the logic result of $A-B$ is marked. The sequences and constellation diagrams of the original signals and the logic signals are shown in Fig. 18b. It can be seen that the logic signal constellation diagram is clear and the logic results are correct, showing potential of building an all-optical logic integrated computing scheme with high flexibility and high computing rate.

#### Enhanced optical nonlinearity in a silicon–organic hybrid slot waveguide for all-optical signal processing

Current nonlinear devices suffer from limited operation speeds and low conversion efficiencies due to the intrinsically nonlinear absorption of silicon. Here, we experimentally demonstrate enhanced optical nonlinearity in a silicon-organic hybrid slot waveguide consisting of a 45 nm ultra-narrow slot waveguide coated with a highly nonlinear organic material [51]. Based on the nanostructure design, the conversion efficiency of the degenerate four-wave mixing showed enhancements of more than 12 dB and 5 dB compared to those measured for an identical device without the organic material and a silicon strip waveguide, respectively.

**Fig. 19:**
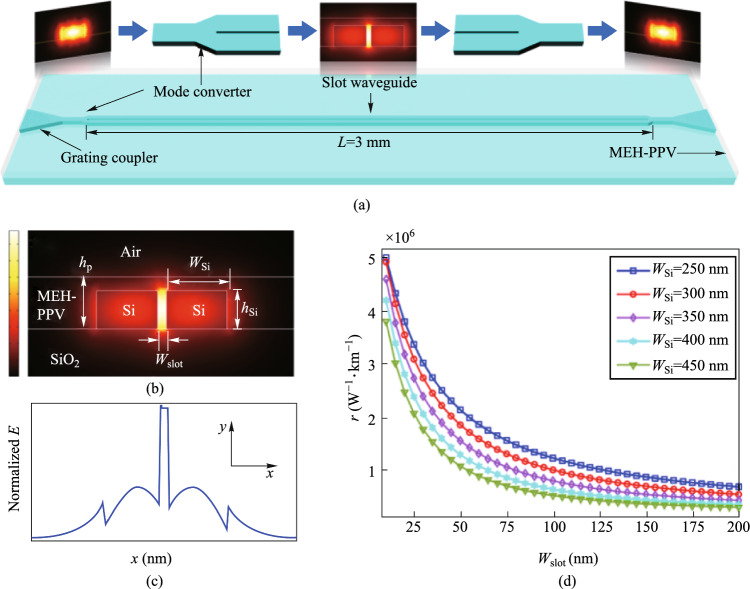
**a** Schematic diagram of the highly nonlinear SOHSW. **b** Normalized 2D electric field distribution and **c** corresponding normalized magnitude of the SOHSW. **d** Evolution of the nonlinear coefficient γ with various slot widths for different waveguide widths

The proposed silicon-organic hybrid slot waveguide (SOHSW) based on a 220 nm SOI wafer is illustrated in Fig. 19a. The slot waveguide is a classic light-field enhancement structure, in which light confinement is achieved by introducing a large discontinuity in the electric field at high-index-contrast interfaces. This confinement results in a strong enhancement of the field in the slot area. The mode profile of the SOHSW made with a 300 nm thick nonlinear polymer layer (*h*_*p*_) is shown in Fig. 19b. Figure 19c shows the corresponding normalized magnitude of electrical field. The light is confined in the nano-scale nonlinear organic slot, which minimize TPA and associated FCA within the silicon due to the relatively weak power density. The MEH-PPV is used to fill the slot with *n*_2_ of 4.5 × 10^−17^ m^2^/W. The evolution of the estimated γ with varying slot widths (*w*_slot_) is calculated for waveguide widths (*w*_si_) ranging from 250 to 450 nm, as depicted in Fig. 19d.

**Fig. 20:**
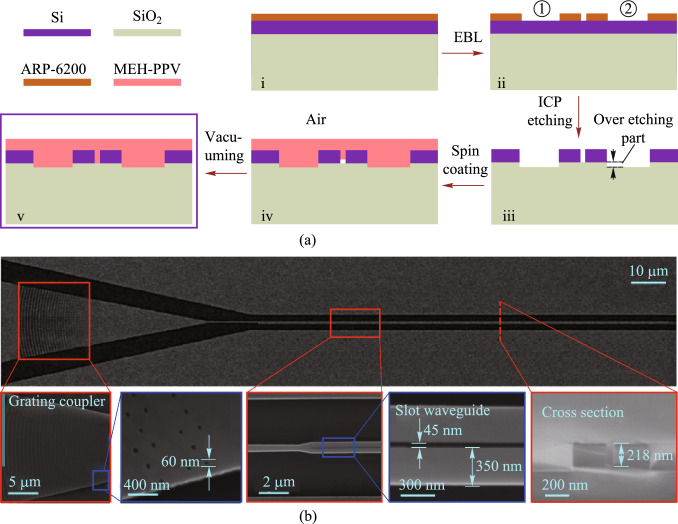
**a** Illustration of the device fabrication steps of the presented SOHSW. **b** Scanning electron microscopy images of the fabricated device

The device is fabricated on standard SOI wafer with a silicon thickness of 220 nm and a buried-oxide thickness of 2 μm. Figure 20a illustrates the fabrication steps used to create the SOHSW. The device pattern is transferred to the silicon layer using EBL and ICP etching. The MEH-PPV cladding layer is prepared by dissolving 20 mg of MEH-PPV powder (Shanghai Aladdin Biochemical Technology Co., Ltd., average molecular weight 70,000–100,000) in 1.5 mL of toluene. The solution is then magnetically stirred at 70 °C for 24 h to ensure complete dissolution. The nonlinear characteristics of the MEH-PPV film are measured using the Z-scan method [50]. Subsequently, the dissolved MEH-PPV is spin coated on the chip at 2000 revolutions per second (RPS) to create a 250 nm film (iv). Any air remaining in the ultra-narrow slot is then removed under vacuum to ensure that the organic material fully fills the slot (v). Scanning electron microscopy (SEM) images of the SOHSW are shown in Fig. 20b. Photonic crystal apodized grating couplers with an initial aperture of 60 nm are used for input and output coupling. The images show that the fabricated SOHSW matches the design, and the measured *w*_slot_, *w*_si_ and slot depth are 45 nm, 350 nm and 218 nm, respectively.

**Fig. 21:**
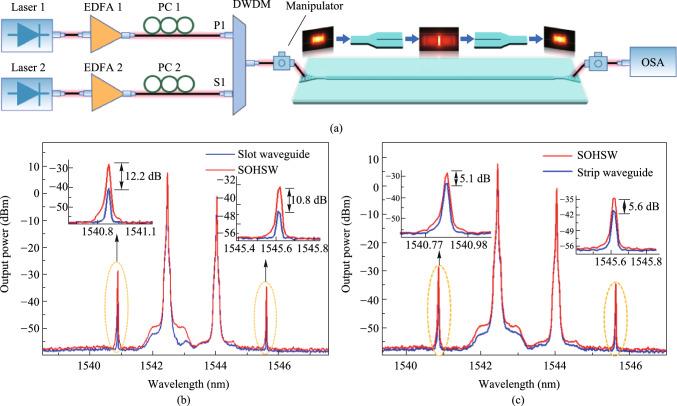
**a** Experimental setup used to measure the nonlinear coefficients. **b** FWM spectra of the SOHSW (red curve) and bare silicon slot waveguide (blue curve). **c** FWM spectra of the SOHSW (red curve) and strip waveguide (220 nm × 450 nm, blue curve)

Figure 21a shows the nonlinear performance characterization of the SOHSW experimental setup. Two CW beams with respective wavelengths of 1542.5 nm (P1) and 1544.1 nm (S1) are amplified, multiplexed and then coupled into the input grating coupler. The average powers of P1 and S1 measured at the output of the dense wavelength division multiplexer (DWDM) are 24 dBm and 19 dBm, respectively. Taking the coupling loss of 5 dB/facet into account, the average powers of the two pumps involved in the SOHSW are 19 dBm and 14 dBm, respectively. Figure 21b displays the FWM spectra measured for the SOHSW (red line) and the bare silicon slot waveguide without the MEH-PPV film (blue line). Both nanostructures have lengths of 3 mm. As illustrated in Fig. 21b, the conversion efficiency using the SOHSW is measured as − 27.5 dB, providing a 12.2 dB enhancement compared to that using the bare slot waveguide as shown in the magnified window. Apart from the slot waveguide, a comparison of the FWM performance of the SOHSW and strip waveguide is also demonstrated as shown in Fig. 21c. The slot in the referenced strip waveguide is 220 nm high and 450 nm wide. In this case, the FWM conversion efficiencies of both idlers achieved with the SOHSW are more than 5 dB higher than those based on the strip waveguide. From the results, the nonlinear coefficient of the fabricated SOHSW is calculated to be as high as 1.43 × 10^6^ W^−1^ km^−1^, showing the promising prospect for using in ultrafast and large-capacity on-chip signal processing in future optical networks.

### Conclusion

Optical logic operations play an important role in the field of high-speed optical computing. We outline the current status and challenges in the development of three research directions: electro-optic oriented logic, all-optical logic and linear interference logic. Among them, all-optical logic has the advantage of high-speed and parallel computing in processing digital signals. By enhancing the nonlinear effect on the integrated devices, it is expected to realize high-speed computing with low power consumption and large capacity. Single-channel 100 Gb/s monolithic integration PLA chip lays the foundation for future high-speed optical signal processing and complex optical computation. Multi-value logic operation devices at 100 Gbit/s shows the potential in building an all-optical logic integrated computing scheme with high-flexibility and high-dimensional. The silicon-organic hybrid slot waveguide is experimentally demonstrated to enhanced optical nonlinearity, showing promising prospects for use in ultrafast and large-capacity on-chip signal processing in future optical networks.

## Multi-dimensional all-optical regeneration photonic chip

*Xinjie Han, Zihao Yang, Hanghang Li, Yong Geng, Jing Xu, Heng Zhou, Kun Qiu*.

### Current status

The amplifier noise and fiber nonlinearities are the main challenges for high-capacity and long-haul fiber optical communication systems [124]. Currently, compensations for these signal impairments are mainly based on digital signal processing (DSP) in the electrical domain, which is still hampered by a few challenges for the next-generation optical transmission networks. First, as the optical fiber communication system evolves to higher data transmission capacity, DSP-based signal compensation might be bottlenecked by the processing speed of DSP modules. Moreover, the complexity, cost, and energy consumption of the involved electrical chips would become unacceptable [125]. Second, to mitigate the Kerr effect induced nonlinear channel impairments, the DSP-based compensation (e.g., digital backpropagation, and deep learning) usually requires unacceptably high computational resources [126]. Third, since DSP-based signal impairment compensation is commonly conducted at the data destination (simultaneously with coherent data receiving), it can hardly be configured as an in-line module (such as a fiber amplifier) that can be placed at intermediated nodes of the transmission link to achieve the optimal effect [127].

On the other hand, phase-sensitive amplification (PSA) can provide low noise optical gain with 6 dB less noise than conventional optical amplifiers [128, 129], such as erbium-doped fiber amplifiers (EDFA) and semiconductor optical amplifiers (SOA). Moreover, PSA is also capable of all-optical mitigation of Kerr-induced nonlinear distortions in the optical domain with a moderate energy consumption [3, 130]. Therefore, PSA-based optical amplification with low-noise gain and all-optical nonlinear compensation capacities is an ideal platform to improve the transmission performance of optical fiber communication.

Plenty of nonlinear material platforms have been applied to realize PSA. Depending on different nonlinear optics platforms, PSA can be realized in *χ*^(2)^ (second-order) nonlinear materials through phase sensitive three-wave mixing (PS-TWM), or in *χ*^(3)^ (third-order) nonlinear materials through phase sensitive four-wave mixing (PS-FWM). In *χ*^(2)^ nonlinear materials [e.g., periodically poled lithium niobate (PPLN)], one pump photon is annihilated while one signal photon and one idler photon are created [131, 132]. In such a PS-TWM nonlinear process, the frequencies of these optical waves satisfy the following relation: ${\omega }_{pump}={\omega }_{signal}+{\omega }_{idler}$. To achieve a stable PSA process, the pump and signal-idler pair should have excellent mutual coherence and should be injected into *χ*^(2)^ nonlinear materials simultaneously. As regards to *χ*^(3)^ (third-order) nonlinear materials, the PS-FWM process involves four optical waves that typically contain two pumps, one signal, and one idler optical wave. To achieve PS-FWM based low noise amplification, two pump photons are annihilated while one signal and one idler photon are created whose frequencies satisfy ${\omega }_{pump1}+{\omega }_{pump2}={\omega }_{signal}+{\omega }_{idler}$. Similarly with PS-TWM, in the PS-FWM process, the two pump waves and signal-idler pair waves should also have excellent mutual coherence to guarantee a stable PS-FWM process [133, 134]. In the following remainder of this section, we focus on FWM-based PSA in third-order nonlinear materials since FWM-based PSA schemes are flexible and can be realized with diversified pump-signal-idler configurations.

In terms of the pump/signal/idler waves configuration, the PS-FWM schemes can be classified into one-mode, two-mode, and four-mode interactions and all these schemes have been experimentally demonstrated so far. Up to now, PS-FWM has been demonstrated in various nonlinear platforms, including semiconductor optical amplifier (SOA) [135], silicon waveguide [136], high nonlinear fiber (HNLF) [8], low-loss silicon nitride waveguide [43], and so on. Regarding applications of PSA, one important PS-FWM based application is to realize low noise optical amplification in optical fiber communication systems. Compared with conventional optical amplifiers, such as EDFA, SOA, Raman amplifier, and phase insensitive optical parametric amplification (PI-OPA), PSA can achieve 6 dB noise figure (NF) improvement [128, 129]. Moreover, low noise PSA applied to the WDM system is also demonstrated [137]. These mean that the PSA has the best potential NF performance compared with any type of optical amplifier ever reported. It is noteworthy that, to ensure that FWM-based optical parametric amplification is working at phase sensitive mode, not only the pump and signal waves are present at the input, but also an idler wave [129]. In 2011, Tong et al. demonstrated experimentally an optical-fiber-based PSA link that has a record-low 1.1 dB noise figure and can be extended to work with multiple wavelength channels for the first time [128]. After that, in 2018, Olsson et al. demonstrated a multi-channel-compatible and modulation-format independent long-haul transmission link with in-line phase-sensitive amplifiers [137]. These previous researches indicate that PSAs can potentially improve the transmission performance of fiber transmission systems.

In addition to the attractive capability of noiseless amplification, the capacity of all optical nonlinear mitigation, which is based on the concept of optical phase multilevel quantization [9, 138] (i.e., all-optical phase regeneration), is also one of the most important advantages of PSA. In the third-order nonlinear material platforms, for the *M*-level phase-encoded optical signals, the phase quantization can be achieved by coherently adding the (*M − *1)th phase harmonic of signal to signal itself with a magnitude ratio of *M − *1:1 through Kerr-based nonlinear FWM process [9]. In 2010, based on the on-mode scheme, Richardson et al. reported the first practical all-optical regenerator that is capable of removing both phase and amplitude noise from binary phase encoded optical signals [8]. After that, Richardson et al. further demonstrated a PSA-based all-optical signal processing architecture that enables multilevel all-optical quantization of phase-encoded optical signals. They demonstrate phase quantization up to six levels in Ref. [9]. These two important types of researches are key progresses to optical fiber communication and show a new application scenario of the nonlinear PS-FWM process.

### Challenges and solutions

PSAs exhibit gain that varies with the phase of the input signal relative to a local optical reference [137]. The theory underlying PSAs dates back over five decades ago with early demonstrations employed second-order nonlinearity in bulk crystals. Later, the use of third-order nonlinearity in optical fibers significantly advanced their practicality due to enhanced robustness, power efficiency, and system integration [139]. While PSAs were once considered impractical due to stringent phase-locking requirements, recent advances—particularly in suppressing stimulated Brillouin scattering [140]—have paved the way for practical phase-sensitive fiber optical parametric amplifiers [141]. These developments offer low-noise, wideband amplification and hold promise for deployment in modern optical networks. Although the nonlinear PSA has many excellent advantages, the PSA-based applications are still immature, and many practical aspects need to be addressed. Some of the major challenges are discussed here.

First, phase manipulation is challenging. To achieve stable PSA operation, the pump, signal, and idler waves should have excellent mutual coherence, meanwhile, the relative phase among these three optical waves should remain constant [9, 128, 139]. In practical situations, the slow relative phase drifts among the pump, signal, and idler waves in a fiber link induced by temperature variations and acoustic vibrations are serious and inevitable. Such random phase drifts will further lead to optical gain fluctuations and need to be addressed. The solution now used to suppress the relative phases is monitoring the power of the regenerated channel as an error signal and controlling the relative length of the fiber link by piezoelectric transducer [128]. However, this method is complex and difficult and needs further investigation.

Second, the generation of coherent multi-wavelength pump waves is challenging. For the PS-FWM based all-optical phase regenerator, how to construct coherent multi-wavelength pump lasers is the main challenge. In nonlinear PS-FWM dynamic processes, the multi-wavelength pump lasers should be frequency separated in a particular frequency spacing, while simultaneously maintaining phase coherence. In most previous research, an extra copier stage was used to produce the multi-wavelength continuous wave (CW) pump lasers. The copier stage is composed mainly of a nonlinear component (e.g., HNLF, SOA) adopted to generate high order FWM side band and a semiconductor laser used to eliminate modulated information from the FWM side band [9]. Obviously, the extra copier stage makes the system complex for practical implement. Furthermore, the copier stage scheme cannot support phase regeneration of high-order modulation formats (e.g., 6PSK, 8PSK) because of the limited nonlinear FWM conversion efficiency. A promising solution is using an optical frequency comb instead of the copier stage which will be carefully discussed later.

Third, the enhancement of nonlinear effects is challenging. PS-FWM based low noise optical amplification and phase regeneration are based on third-order nonlinear effect in* χ*^(3)^ nonlinear materials. Therefore, the nonlinear effect performance of the nonlinear materials is crucial to optical amplification and phase regeneration. HNLF is the most widely used nonlinear platform for PSA, but the inherent SBS effect in fiber hampers the maximum input optical pump power, which in turn limits the FWM conversion efficiency [140]. The common solution is to introduce phase dithering to pump lasers [141]. However, this technique introduces additional phase noise to the pump which further degrades signal performance. Introducing novel nonlinear platforms is a promising solution, such as silicon waveguide [142], distributed straining fiber [9], thin film lithium niobate (TFLN) waveguide [143], silicon nitride waveguide [43]. Novel photonic integrated silicon nitride waveguide adopted to achieve optical parametric amplification has been experimentally demonstrated lately [43]. Silicon nitride waveguides exhibit ultra-low loss, enabling significant accumulation of optical nonlinearity over long-distance on-chip propagation. This distinctive behavior makes them well-suited for integrated PSA applications. In this work, a peak gain of 12 dB on a silicon nitride chip has been achieved and provides a novel photonic integrated solution to PSA-based applications.

### Progress

We explore the third-order nonlinearity for both phase as well as amplitude regenerations based on innovated photonic devices, as described in the following three parts.

#### Multi-channel all-optical phase regeneration based on Kerr combs and silicon waveguides

We demonstrate all-optical phase regeneration for WDM channels with a novel photonic integrated architecture, which is composed of a low-loss silicon waveguide and a silicon nitride micro-ring. The silicon waveguide performs as the signal regenerator with reversed biased PIN junction used as a nonlinear platform to enhance PS-FWM nonlinear dynamic, while the silicon nitride micro-ring is used to generate dissipative Kerr soliton frequency comb to provide coherent multi-wavelength pump lasers. In particular, multi-channel all-optical phase regeneration is experimentally demonstrated for 40 Gbit/s QPSK data, achieving the best SNR-improving of more than 6 dB. Our study showcases a promising avenue to enable the practical implementation of all-optical phase regeneration in realistic long-distance fiber transmission networks.

1. Integrated silicon regenerator

**Fig. 22:**
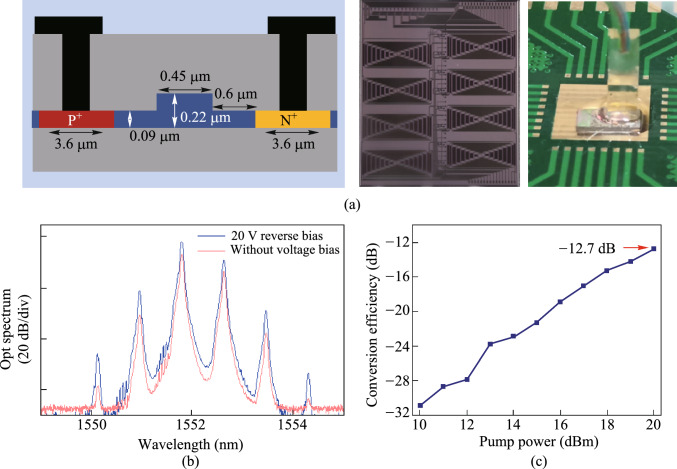
**a** Cross section and picture of silicon waveguide used in our experiment. **b** Measured optical spectra for the phase insensitive nonlinear FWM with 20 V reverse bias (blue line) and without 20 V reverse bias (red line), at pump power of 20 dBm. **c** Phase insensitive nonlinear FWM conversion efficiency for different input pump power, with 20 V reverse bias implemented to the waveguide’s p–i–n junction

The nonlinear all-optical regenerator used in our experiment is an SOI photonic chip containing multiple 4 cm-long silicon waveguides (Fig. 22). The dimensions of the ridge waveguide are shown in Fig. 22a, whose width, height and slab height are 450 nm, 220 nm, and 90 nm, respectively. The measured linear loss of the silicon waveguide is about 1.5 dB/cm. For easy and stable external driving, the silicon chip is packaged by a multi-channel fiber array with a typical end-to-end loss of about 9 dB. To remove free carriers from the silicon waveguide, a lateral p–i–n junction is implanted along the waveguide rib by doping with boron and phosphorus ions respectively. Reverse voltage bias is implemented to the waveguide’s p–i–n junction to shorten the free carrier lifetime and avoid severe free-carrier absorption to enhance nonlinear conversion efficiency. In particular, at 20 V reverse bias, phase-insensitive nonlinear FWM conversion efficiency can reach as high as − 13 dB in our silicon waveguides, at reasonably high pump powers (< 20 dBm), as shown in Fig. 22b. Compared with 0 V reverse bias, the conversion efficiency increases significantly. Meanwhile, the nonlinear FWM conversion efficiency for different input pump power with constant 20 V reverse bias is measured and shown in Fig. 22c.

(2) Kerr comb based integrated multi-wavelength coherent pump lasers

**Fig. 23:**
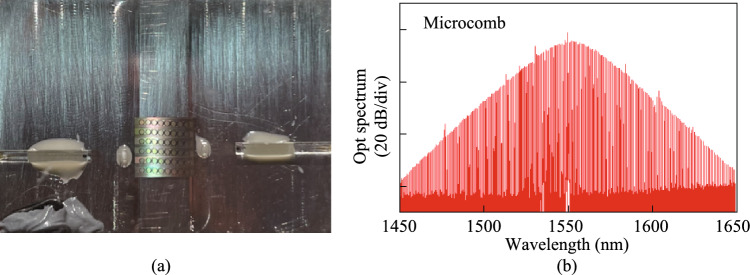
**a** Picture of packaged Si_3_N_4_ chips used in our experiment. **b** Measured optical spectrum of generated Kerr soliton comb

Up to now, all-optical phase regeneration has not been seen in realistic fiber transmission systems, mainly due to the nonlinear PS-FWM dynamic requires multiple light waves, all of which need to be separated at particular frequency spacing and highly phase coherence. As a consequence of these stringent requirements, current all-optical phase regenerators usually consist of highly complex apparatus that are bulky, expensive, power hungry and hard to operate. In this research, we reported a novel scheme of multi-wavelength coherent pump lasers generation based on an integrated Kerr comb to address the above challenges. The Kerr soliton frequency comb is generated in a high-*Q* silicon nitride (Si_3_N_4_) micro-ring (see Fig. 23a). The cross section of the micro-ring is 1650 nm × 800 nm and the free spectral range (FSR) is about 100 GHz. The Si_3_N_4_ chip is packaged with polarization-maintaining I/O fibers to ensure the long-term and stable operation of the Kerr soliton comb. The optical spectrum of the generated Kerr soliton comb is illustrated in Fig. 23b, with a smooth spectral envelope. For all-optical phase regeneration of QPSK, three comb lines are filtered out from the Kerr soliton comb and used as a signal carrier and two pump waves, respectively. Thanks to the excellent mutual coherence among all comb lines, the relative phases among the selected three comb lines are stable and can be manipulated flexibly to meet the high gain PSA conditions.

(3) Experimental setup and results

**Fig. 24:**
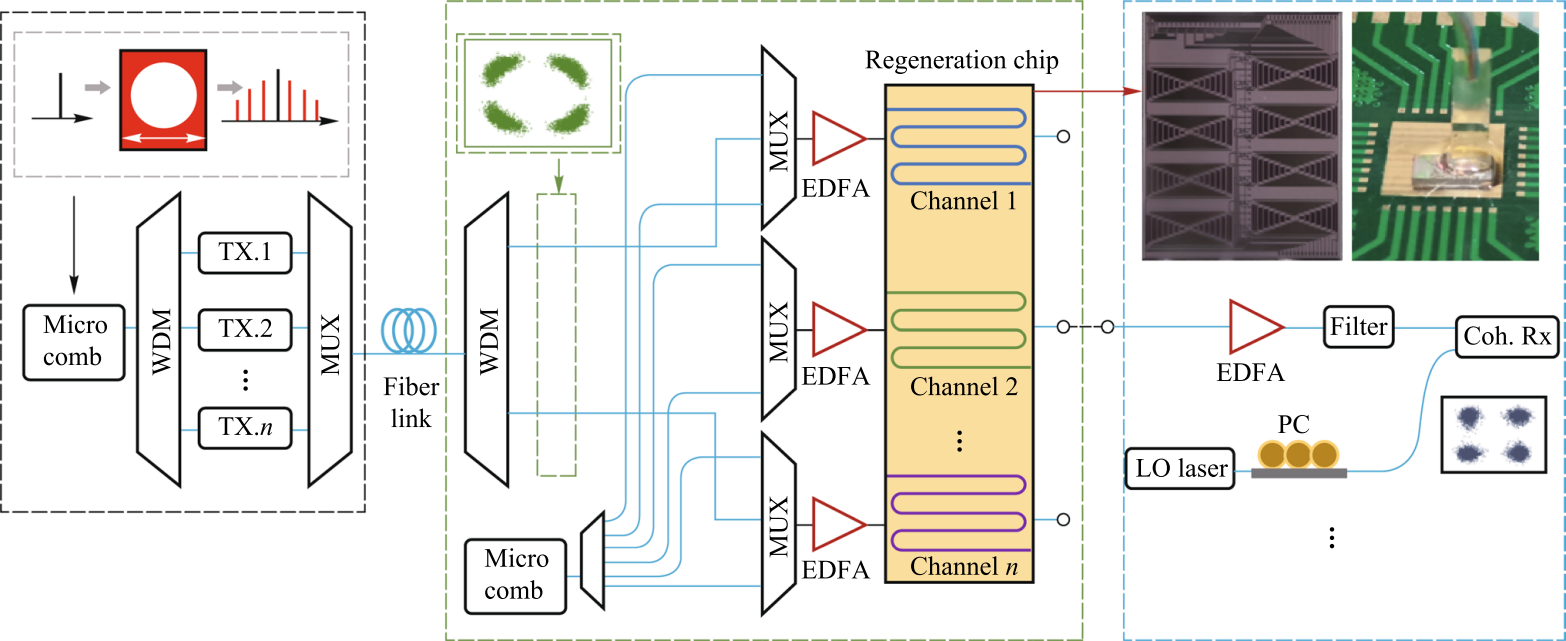
Experiment setup for all-optical phase regeneration

**Fig. 25:**
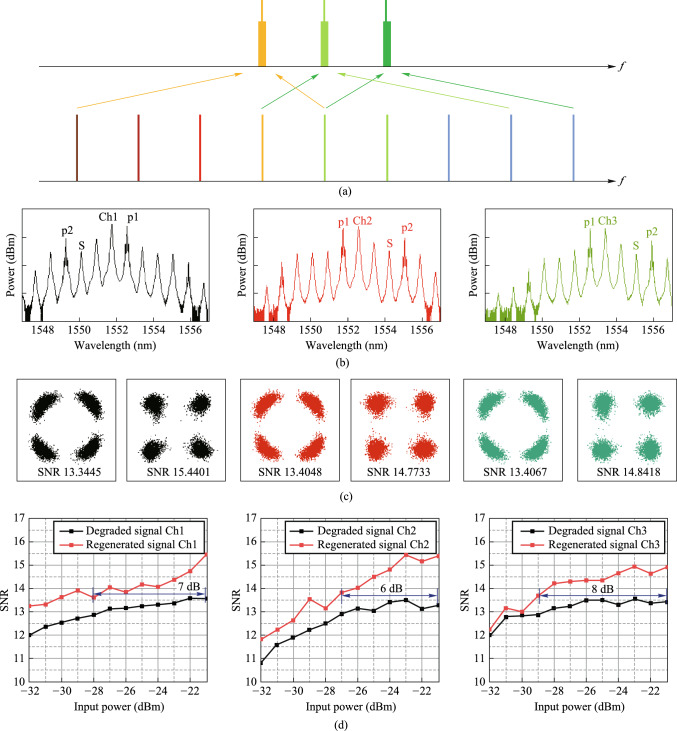
**a** Specific pump lasers and signal channel configuration for three channel QPSK all-optical phase regeneration in our experiment. **b** Measured optical spectra of the phase sensitive FWM processes for all three data channels. P1 and P2 are the double pumps required in the phase sensitive FWM process, Ch1, Ch2, and Ch3 are the data channels, and the beam of the light marked with s is the regenerated signal. **c** Constellation diagram and signal-to-noise ratio of original distorted signals (left) and regenerated signals (right) for all three data channels. **d** Measured SNR of original distorted signals (black) and regenerated signals (red) for different input coherent receiver power for all three data channels

Figure 24 illustrates the experiment setup of our novel all-optical phase regenerator. At the transmitter, a high *Q* silicon nitride micro-ring is used to generate a Kerr soliton frequency comb. Three comb lines are selected from the Kerr soliton comb by a WDM multiplexer and injected into an IQ modulator to load 40 Gbit/s QPSK data. Phase noise is artificially imposed onto the signal (as exemplified in the inset of Fig. 24). The three channel QPSK signals are then combined using another WDM multiplexer, sent through to a spool of fiber that mimics the transmission link, and reach the all-optical phase regenerator, as shown in the central block of Fig. 24. At the regenerator, the three channel QPSK data are demultiplexed and sent into a silicon photonic chip that contains multiple nonlinear waveguides for all-optical phase regeneration. 20 V reverse bias is applied to the p–i–n junction of each waveguide to remove the free carriers generated in the PS-FWM process. At the regenerator stage, to achieve four-level phase squeezing, the signal should be coherently added to its conjugated 3rd harmonic with an amplitude ratio of 3:1. In our experiment, an idler-free scheme is adopted to meet the above requirements [144]. The Kerr micro-comb lines generated at the regeneration stage can be selected to serve as the CW pump lasers for PS-FWM. Figure 25a shows the specific pump laser configuration for three channel QPSK all-optical phase regeneration in our experiment. It is also seen that, as the Kerr microcomb usually has a broadband spectrum, one Kerr microcomb at the regeneration stage can potentially support phase regeneration of any WDM channels across the whole C- and even the L-band (amplifiers are needed to guarantee enough power budget though).

The selected comb lines as CW pump lasers are then sent to the silicon waveguide together with the corresponding QPSK signal, and the PS-FWM processes required by all-optical phase regeneration are achieved for all three data channels. The PS-FWM spectra are shown in Fig. 25b. The idler waves corresponding to the phase regenerated signals are then filtered out and feed into a coherent data receiver, wherein the constellation maps and signal-to-noise ratio (SNR) of the regenerated signals are retrieved and compared with the original distorted signals, the results are summarized in Fig. 25c. It is observed that, after all-optical phase regeneration, all three QPSK signals exhibit substantially reshaped constellation maps (the phase variances before and after regeneration are also listed for each channel). We also measured the data SNR as the function of input power to the coherent receiver, as shown in Fig. 25d. For data channel Ch1(Ch2/Ch3), to achieve an SNR of 13.6 dB, the input power injected into the coherent receiver for degraded and regenerated signal are − 21 dBm (− 21 dBm/− 21 dBm) and − 28 dBm (− 27 dBm/− 29 dBm) respectively. So, the receiver sensitivity is improved by − 21 − (− 28) = 7 dB (6 dB/8 dB). Hence, it is seen that > 6 dB improvement in receiver sensitivity is achieved for each of the three QPSK signals.

In Table 7, we present a comprehensive comparison between our work and alternative nonlinear regeneration schemes. By incorporating silicon-based waveguides with solitary solitons, multi-wavelength, all-optical amplitude and phase regeneration capabilities for PAM4 and QPSK signals alike have been successfully achieved.

**Table 7:** Performance of nonlinear regeneration materials

Refs.	Materials	Regeneration scheme	On-chip integration	Reconfigurable	Additional equipment	Regenerative phase number/amplitude number
[137]	HNLF	FWM + injection locked + PSA	No	No	2 nonlinear materials and slave lasers	4/0
[145]	HNLF	Electro-optic modulation + PSA	No	Yes	Photoelectric modulator	2/0
[136]	HNLF + SOI photonic chip	FWM + injection locked + PSA	No	No	2 nonlinear materials and slave lasers	4/0
[146]	HNLF	Optical time lense + BPSK changed to OOK + PSA	No	No	4 nonlinear materials	2/0
[147]	Silicon waveguide	FWM	Yes	No	No	0/4
[148]	Silicon waveguide	FWM	Yes	No	No	0/2
This work	Silicon waveguide	Soliton + PSA	Yes	Yes	No	4/4

#### Increase the capacity of all-optical amplitude regeneration based on on-chip mode division multiplexing technique

Mode-division multiplexing (MDM) has been proposed as an effective way to enhance information capacity of optical communication networks. Similarly, MDM has the potential to increase the processing capacity of AOSP chips. The practical application of multimode all-optical regenerators is often challenged by the difficulty of regenerating signals in higher-order modes. In this project, we experimentally demonstrate the all-optical regeneration of a 40 Gb/s non-return-to-zero on–off keying (NRZ-OOK) signal in both TE0 and TE1 modes through four-wave mixing (FWM) in a low-loss silicon-based nanowaveguide [10]. The extinction ratio (ER) enhancement achieved for both modes is approximately 6 dB after regeneration, indicating the potential of enlarging the all optical signal processing capacity of silicon integrated circuits based on MDM techniques.

**Fig. 26:**
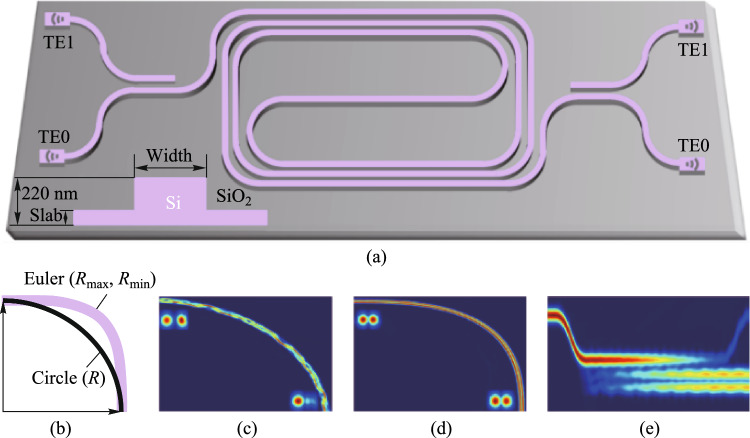
**a** Schematic diagram of the multimode waveguide structure. **b** Diagram of waveguide bending with constant radius of curvature and evolution of the radius of curvature, i.e., Euler bending. **c** and **d** Simulated comparison results of TE1 mode transmission in the circular waveguide and Euler curved waveguide. **e** Simulation result of TE1 mode multiplexing

As shown in Fig. 26a, the core components of the low-loss multimode regenerator include low-loss nonlinear multimode waveguides, mode multiplexers and demultiplexers. The FWM process takes place in the nonlinear waveguide, while the mode multiplexer facilitates the conversion between optical modes of different orders, thereby enabling multimode signal regeneration.

The design of the low-loss nonlinear waveguide, necessitates balancing between minimizing loss and maximizing the nonlinear coefficient. Moreover, proper dispersion management is crucial to ensure adequate bandwidth. Consequently, we have designed a ridge waveguide with a 2000 nm width, a 70 nm height, and a 9.5 cm length. To prevent crosstalk in the low-loss waveguides, careful planning of the bend geometry is required. Figure 26b shows a comparison of the two curves. The simulation results illustrated in Fig. 26c and d, conducted using Lumerical FDTD software, demonstrate the superiority of the Euler bending waveguide over a traditional circular waveguide in terms of minimizing transmission loss and mode crosstalk for higher-order modes. The mode multiplexer, a key component of the multimode system, is shown in Fig. 26e, designed based on a directional coupler to transform optical fields from TE0 to TE1 mode. The crosstalk between two modes is measured to be less than − 20 dB. The FWM conversion efficiencies for TE0 and TE1 modes at different pump powers, are measured to reach − 9.6 dB and − 13.0 dB, respectively. The FWM conversion efficiency, in both modes, fits well with theoretical predictions at lower power levels, whereas at high optical powers onboard, the efficiency tends to saturate due to two-photon absorption (TPA) and free carrier absorption (FCA) effects. The 3 dB conversion bandwidth for the TE0 and TE1 modes are measured to be 10.6 nm and 12.0 nm, respectively, consistent with numerical simulations.

For experimental verification, a continuous light with the wavelength setting to 1549.2 nm is used as the pump light, while another laser with the wavelength at 1550.8 nm is modulated by the Mach–Zehnder modulator (MZM) to generate 40 Gb/s NRZ-OOK signal. Two beams of light are combined through the coupler and incident on the silicon-based waveguide. The light is pre-amplified by an optical amplifier and filtered by tunable bandpass filter to obtain idler frequency light, which is the regenerated data signal, and its wavelength is 1552.4 nm. Finally, eye diagram observation and signal quality analysis are performed by an oscilloscope (OSC), and bit error rate (BER) measurement is performed by bit error rate tester (BERT). The measured FWM spectra show a conversion efficiency of − 16.1 dB for TE0 mode and − 20.7 dB for TE1 mode, respectively.

**Fig. 27:**
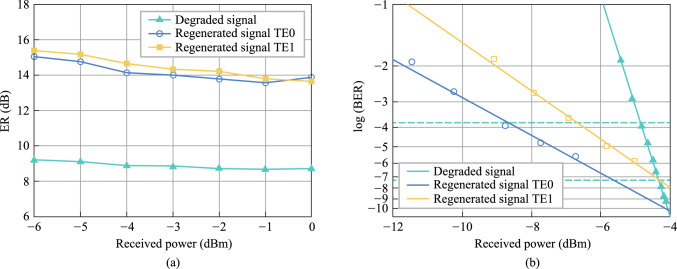
**a** ER curves of degraded signals, TE0 mode and TE1 mode regeneration signals at received power from − 6 to 0 dBm, which are given by green, blue and yellow curves, respectively. **b** BER curves of degraded signal, TE0 mode and TE1 mode regenerated signal under different received power

To evaluate the performance of the regenerator under different optical power conditions, the OSC receives signals of different powers by adjusting the power before the entering the OSC. The ER curves under different received optical power are shown in Fig. 27a. Compared with the degraded signal, the ER of the regenerated signal in TE0 and TE1 mode has a 5–6 dB improvement. The ER increase of TE0 mode regenerated signal can reach 5.85 dB. The ER increase of TE1 mode regenerated signal can reach 6.21 dB. The BER of the degraded signal and the regenerated signal is measured as shown in Fig. 27b. As can be seen from the blue dashed line, BER below 10^−7^ is achieved when the received power is − 4.3 dBm. When the optical power of the degraded signal is below − 4.3 dBm, TE0 and TE1 mode regenerated signals obtain a notable regeneration effect. When the target BER is the hard decision forward error correction (HD-FEC) threshold of 3.8 × 10^−3^, the receiver sensitivity is improved by 3.85 dB and 1.85 dB for TE0 and TE1 mode, respectively.

#### High-speed ultra-efficient all-optical wavelength conversion based on high-Q microresonators using parity-time symmetry

**Fig. 28:**
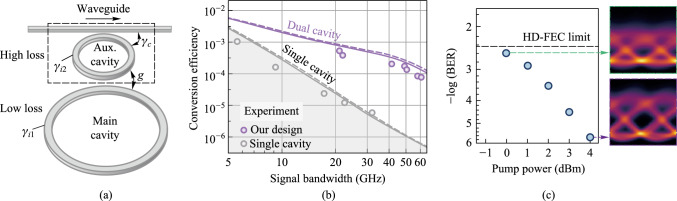
**a** Schematic diagram of dual coupled microresonators using parity-time symmetry. **b** Measured conversion efficiency for single cavity as well as dual cavity. **c** BER measurements versus different pump power of On–Off Keying signals at 38 GBaud

It is known that microresonators with high quality factor (*Q*) can be used to enhance intracavity photon density, opening new avenues for a wide range of nonlinear applications such as the generation of Kerr soliton microcombs. However, the enhanced nonlinear light-matter interaction comes at the cost of the response speed due to Fourier reciprocity. Since the linewidth is inversely related to the *Q*, there is a fundamental trade-off between the high-*Q* cavity enhanced nonlinearity and the maximum signal bandwidth $B$ (proportional to the data rate) supported by a resonator. Various schemes have been investigated to overcome this bandwidth-efficiency limit [149, 150]. However, challenges on structural complexity, footprint, limited performance still exist, or quantum applications other than AOSP are discussed. Strong nonlinear effects can also be achieved by increasing the interaction lengths of the nonlinear medium [43]. However, it is limited by large device footprint, reduced phase-matching bandwidth, and demanding fabrication tolerance. Note that four wave mixing is a third-order nonlinearity that involves interaction of four waves. For most AOSP applications, pump wave is a continuous wave light, which is narrow linewidth. Therefore, it is possible to engineer the spectrum of microresonators so that different resonances may have different linewidth. To balance the requirement for high-speed and high efficiency, the linewidth of the pump resonance can be selectively reduced to achieve large field enhancement, while the linewidth of the signal and idler resonances is kept large to facilitate high-speed operation. To enable this operation, a dual coupled microresonator system based on parity-time (PT) symmetry has been proposed in this project to overcome the trade-off between speed and efficiency (Fig. 28a) [151]. By setting the ratio of the length of the two microresonators as 1:2, the transmission spectrum can be effectively manipulated. With proposed designed coupling coefficients, the signal and idler resonances are broadband due to the resonances merging effect occurs at the exceptional point, while the pump resonance is narrowband due to the operation in the broken PT-symmetry regime. With this specific design, two-order of magnitude boost of conversion efficiency has been achieved at 40 GHz signal bandwidth. A series of PT structures have been fabricated on high nonlinear integrated platform, i.e., AlGaAs on Insulator. Systematic verification of wavelength conversion operation shows that our scheme is power efficient. By using only 1mW pump power, error-free wavelength conversion at 38GBaud has been achieved at hard-decision forward-error-correction (HD-FEC) limit. The device also features small footprint (< 0.01 mm^2^) as well as broad conversion range (> 170 nm).

### Conclusion

In conclusion, we have demonstrated a new architecture to implement multi-channel and reconfigurable all-optical phase regeneration, by using photonic integrated devices including chip-scale Kerr soliton microcomb and high efficiency nonlinear silicon waveguide. All-optical phase regenerations of three channel 40 Gbit/s QPSK signal have been achieved with a novel system that is much simpler than traditionally schemes (mainly thanks to that the complex copier stage is eliminated). We also demonstrate regenerations of amplitude modulated signal using mode division multiplexing techniques as well as multimode waveguides. A parity-time symmetry scheme has been prosed and verified to overcome the trade-off between nonlinear efficiency and processing speed.

## Multi-channel and multi-functional AOSP chip together with its packaging

*Bei Chen, Huashun Wen, Kunpeng Zhai, Xuhan Guo, Ming Li, Jianguo Liu, Ninghua Zhu*.

### Current status

As the surge of various emerging applications in the fields of telecommunications, quantum information processing, sensing and neuromorphic photonic computing, all-optical signal processing (AOSP) chips with single function are not enough to support the practical requirements. Gradually, the interest in integrated multi-channel and multi-functional AOSP chips with high density is driven, in order to supply flexible, reconfigurable, high-speed, low-power-consuming, compact and easily-fabricated optical devices. In fact, a universal AOSP chip with multiple functions, is similar to an electronic field-programmable gate array (FPGA)-based signal processor.

To date, various optical processing chips with different functions have been reported, where the implemented functions are mainly focused on all-optical routing [151], logical operation [113], signal regeneration [9], wavelength selective switching [76], filtering [152], wavelength conversion [153] and so on. These functions are basic building blocks of a universal AOSP chip for signal generation and fast computing. The programmable reconfiguration capability of AOSP chips can improve the environmental survivability of photonic systems, and enhance the spectrum utilization efficiency. Hence, it has the potential to realize intelligent optical communication network systems.

As demonstrated, the reported multi-channel and multi-functional AOSP chips are built up with different structures, such as Mach–Zehnder interferometers (MZIs) [154, 155], microring resonators (MRRs) [156], micro-disk resonators (MDRs) [157], waveguide Bragg gratings [158] and so on. In 2015, Zhuang et al. demonstrated a FPGA-like photonic signal processor chip by means of a grid of tunable MZIs, which was fabricated in the commercial Si_3_N_4_ foundry. Four different functions including notch filter, Hilbert transformer, bandpass filter and programmable delay lines are integrated in the same chip with the footprint of 3.5 mm × 8.5 mm [154]. After that, in 2016, Liu et al. experimentally demonstrated a fully reconfigurable photonic integrated signal processor based on an InP-InGaAsP material platform. The proposed AOSP was capable of performing reconfigurable signal processing functions including temporal integration, temporal differentiation and Hilbert transformation by controlling the injection currents of the active components [157]. Moreover, in 2017, Pérez et al. demonstrated over 20 different functionalities with a seven hexagonal cell structure, in which the chip footprint is 15 mm × 20 mm [157]. In 2018, Zhang et al. proposed the reconfigurable grating consisting of multiple series-connected uniform Bragg grating sections and a Fabry–Perot (FP) cavity section in the center of the grating, which was fabricated in a CMOS-compatible process using 248-nm deep ultraviolet lithography. The proposed reconfigurable grating can perform temporal differentiation, microwave time delay, and frequency identification [158]. Furthermore, in 2020, Zhang et al. demonstrated a scalable photonic field-programmable disk array (FPDA) signal processor based on ultra-compact microdisk resonators. By field-programming the processor, the proposed FPDA signal processor can be realized to perform multiple specific signal processing functions including filtering, temporal differentiation, time delay, beamforming, and spectral shaping [159]. These schemes fully verify the good performances and various characteristics of the multi-channel and multi-functional AOSP chips. Meanwhile, the proposed AOSP chips can be further applied to different fields including communications, chemical and biomedical sensing, signal processing, multiprocessor networks, and quantum information systems. In addition, advanced optical and electrical packaging technology [160], including ultralow-loss optical coupling, electronic wire bonding, and thermal management, is indispensable to realize integrated optoelectronic systems with high density, high speed, good signal integrity and low crosstalk.

### Challenges and solutions

Nevertheless, the implementation of highly integrated AOSP chips is still facing multiple challenges. One aspect is that the basic optical units with different functions are based on various materials or multiple cross-sections with different footprints, due to that the requirements of different applications and their corresponding performances of the designed devices are quite different. It means that heterogeneous [161] or hybrid [162] integration technology is required for AOSP chips with multiple channels and functions. Meanwhile, spot-size convertors [163] or waveguide transition structures [164] with compact footprint and ultralow loss are necessary for a good match of optical filed distributions. Another aspect is that compact fiber-to-chip light coupling with low loss and large bandwidth [165], single mode fiber (SMF)-to-chip edge coupler in particular, is extensively demanded in integrated photonics. The inescapable challenge of edge coupler is the difficulty and complexity in fabrication and packaging. In further, as the integration scale increases, severe crosstalk [166] among optical, electrical and heat filed may be introduced into the systems, due to the complexity of the coupling mechanism between radio frequency and optical wave.

Specifically, the key challenges of the AOSP chips with different functions are diverse. As for on-chip programmable optical filtering and multidimensional signal regeneration, flexible and precise tuning for optical fields are important aspects, in which e.g., thermo-optic (TO) effect [167], electro-optic (EO) effect [168], acoustic-optic (AO) effect [169], magneto-optic (MO) effect [170] can be adopted for the adjustments. Among them, TO effect is one of the most popular options for the adjustments due to that TO effect is available for almost all materials and easy to be fabricated. In particular, the TO coefficient of silicon materials is $1.8\times {10}^{-4 }\space{\text{K}}^{-1}$ and the corresponding heat conductivity is around 149 W/(m·K) [171, 172]. As a representative scheme, the reported programmable optical filters in Ref. [152] can be tuned thermally by locally controlling the temperature in the phase-shifting region with micro-heaters. Besides, ultra-low loss and the enhancement of nonlinear effect can improve the performances of on-chip signal regeneration and programmable logic computing. According to the reported works, there are two typical methods to reduce the scattering losses in optical waveguides. One is to smoothen the waveguide sidewalls by improving the fabrication processes or introducing some specific processes, such as post-processing [173, 174], anisotropic etching [58], and chemical oxidation [175]. However, these processes are usually incompatible with the multi-project-wafer (MPW) foundry for silicon photonics, and thus increase the complexity for further photonic integration. The other issue is to decrease the interaction between optical fields with rough sidewalls by using a shallowly etched ridge or ultrathin core region [52, 176]. For example, a shallowly etched silicon-based ridge waveguide with a cross section of 0.25 μm × 2 μm and an etching depth of 50 nm was developed, and the propagation loss was reduced to be ~ 0.274 dB/cm [52]. To address different challenges in different AOPS functions, the optimal geometry adopted in filter, logic as well as regeneration operations are different. Therefore, low loss mode converter between each function are required. Moreover, as for multi-channel AOSP chips with large-scale integration, it will all face the problem of the crosstalk among optical, electrical and thermal fields.

To solve the abovementioned problems, we proposed and demonstrated a silicon-based multi-channel and multi-functional AOSP chip, together with advanced optoelectronic packaging technology. Three different functions including reconfigurable filtering, signal regeneration and logical operation were all experimentally implemented.

### Progress

#### Integrated multi-channel AOSP chip with multiple functions

**Fig. 29:**
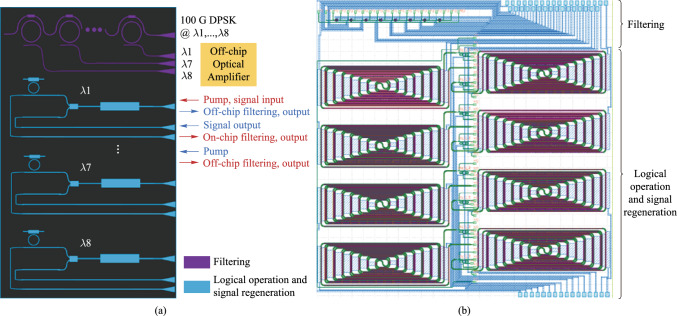
**a** Schematic of the proposed multi-channel AOSP chip with multiple functions. **b** Corresponding layout

In this section, a silicon-based multi-channel AOSP chip with multiple functions was proposed and demonstrated, as shown in Fig. 29. It consisted of eight wavelength channels with total capacity over 800 Gb/s. Three different functions including filtering, logical operation and signal regeneration functions can be realized. The basic filtering unit was mainly based on the Mach–Zehnder interferometer (MZI)-assisted microring resonator (MRR). Meanwhile, the functions of logical operation and signal regeneration were relied on the silicon waveguides with reverse-biased p–i–n junction. As depicted in Fig. 29a, eight different wavelengths modulated by differential phase shift keying (DPSK) method with speed of 100 Gb/s were coupled into the fabricated AOSP chip by the optical grating coupler (GC). By tuning each phase shifter on the filtering units, the corresponding operating center wavelengths were consistent with the input wavelengths. Each filtering unit was realized by the MZI-assisted MRR, i.e., based on the technology described in Sect. 2. Eight modulated signals can be dropped by each filtering unit and coupled to eight output GCs. Subsequently, after off-chip amplification, these eight modulated signals were sent into eight function units for logical operation and signal regeneration. Each function unit mainly consisted of a MRR and a spiral silicon waveguide with p–i–n junction, based on the technology described in Sects. 3 and 4. As already mentioned, additional transition structures were introduced, due to the inevitable mode mismatch caused by different propagation waveguides in multiple functional units. In addition, Fig. 29b demonstrated the corresponding chip layout for eight-channel AOSP chip. Fiber arrays with an equal space of 127 $\upmu$m were used for multi-channel coupling of input and output signals. A total of 136 optical/electrical devices were monolithically integrated on the fabricated AOSP chip.

The proposed eight-channel AOSP chip was fabricated on a standard 8-inch process platform of Chongqing United Micro-Electronics Center (CUMEC), China.

**Fig. 30:**
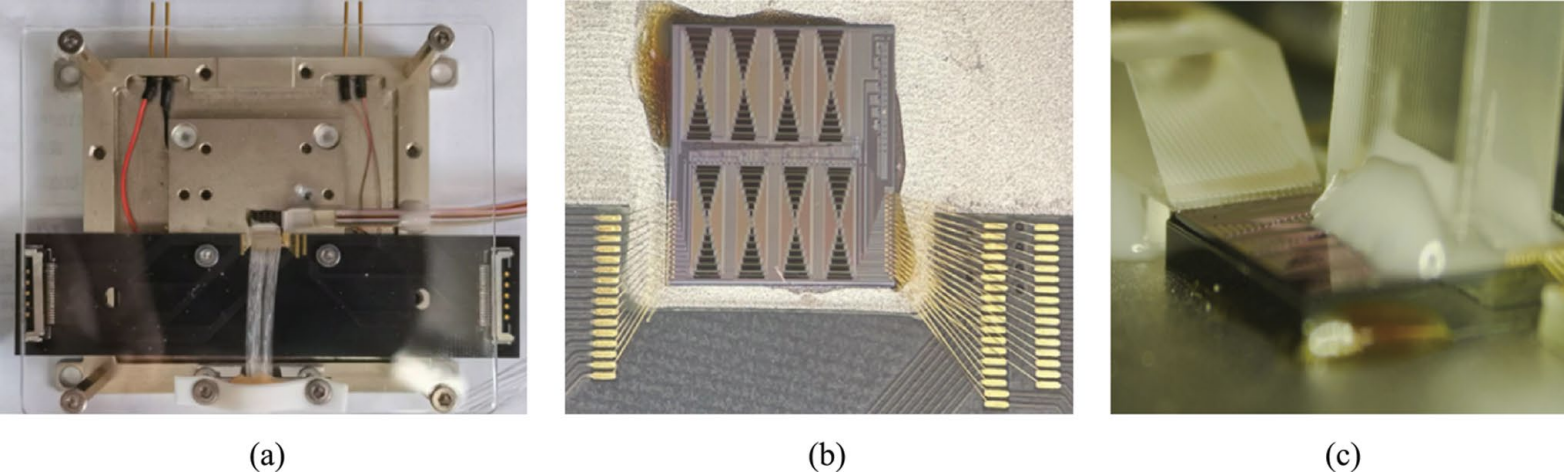
**a** Image of packaged eight-channel AOSP chip. **b** Electrical and **c** optical packages

**Fig. 31:**
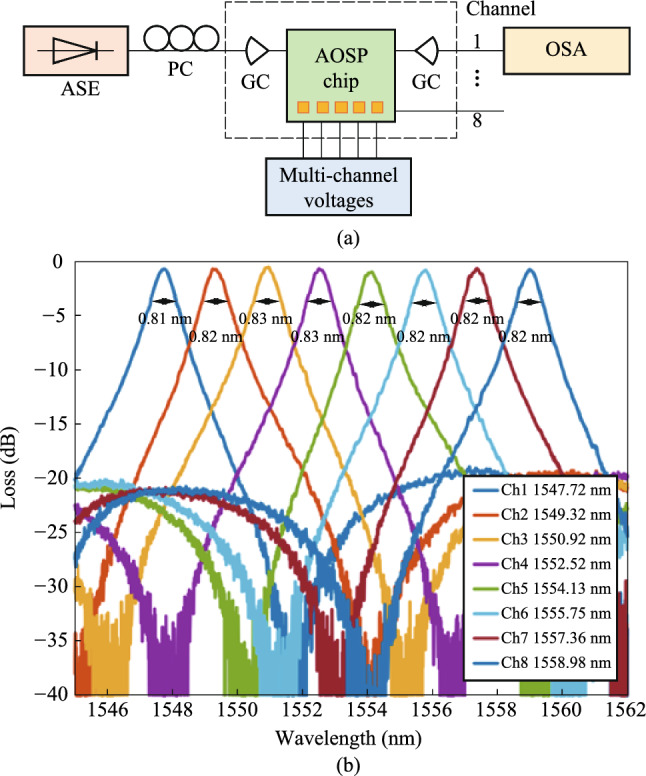
**a** Experimental setup for “filtering” function of the proposed AOSP chip. **b** Transmission spectra of eight channels

To fully demonstrate the performances of the packaged eight-channel AOSP chip (shown in Fig. 30), three different functions including filtering, logical operation and signal regeneration were tested, respectively. The filtering function means that signals with different wavelengths can be distinguished and dropped to the specific channel. In our eight-channel AOSP chip, this function is realized by eight MZI-assisted MRRs, which are tuned to be resonant under eight different wavelengths. Meanwhile, due to their add/drop structures, the required signals can be captured from the drop ports. As shown in Fig. 31a, a wide-spectrum source (amplified spontaneous emission, ASE) was used to couple a multiple-wavelength source into the device. An optical spectrum analyzer (OSA) was used to record the transmission spectra from eight filtering channels at the corresponding drop ports. Besides, multi-channel voltages generated by the analog output module (PXI-6704-NI) were applied to the AOSP chip, in order to tune the operating center wavelengths of eight filtering channels to be consistent with the channel interval of 100 GHz as defined by International Telecommunication Union (ITU). The transmission spectra at eight drop ports were depicted in Fig. 31b, which indicated that the 3-dB bandwidths of eight filtering channels were all around 100 GHz. Besides, the corresponding center wavelengths were at 1547.72 nm, 1549.32 nm, 1550.92 nm, 1552.52 nm, 1554.13 nm, 1555.75 nm, 1557.36 nm, and 1558.98 nm, respectively. Moreover, the crosstalk between the neighboring channels was over 10 dB, which means that it can be used for 100 Gb/s DPSK signal filtering.

**Fig. 32:**
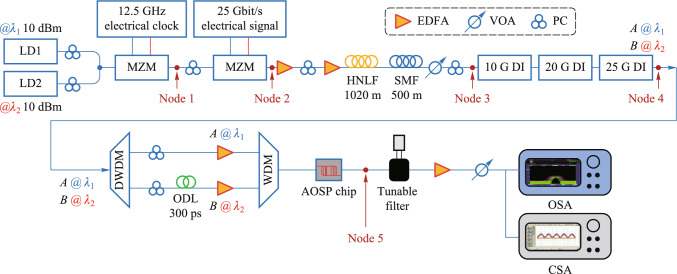
Experimental setup for “logical operation” function of the proposed AOSP chip. *LD* luminescent diode, *PC* polarization controller, *MZM* Mach–Zehnder modulator, *EDFA* erbium-doped fiber amplifier, *HNLF* highly nonlinear fiber, *SMF* single-mode fiber, *VOA* variable optical attenuator, *DI* delay interferometer, *DWDM* dense wavelength division multiplexing, *OSA* optical spectrum analyzer, *CSA* communications signal analyzer

**Fig. 33:**
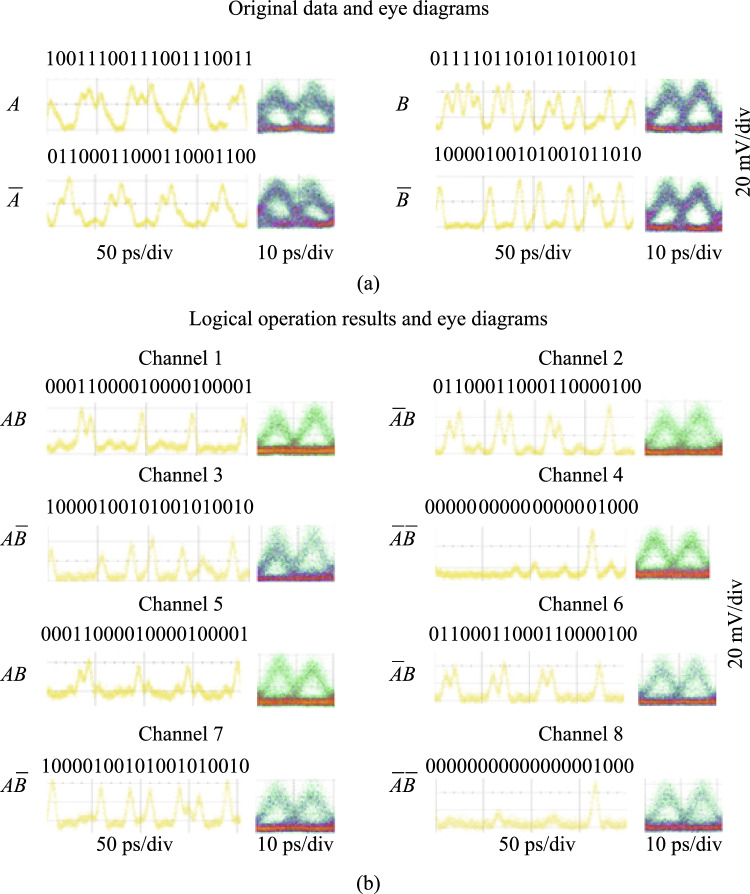
**a** Original data and the corresponding eye diagrams. **b** Logical operation results by the proposed AOSP chip and the corresponding eye diagrams

In the following test, the logical operation function of the proposed AOSP chip was measured. The corresponding experimental setup was shown in Fig. 32. The basic logic gates of two inputs (named as *A* and *B*) including *AB*, $\overline{A }$*B*, *A*$\overline{B }$, $\overline{A }\overline{B }$ were demonstrated, which relied on nonlinear FWM effect caused by the designed reverse-biased p–i–n junction in silicon waveguides. Two optical continuous waves (CWs) with the power of 10 dBm were generated. After adjusting the polarization states of each branch, these two CW lights were combined by an optical coupler. Subsequently, it was modulated by two Mach–Zehnder modulators (MZMs) with the electrical clock of 12.5 GHz and the electrical signal data of 25 Gbit/s, corresponding to node 1 and node 2. After the optical amplification by the erbium-doped fiber amplifier (EDFA), the modulated optical signals were propagated into a highly nonlinear fiber (HNLF) with the length of 1020 m and a SMF with the length of 500 m. At node 3, the modulated optical signals were compressed. Additionally, they could be further multiplexed to be 100 Gb/s by three cascaded optical delay interferometers (DIs). The dense wavelength division multiplexing (DWDM) was used to distinguish two different wavelengths. Hence, two optical signals at different wavelengths were separated. To synchronize these two signals, a tunable ODL was introduced. Moreover, each optical signal was further amplified and coupled by a WDM. After that, they were coupled into the proposed AOSP chip. Based on on-chip four-wave mixing (FWM) effect [177], the corresponding logical operation could be manipulated. Finally, the generated idler light was filtered and captured by the communications signal analyzer (CSA). By tuning the wavelengths of two CW lights and choosing the different operation channels of the AOSP chip, eight different logical operations were realized. As shown in Fig. 33a, two electrical signals named as “*A*” and “*B*” were modulated to two CW lights as the original data. The results including eight different logical operations generated by the proposed AOSP chip were all tested, as illustrated in Fig. 33b. Meanwhile, the corresponding eye diagrams were also captured, verifying that the proposed eight-channel AOSP chip can realize the logical operations at the speed of 100 Gb/s. Besides, the widths of all eye diagrams were set as 10 ps.

**Fig. 34:**
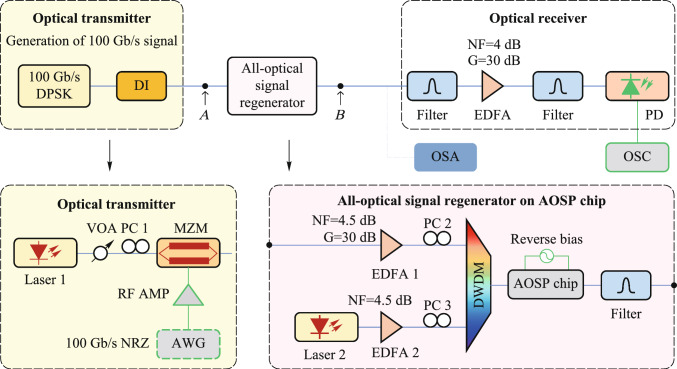
Experimental setup for “signal regeneration” function of the proposed AOSP chip. *DPSK* differential phase shift keying, *DI* delay interferometer, *EDFA* erbium-doped fiber amplifier, *PD* photodetector, *OSA* optical spectrum analyzer, *OSC* oscilloscope, *VOA* variable optical attenuator, *PC* polarization controller, *MZM* Mach–Zehnder modulator, *RF AMP* radio-frequency amplifier, *AWG* arbitrary waveform generator, *NRZ* non-return to zero, *DWDM* dense wavelength division multiplexing

**Fig. 35:**
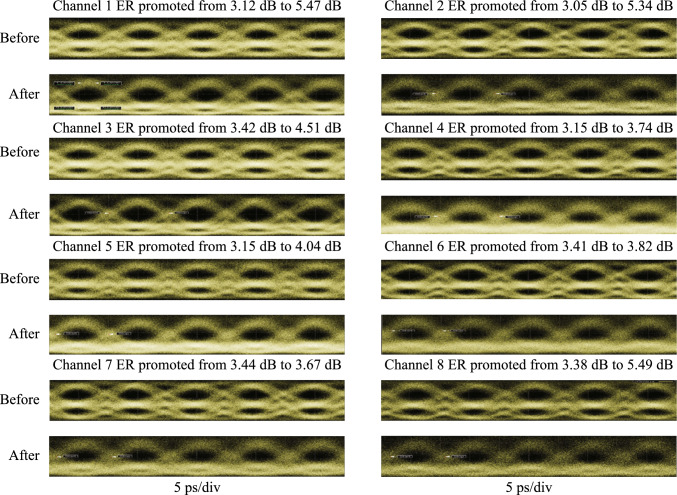
Measured eye diagrams of eight channels before and after signal regeneration

Furthermore, the all-optical signal regeneration function of the proposed AOSP chip was tested. It is worth noting that all-optical signal regeneration function with high speed is widely required to restore the quality of signal data in the optical channel, in which signal propagation impairments are inevitably caused by nonlinearity, dispersion and noise. In our proposed AOSP chip, the nonlinear propagation loss resulted by FCA are reduced by the designed silicon-integrated reverse-biased p–i–n junction. Figure 34 showed the experimental implementations. In the optical transmitter, a 100 Gb/s non-return to zero (NRZ) signal was generated by an arbitrary waveform generator (AWG), which was amplified by a radio-frequency amplifier (RF AMP) and modulated onto the light from a tunable laser by using the MZM. Besides, a VOA was used to adjust the optical power of the original data signal light. Moreover, the modulated DPSK signal could be converted into on-and-off keying (OOK) signal by a variable DI. In the all-optical signal regenerator, the degraded original data signal light was combined with the pump light from another tunable laser 2 via a DWDM, and these two lights were launched into the silicon-based AOSP chip simultaneously. Two polarization controllers (PCs) were utilized to adjust the states of the polarization (SOPs) of these two lights to obtain optimal coupling efficiency. In addition, the optical powers of these two lights were also adjusted by two EDFAs whose noise figure (NF) were both 4.5 dB. As for the optical receiver, the regenerated data signal was selected by an optical bandpass filter 1 with the bandwidth of 200 GHz. Subsequently, it was amplified by an EDFA with NF of 4 dB and further filtered by another optical bandpass filter to reduce the receiving noise. Detected by a photodetector (PD), the extinction ratio (ER) and the quality factor *Q* of the original data signal together with the regenerated data signal were analyzed by a digital oscilloscope (OSC). It is worth mentioning that the node *A* in the propagation link represented back-to-back (B2B) transmission point, also corresponding to the signal before regeneration. Besides, the node *B* was corresponding to the signal after regeneration. By comparing the measured eye diagrams at *A* and *B*, the performances of the all-optical signal regeneration function were evaluated in detail, as shown in Fig. 35. By tuning the lasing wavelengths in the optical transmitter and switching the operation channels of the AOSP chip, the corresponding signal regenerations in each channel were all measured. It is clear that after the signal regeneration in the proposed AOSP chip, the ERs of the regenerated signals in each channel were all promoted.

As listed in Table 8, we compared our work with previous reported signal processors and extracted the key performances of those work. Our work demonstrated a silicon-based eight-channel AOSP chip with multiple functions including filtering, logical operation and signal regeneration. The total capacity of this chip was up to 800 Gb/s, where the speed of each channel demonstrated up to 100 Gb/s. Meanwhile, it can support two kinds of modulated format, including DPSK and OOK. Considering its characteristics of compact footprint, high-density, large capacity, fast processing speed, multi-channel and multi-functional operation with low power consumption, the proposed AOSP chip showcase the potential for applications in the next generation information network.

**Table 8:** Performance Comparison of the reported signal processors

Refs.	Number of wavelength channels	Speed of each channel	Total capacity	Signal format	Functions	Material	Year
[178]	16	–	–	–	Filtering	Silicon	2022
[179]	1	10 Gb/s	10 Gb/s	OOK	Logical operation	Fiber	2022
[180]	1	20 Gb/s	20 Gb/s	QPSK	Logical operation	Silicon	2016
[146]	16	10 Gb/s	160 Gb/s	DPSK	Signal regeneration	Fiber	2018
[147]	1	10 Gb/s	20 Gb/s	PAM4	Signal regeneration	Silicon	2016
This work	8	100 Gb/s	800 Gb/s	DPSK/ OOK	Filtering/logical operation/signal regeneration	Silicon	2023

#### Advanced optoelectronic packaging technology

To realize the monolithically integrated multi-channel and multi-functional AOSP chip, the optoelectronic integrated packaging technology with high density was developed. Several key issues are addressed. First, the coupling efficiency of AOSP chip is critical to the overall performance of the AOSP chips. Ultra-compact fiber-to-chip metamaterial edge coupler as well as different optical packaging methods were developed to promote the optical coupling between the integrated chip and external fibers. Meanwhile, the chip-level and module-level thermal analysis were carried out to optimize the temperature and heat dissipation of the integrated chips. The indispensable electrical packaging was also demonstrated. These technologies are elaborated in the following.

1. Optical coupling and packaging

**Fig. 36:**
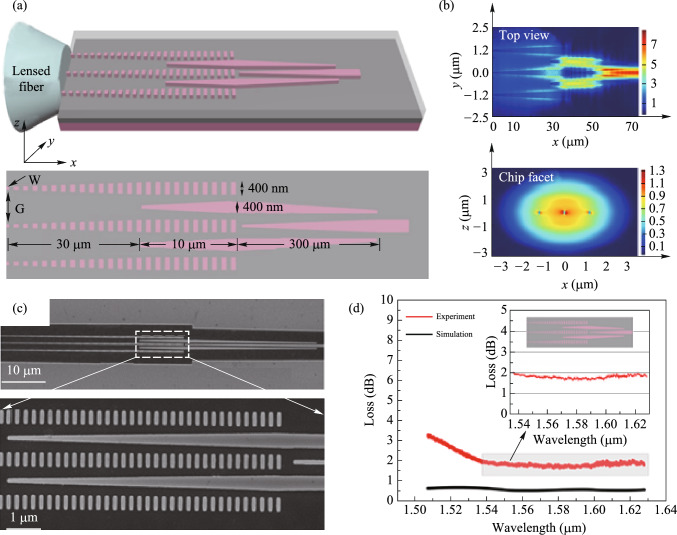
**a** Structure of the SWG metamaterial edge coupler for lensed fiber. **b** Corresponding electrical filed distribution. **c** Optical microscope photographs. **d** Transmission spectra of the SWG metamaterial edge coupler in simulation and measurement; the inset indicates the spectra of 2-dB bandwidth from 1536 to 1628 nm

In this section, two kinds of ultracompact metamaterial edge couplers based on subwavelength grating (SWG) [181] for lensed fiber and SMF with an eased fabrication process were experimentally demonstrated, respectively. Both of them were fully based on the standard fabrication process on a SOI platform. The couplers were fabricated by a single step of electron beam lithography (EBL) and inductively coupled plasma-reactive ion etching (ICP-RIE) process. The structure of the proposed SWG metamaterial edge coupler for lensed fiber was illustrated in Fig. 36a. The corresponding electrical filed distributions from the top view and the chip facet were both analyzed by the Lumerical 3D-FDTD, as shown in Fig. 36b. Besides, the corresponding optical microscope photographs and transmission spectra of the SWG metamaterial edge coupler in simulation and measurement were depicted in Fig. 36c and d. It was shown that the simulated propagation loss at 1550 nm is 0.5 dB. The minimum loss of simulated results was at 1566 nm, corresponding to 0.48 dB. In addition, among the operating wavelength range over 121 nm, the fluctuation was only 0.1 dB. Compared with the simulated results, the tested propagation loss at 1550 nm is 1.82 dB. Due to the inevitable fabrication error, the minimum loss was located at the wavelength of 1581 nm. Moreover, the tested loss was less than 2.0 dB from 1536 to 1628 nm, which indicated that the 2-dB bandwidth of the proposed metamaterial edge coupler for lensed fiber was 92 nm. Furthermore, the fluctuation among the 2-dB bandwidth was only 0.4 dB, which also verified that the proposed edge coupler had a good stability of the operating wavelength.

**Fig. 37:**
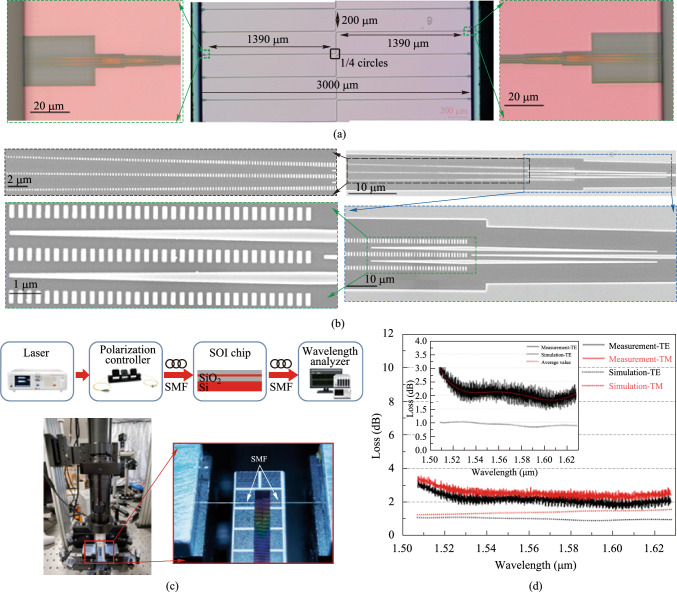
**a** Optical microscope photographs of the diced single chip and **b** the scanning electron microscope photographs of the fabricated SWG metamaterial edge coupler. **c** Experimental setup. **d** Transmission spectra of the SWG metamaterial edge coupler in simulation and measurement; the inset indicates the spectra of the TE_0_ mode

**Fig. 38:**
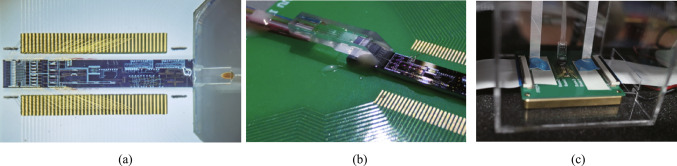
Optical packaging including **a** edge coupling, **b** horizontal grating coupling with the angle of 41$^\circ$, and **c** vertical grating coupling with the angle of 8$^\circ$

In addition, another metamaterial edge coupler based on the optimized SWG structure for SMF was proposed and fabricated, as shown in Fig. 37. The experimental results show that this metamaterial-based coupler possessed low coupling loss and broad bandwidth simultaneously with the coupling length of only 90 $\upmu$m. At 1550 nm, the coupling losses were 2.22/2.53 dB/facet for the fundamental TE/TM mode, while the minimum average loss could reach 1.81 dB/facet. The measured bandwidth with a loss below 3 dB was as broad as 120 nm, covering the entire C/L band. Moreover, this prominently eased fabrication process potentially exhibits significant superiority in both research and industrial applications. To satisfy different requirements of the integrated chips, three different kinds of optical packaging technology including edge coupling, horizontal grating coupling with the angle of 41° and vertical grating coupling with the angle of 8° were all developed, as shown in Fig. 38.

(2) Electrical packaging

**Fig. 39:**
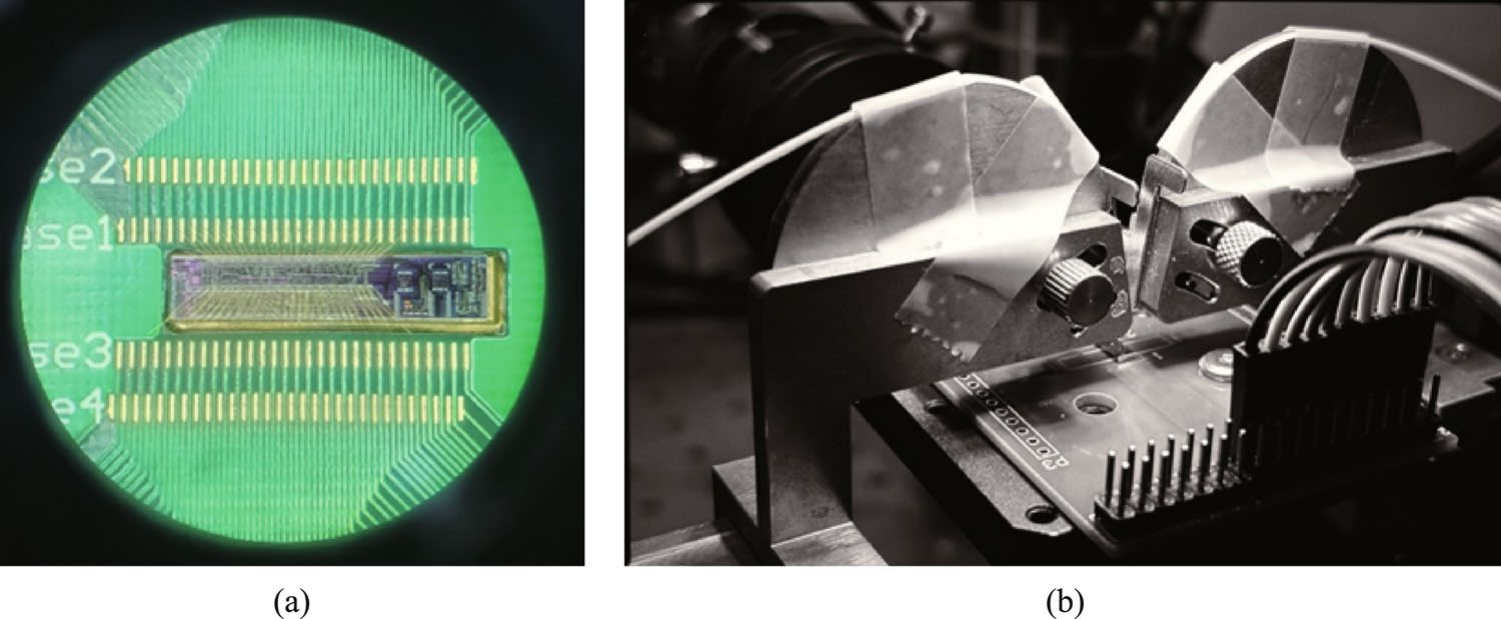
Electrical packaging including **a** wire-bonding and **b** custom-defined printed circuit board

It is worth noting that the integrated multi-channel and multi-functional chips with high density require a large number of electrical pads to connect with the off-chip voltage sources. As the scale of integration increases, multi-line and multi-layer wire-bonding between the on-chip electrical pads and custom-defined printed circuit board (PCB) is required, as shown in Fig. 39. Besides, rules of the wire-bonding technology for direct current (DC) or high-frequency current are different. Especially, electrical crosstalk between high-frequency transmission lines would have impact on the operating bandwidth and power consumption of the integrated chips. Hence, more analysis of the electrical parameters like parasitic capacitance, microwave refractive index is necessary. Meanwhile, the electrical packaging is always developed before the optical packaging, in order to ensure that these two operations would not interact on each other.

(3) Thermal analysis

**Fig. 40:**
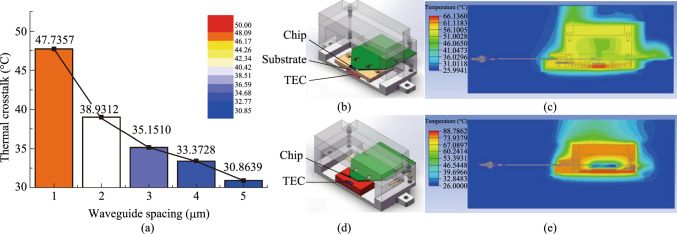
**a** Simulated results of the on-chip thermal crosstalk. **b** Hybrid package with the substrate and **c** the corresponding thermal analysis. **d** Hybrid package without the substrate and **e** the corresponding thermal analysis. TEC, thermo-electric cooler

Thermal crosstalk of the silicon-based chips with high integration is prominent. The thermal resistance of common optoelectronic devices is about 10 K/mW, which makes it difficult to realize the larger scale integration with high density, fast speed and various functions. To solve the heat dissipation of the integrated chip, the effect of heat conduction and convective heat transfer should be considered [182]. The corresponding simulated results of the on-chip thermal crosstalk were depicted in Fig. 40a. Setting the power of the single channel waveguide at 10 mW, the thermal crosstalk between the neighboring waveguides would be decreased gradually as the space was increased. Until the space was increased to 5 $\upmu$m, the on-chip temperature would be acceptable. It is noted that thermo-electric cooler (TEC) module is necessary to maintain the on-chip temperature and reduce the thermal crosstalk. Besides, we use the tungsten copper alloy as the material of overall hybrid packaging structure, as shown in Fig. 40b and d. Moreover, it can be found that the substrate between the integrated chip and the TEC module can conduct the on-chip temperature effectively and evenly, as illustrated in Fig. 40c and e.

(4) Demonstration of intelligent photonic routing

**Fig. 41:**
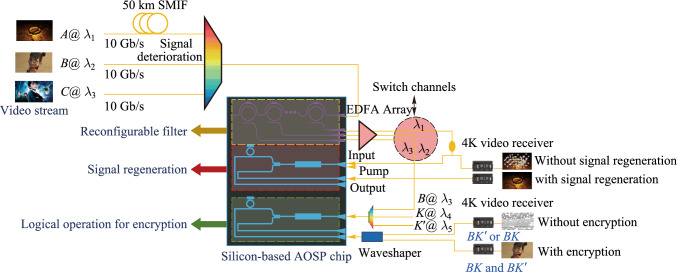
Experimental setup of demonstration for photonic routing

Finally, the demonstration of intelligent photonic routing based on the packaged multi-channel and multi-functional AOSP Chip was implemented, as shown in Fig. 41. Multi-channel 4 K video streams were adopted as the modulated signals for the inputs of the network. Based on the application of three-channel 4 K video streams, three different functions including reconfigurable filtering, signal regeneration and logical operation for encryption in the packaged AOSP chip were achieved in real-time.

In the optical transmitter, three-channel 4 K video streams were modulated into 10 Gb/s data streams named as *A*, *B*, *C*, corresponding to the wavelengths of ${\lambda }_{1}$, ${\lambda }_{2}$, ${\lambda }_{3}$. The data stream *A* modulated to the wavelength of ${\lambda }_{1}$ was sent into an SMF of 50 km length. The original data signal light would become degraded after it transmitted through a long distance. After combined by the DWDM, these three data streams were coupled into the silicon-based AOSP chip. As for the function of reconfigurable filtering, adjusting the applied voltages on each filtering unit would change the operating center wavelengths of the filters. Hence, the output ports for these three data streams could be switched, which verified that the reconfigurable filter was realized. After the filtering units, three data streams were dropped and coupled into the off-chip EDFA array for amplification, which was used to compensate for the losses caused by the on-chip filtering. Besides, in order to demonstrate the function of signal regeneration, the data stream A was split into two branches by the optical coupler. One of the branches was captured by the 4 K video receiver directly. However, the modulated video stream could not be analyzed due to the signal degradation. The other one combined with a pump light was coupled into the silicon-based AOSP chip for the signal regeneration based on the on-chip FWM effect. After that, the output was measured by the 4 K video receiver. After regeneration, the original data stream *A* could be decoded and this video stream could be played. Additionally, we chose the data stream *B* modulated to the wavelength of ${\lambda }_{2}$ as an example, so as to demonstrate the function of logical operation. The encryption key named as *K* was modulated to the wavelength of ${\lambda }_{4}$. Besides, the corresponding inverse code named as *K′* was modulated to the wavelength of ${\lambda }_{5}$. Subsequently, the data stream B, the encryption key *K* and its inverse code *K*′ were combined by the DWDM and further coupled into the integrated AOSP chip. Based on the on-chip FWM effect, idler lights named as *BK* and *BK*′ were generated, respectively. After the on-chip logical operation, the output light was sent into the off-chip wave shaper for filtering. If only one idler light *BK* or *BK*′ was captured, the 4 K video receiver could not receive the complete signals. Therefore, the video stream could not be played. If the idler lights *BK* and *BK*′ were both captured, which means that the output signal was equal to *BK* + *BK*′, the video stream could be decoded by the 4 K video receiver. It indicated that the AOSP chip can realize the logical operation for signal encryption.

### Conclusion

In conclusion, we have demonstrated a silicon-based multi-channel and multi-functional AOSP chip, together with advanced optoelectronic packaging technology. With 136 optical/electrical devices monolithically integrated together on the fabricated AOSP chip, three different functions including reconfigurable filtering, signal regeneration and logical operation were all experimentally implemented. Besides, intelligent photonic routing for multi-channel 4 K video streams was adopted to verify the chip characteristics and functions in real-time.

## Summary and outlook

AOSP holds great potential to overcome the bandwidth limitations inherent in electrical signal processing while significantly reducing both cost and energy consumption. This work highlights key advancements in the development of reconfigurable AOSP chips. Key challenges in building large-scale integrated AOSP photonic chips, such as high transmission losses, weak nonlinear effects, limited optical field control, and severe optical, electrical, and thermal crosstalk, have been addressed through structural and material innovations. Ultra-low loss silicon waveguides are developed with losses as low as 0.17 dB/cm, resulting in *Q* factor up to 2.1 $\times$ 10^6^. Advanced integrated filters with bandwidth tunable from 0.55 to 648.72 pm (i.e., tuned over three order of magnitude), as well as FSR tunable from 0.06 nm to 1.86 nm (30 times) have been realized. The absolute FWM conversion efficiencies have been demonstrated up to − 12 dB, where such a high efficiency is of great importance to ensure the success of high-performance logic and regeneration operation. Eight-channel multifunctional monolithic integration of filtering, logic, and regeneration has been achieved, with 136 devices (including filters, logic gates, regenerators, gratings, MMIs, electrodes, etc.) integrated on a single chip. A total signal processing capacity of up to 800 Gb/s (each channel operated at 100 Gb/s) has been demonstrated, accommodating multiple modulation formats, including DPSK and OOK. Full set of CLUs are generated for logic operations and over 6 dB improvement of receiver sensitivity has been demonstrated for QPSK regeneration. With advanced optical and electrical packaging techniques, a chip-scale routing and processing of multi-channel signals has been verified. Since optical Kerr nonlinearity is intrinsically ultrafast (on the time-scale of femtosecond), these efforts lay the foundation for the design and fabrication of large-scale silicon-based AOSP chips toward higher speeds. Looking ahead, improvements in nanofabrication techniques, novel materials, and packaging processes are expected to further enhance the performance and flexibility of AOSP chips, paving the way for transformative applications in optical communications, high-performance computing, imaging, and sensing.

## Data Availability

The data that support the findings of this study are available from the corresponding author, upon reasonable request.

## References

[1] Ji, Y., Wang, H., Cui, J., Yu, M., Yang, Z., Bai, L.: All-optical signal processing technologies in flexible optical networks. Photonic Netw. Commun. **38**(1), 14–36 (2019)

[2] Wabnitz, S., Eggleton, B.J.: All-optical signal processing: data communication and storage applications. Springer International Publishing, Cham (2015)

[3] Ellis, A.D., Zhao, J., Cotter, D.: Approaching the non-linear Shannon limit. J. Lightwave Technol. **28**(4), 423–433 (2010)

[4] Zhong, Z., Wang, H., Ji, Y.: All-optical aggregation and de-aggregation between 3× BPSK and 8QAM in HNLF with wavelength preserved. Appl. Opt. **59**(4), 1092–1098 (2020) 32225247 10.1364/AO.379130

[5] Cui, J., Tan, Y., Lu, G.W., Ji, Y., Wen, H., Zhai, K., Li, M., Zhu, N.: Across-dimensional optical constellation de-aggregations from QAMs to PAMs in optical transparent networks. Opt. Laser Technol. **175**, 110737 (2024)

[6] Dong, J., Zhang, X., Xu, J., Huang, D., Fu, S., Shum, P.: 40 Gb/s all-optical NRZ to RZ format conversion using single SOA assisted by optical bandpass filter. Opt. Expr. **15**(6), 2907 (2007) 10.1364/oe.15.002907 19532526

[7] Fu, S., Zhong, W.D., Shum, P.P., Wen, Y.J.: All-optical NRZ-OOK-to-RZ-OOK format conversions with tunable duty cycles using nonlinear polarization rotation of a semiconductor optical amplifier. Opt. Commun. **282**(11), 2143–2146 (2009)

[8] Slavík, R., Parmigiani, F., Kakande, J., Lundström, C., Sjödin, M., Andrekson, P.A., Weerasuriya, R., Sygletos, S., Ellis, A.D., Grüner-Nielsen, L., Jakobsen, D., Herstrøm, S., Phelan, R., O’Gorman, J., Bogris, A., Syvridis, D., Dasgupta, S., Petropoulos, P., Richardson, D.J.: All-optical phase and amplitude regenerator for next-generation telecommunications systems. Nat. Photonics **4**(10), 690–695 (2010)

[9] Kakande, J., Slavík, R., Parmigiani, F., Bogris, A., Syvridis, D., Grüner-Nielsen, L., Phelan, R., Petropoulos, P., Richardson, D.J.: Multilevel quantization of optical phase in a novel coherent parametric mixer architecture. Nat. Photonics **5**(12), 748–752 (2011)

[10] Yang, Z., Dong, W., Fan, Z., He, S., Chen, N., Li, H., Zhou, H., Zhang, X., Xu, J.: 40 Gb/s multimode all-optical regenerator based on the low-loss silicon-based nanowaveguide. Opt. Expr. **32**(4), 6507 (2024) 10.1364/OE.508059 38439351

[11] Chitgarha, M.R., Khaleghi, S., Yilmaz, O.F., Tur, M., Haney, M.W., Langrock, C., Fejer, M.M., Willner, A.E.: Demonstration of channel-spacing-tunable demultiplexing of optical orthogonal-frequency-division-multiplexed subcarriers utilizing reconfigurable all-optical discrete Fourier transform. Opt. Lett. **37**(19), 3975 (2012) 23027250 10.1364/OL.37.003975

[12] Marpaung, D., Pagani, M., Morrison, B., Eggleton, B.J.: Nonlinear integrated microwave photonics. J. Lightwave Technol. **32**(20), 3421–3427 (2014)

[13] Xue, X., Zheng, X., Zhou, B., Weiner, A.M.: Microresonator frequency combs for integrated microwave photonics. IEEE Photonics Technol. Lett. **30**(21), 1814–1817 (2018)

[14] Tur, M., Langrock, C., Almaiman, A., Touch, J.D., Khaleghi, S., Chitgarha, M.R., Mohajerin-Ariaei, A., Fejer, M.M., Ziyadi, M., Willner, A.E.: Reconfigurable 2-D WDM optical tapped-delay-line to correlate 20 Gbaud QPSK data. In: 39th European conference and exhibition on optical communication (ECOC 2013), London, UK (2013)

[15] Khaleghi, S., Chitgarha, M.R., Ziyadi, M., Daab, W., Mohajerin-Ariaei, A., Rogawski, D., Touch, J.D., Tur, M., Langrock, C., Fejer, M.M., Willner, A.E.: A tunable optical tapped-delay-line that simultaneously and independently processes multiple input WDM data signals. In: Optical fiber communication conference/national fiber optic engineers conference 2013. OSA, Anaheim, California (2013)

[16] Gu, W., Gao, X., Dong, W., Wang, Y., Zhou, H., Xu, J., Zhang, X.: All-optical complex-valued convolution based on four-wave mixing. Optica **11**(1), 64 (2024)

[17] Khaleghi, S., Yilmaz, O.F., Chitgarha, M.R., Tur, M., Ahmed, N., Nuccio, S.R., Fazal, I.M., Wu, X., Haney, M.W., Langrock, C., Fejer, M.M., Willner, A.E.: High-speed correlation and equalization using a continuously tunable all-optical tapped delay line. IEEE Photonics J. **4**(4), 1220–1235 (2012)

[18] Vukovic, N., Healy, N., Suhailin, F.H., Mehta, P., Day, T.D., Badding, J.V., Peacock, A.C.: Ultrafast optical control using the Kerr nonlinearity in hydrogenated amorphous silicon microcylindrical resonators. Sci. Rep. **3**(1), 2885 (2013) 24097126 10.1038/srep02885 PMC3791441

[19] Chai, Z., Hu, X., Wang, F., Niu, X., Xie, J., Gong, Q.: Ultrafast all-optical switching. Adv. Opt. Mater. **5**(7), 1600665 (2017)

[20] Steiner, T.J., Castro, J.E., Chang, L., Dang, Q., Xie, W., Norman, J., Bowers, J.E., Moody, G.: Ultrabright entangled-photon-pair generation from an AlGaAs-on-insulator microring resonator. PRX Quantum **2**(1), 010337 (2021)

[21] Ma, Z., Chen, J.Y., Li, Z., Tang, C., Sua, Y.M., Fan, H., Huang, Y.P.: Ultrabright quantum photon sources on chip. Phys. Rev. Lett. **125**(26), 263602 (2020) 33449782 10.1103/PhysRevLett.125.263602

[22] Lin, Q., Agrawal, G.P.: Silicon waveguides for creating quantum-correlated photon pairs. Opt. Lett. **31**(21), 3140 (2006) 17041661 10.1364/ol.31.003140

[23] Imany, P., Jaramillo-Villegas, J.A., Odele, O.D., Han, K., Leaird, D.E., Lukens, J.M., Lougovski, P., Qi, M., Weiner, A.M.: 50-GHz-spaced comb of high-dimensional frequency-bin entangled photons from an on-chip silicon nitride microresonator. Opt. Expr. **26**(2), 1825 (2018) 10.1364/OE.26.001825 29401906

[24] Yu, C., Christen, L., Luo, T., Wang, Y., Pan, Z., Yan, L.S., Willner, A.E.: All-optical XOR gate using polarization rotation in single highly nonlinear fiber. IEEE Photonics Technol. Lett. **17**(6), 1232–1234 (2005)

[25] Hajomer, A.A.E., Presi, M., Andriolli, N., Porzi, C., Hu, W., Contestabile, G., Yang, X.: On-chip all-optical wavelength conversion of PAM-4 signals using an integrated SOA-based turbo-switch circuit. J. Lightwave Technol. **37**(16), 3956–3962 (2019)

[26] Bogoni, A., Wu, X., Fazal, I., Willner, A.E.: 160 Gb/s Time-domain channel extraction/insertion and all-optical logic operations exploiting a single PPLN waveguide. J. Lightwave Technol. **27**(19), 4221–4227 (2009)

[27] Shen, J., Yu, S., Liao, P., Chen, Z., Gu, W., Guo, H.: All-optical full-adder based on cascaded PPLN waveguides. IEEE J. Quantum Electron. **47**(9), 1195–1200 (2011)

[28] Borghi, M., Castellan, C., Signorini, S., Trenti, A., Pavesi, L.: Nonlinear silicon photonics. J. Opt. **19**(9), 093002 (2017)

[29] Ye, Z., Zhao, P., Twayana, K., Karlsson, M., Torres-Company, V., Andrekson, P.A.: Overcoming the quantum limit of optical amplification in monolithic waveguides. Sci. Adv. **7**(38), eabi8150 (2021) 34524857 10.1126/sciadv.abi8150 PMC8443169

[30] Razzari, L., Duchesne, D., Ferrera, M., Morandotti, R., Chu, S., Little, B.E., Moss, D.J.: CMOS-compatible integrated optical hyper-parametric oscillator. Nat. Photonics **4**(1), 41–45 (2010)

[31] Xia, D., Yang, Z., Zeng, P., Zhang, B., Wu, J., Wang, Z., Zhao, J., Huang, J., Luo, L., Liu, D., Yang, S., Guo, H., Li, Z.: Integrated chalcogenide photonics for microresonator soliton combs. Laser Photonics Rev. **17**(3), 2200219 (2023)

[32] Pu, M., Hu, H., Ottaviano, L., Semenova, E., Vukovic, D., Oxenløwe, L.K., Yvind, K.: Ultra-efficient and broadband nonlinear AlGaAs-on-insulator chip for low-power optical signal processing. Laser Photonics Rev. **12**(12), 1800111 (2018)

[33] Stassen, E., Kim, C., Kong, D., Hu, H., Galili, M., Oxenløwe, L.K., Yvind, K., Pu, M.: Ultra-low power all-optical wavelength conversion of high-speed data signals in high-confinement AlGaAs-on-insulator microresonators. APL Photonics **4**(10), 100804 (2019)

[34] Chang, L., Xie, W., Shu, H., Yang, Q.F., Shen, B., Boes, A., Peters, J.D., Jin, W., Xiang, C., Liu, S., Moille, G., Yu, S.P., Wang, X., Srinivasan, K., Papp, S.B., Vahala, K., Bowers, J.E.: Ultra-efficient frequency comb generation in AlGaAs-on-insulator microresonators. Nat. Commun. **11**(1), 1331 (2020) 32165610 10.1038/s41467-020-15005-5 PMC7067760

[35] Ge, L., Chen, Y., Jiang, H., Li, G., Zhu, B., Liu, Y., Chen, X.: Broadband quasi-phase matching in a MgO:PPLN thin film. Photon. Res. **6**(10), 954 (2018)

[36] Wei, J., Hu, Z., Zhang, M., Li, P., Wu, Y., Zeng, C., Tang, M., Xia, J.: All-optical wavelength conversion of a 92-Gb/s 16-QAM signal within the C-band in a single thin-film PPLN waveguide. Opt. Expr. **30**(17), 30564 (2022) 10.1364/OE.465382 36242157

[37] Jalali, B., Yegnanarayanan, S., Yoon, T., Yoshimoto, T., Rendina, I., Coppinger, F.: Advances in silicon-on-insulator optoelectronics. IEEE J. Sel. Top. Quantum Electron. **4**(6), 938–947 (1998)

[38] Bogaerts, W., Baets, R., Dumon, P., Wiaux, V., Stephan, B., Taillaert, D., Luyssaert, B., Campenhout, J.V., Bienstman, P., Thourhout, D.V.: Nanophotonic waveguides in silicon-on-insulator fabricated with CMOS technology. J. Lightwave Technol. **23**(1), 401–412 (2005)

[39] Pavesi, L.: Thirty years in silicon photonics: a personal view. Front. Phys. (Lausanne) **9**, 786028 (2021)

[40] Bell, M.I.: Frequency dependence of Miller’s rule for nonlinear susceptibilities. Phys. Rev. B Solid State **6**(2), 516–521 (1972)

[41] Boes, A., Chang, L., Langrock, C., Yu, M., Zhang, M., Lin, Q., Lončar, M., Fejer, M., Bowers, J., Mitchell, A.: Lithium niobate photonics: Unlocking the electromagnetic spectrum. Science **379**(6627), eabj4396 (2023) 36603073 10.1126/science.abj4396

[42] Ding, M., Zhang, M., Hong, S., Zhao, Y., Zhang, L., Wang, Y., Chen, H., Yu, Z., Gao, S., Dai, D.: High-efficiency four-wave mixing in low-loss silicon photonic spiral waveguides beyond the singlemode regime. Opt. Expr. **30**(10), 16362 (2022) 10.1364/OE.456704 36221480

[43] Riemensberger, J., Kuznetsov, N., Liu, J., He, J., Wang, R.N., Kippenberg, T.J.: A photonic integrated continuous-travelling-wave parametric amplifier. Nature **612**(7938), 56–61 (2022) 36450905 10.1038/s41586-022-05329-1

[44] Xuan, Y., Liu, Y., Varghese, L.T., Metcalf, A.J., Xue, X., Wang, P.H., Han, K., Jaramillo-Villegas, J.A., Noman, A.A., Wang, C., Kim, S., Teng, M., Lee, Y.J., Niu, B., Fan, L., Wang, J., Leaird, D.E., Weiner, A.M., Qi, M.: High-*Q* silicon nitride microresonators exhibiting low-power frequency comb initiation. Optica **3**(11), 1171 (2016)

[45] Li, F., Pelusi, M., Xu, D.X., Ma, R., Janz, S., Eggleton, B.J., Moss, D.J.: All-optical wavelength conversion for 10 Gb/s DPSK signals in a silicon ring resonator. Opt. Expr. **19**(23), 22410 (2011) 10.1364/OE.19.022410 22109117

[46] Monat, C., Grillet, C., Collins, M., Clark, A., Schroeder, J., Xiong, C., Li, J., O’Faolain, L., Krauss, T.F., Eggleton, B.J., Moss, D.J.: Integrated optical auto-correlator based on third-harmonic generation in a silicon photonic crystal waveguide. Nat. Commun. **5**(1), 3246 (2014) 24496243 10.1038/ncomms4246

[47] Almeida, V.R., Xu, Q., Barrios, C.A., Lipson, M.: Guiding and confining light in void nanostructure. Opt. Lett. **29**(11), 1209 (2004) 15209249 10.1364/ol.29.001209

[48] Kauranen, M., Zayats, A.V.: Nonlinear plasmonics. Nat. Photonics **6**(11), 737–748 (2012)

[49] Abir, T., Tal, M., Ellenbogen, T.: Second-harmonic enhancement from a nonlinear plasmonic metasurface coupled to an optical waveguide. Nano Lett. **22**(7), 2712–2717 (2022) 35369689 10.1021/acs.nanolett.1c04584 PMC9011386

[50] Koos, C., Vorreau, P., Vallaitis, T., Dumon, P., Bogaerts, W., Baets, R., Esembeson, B., Biaggio, I., Michinobu, T., Diederich, F., Freude, W., Leuthold, J.: All-optical high-speed signal processing with silicon–organic hybrid slot waveguides. Nat. Photonics **3**(4), 216–219 (2009)

[51] Wang, Y., He, S., Gao, X., Ye, P., Lei, L., Dong, W., Zhang, X., Xu, P.: Enhanced optical nonlinearity in a silicon–organic hybrid slot waveguide for all-optical signal processing. Photon. Res. **10**(1), 50–58 (2022)

[52] Dong, P., Qian, W., Liao, S., Liang, H., Kung, C.C., Feng, N.N., Shafiiha, R., Fong, J., Feng, D., Krishnamoorthy, A.V., Asghari, M.: Low loss shallow-ridge silicon waveguides. Opt. Expr. **18**(14), 14474–14479 (2010) 10.1364/OE.18.014474 20639932

[53] Ji, X., Barbosa, F.A.S., Roberts, S.P., Dutt, A., Cardenas, J., Okawachi, Y., Bryant, A., Gaeta, A.L., Lipson, M.: Ultra-low-loss on-chip resonators with sub-milliwatt parametric oscillation threshold. Optica **4**(6), 619–624 (2017)

[54] Puckett, M.W., Liu, K., Chauhan, N., Zhao, Q., Cheng, H., Blumenthal, D.J.: 422 Million intrinsic quality factor planar integrated all-waveguide resonator with sub-MHz linewidth. Nat. Commun. **12**(1), 934 (2021) 33568661 10.1038/s41467-021-21205-4 PMC7876138

[55] Qiu, H., Zhou, F., Qie, J., Yao, Y., Hu, X., Zhang, Y., Xiao, X., Yu, Y., Dong, J., Zhang, X.: A continuously tunable sub-gigahertz microwave photonic bandpass filter based on an ultra-high-*Q* silicon microring resonator. J. Lightwave Technol. **36**(19), 4312–4318 (2018)

[56] Guillén-Torres, M. A., Caverley, M., Cretu, E., Jaeger, N. A., Chrostowski, L.: Large-area, high-*Q* SOI ring resonators. In 2014 IEEE photonics conference 336–337 IEEE. (2014)

[57] Zhang, L., Jie, L., Zhang, M., Wang, Y., Xie, Y., Shi, Y., Dai, D.: Ultrahigh-Q silicon racetrack resonators. Photon. Res. **8**(5), 684–689 (2020)

[58] Lee, K.K., Lim, D.R., Kimerling, L.C., Shin, J., Cerrina, F.: Fabrication of ultralow-loss Si/SiO_2_ waveguides by roughness reduction. Opt. Lett. **26**(23), 1888–1890 (2001) 18059727 10.1364/ol.26.001888

[59] Biberman, A., Shaw, M.J., Timurdogan, E., Wright, J.B., Watts, M.R.: Ultralow-loss silicon ring resonators. Opt. Lett. **37**(20), 4236–4238 (2012) 23073422 10.1364/OL.37.004236

[60] Takahashi, J.I., Tsuchizawa, T., Watanabe, T., Itabashi, S.I.: Oxidation-induced improvement in the sidewall morphology and cross-sectional profile of silicon wire waveguides. J Vac Sci Technol B Microelectron Nanometer Struct Process Meas Phenom **22**(5), 2522–2525 (2004)

[61] Xie, W., Chang, L., Shu, H., Norman, J.C., Peters, J.D., Wang, X., Bowers, J.E.: Ultrahigh-*Q* AlGaAs-on-insulator microresonators for integrated nonlinear photonics. Opt. Expr. **28**(22), 32894–32906 (2020) 10.1364/OE.405343 33114964

[62] Hung, S.C., Liang, E.Z., Lin, C.F.: Silicon waveguide sidewall smoothing by KrF excimer laser reformation. J. Lightwave Technol. **27**(7), 887–892 (2009)

[63] Zhang, B., Al Qubaisi, K., Cherchi, M., Harjanne, M., Ehrlichman, Y., Khilo, A.N., Popović, M.A.: Compact multi-million *Q* resonators and 100 MHz passband filter bank in a thick-SOI photonics platform. Opt. Lett. **45**(11), 3005–3008 (2020) 32479444 10.1364/OL.395203

[64] Huang, Q., Yu, J.: Coherent interaction between two orthogonal travelling-wave modes in a microdonut resonator for filtering and buffering applications. Opt. Expr. **22**(21), 25171–25182 (2014) 10.1364/OE.22.025171 25401549

[65] Naweed, A.: Photonic coherence effects from dual-waveguide coupled pair of co-resonant microring resonators. Opt. Expr. **23**(10), 12573–12581 (2015) 10.1364/OE.23.012573 26074512

[66] Huang, G., Fu, M., Qi, J., Pan, J., Yi, W., Li, X.: Design of broadband flat optical frequency comb based on cascaded sign-alternated dispersion tellurite microstructure fiber. Micromachines (Basel) **12**(10), 1252 (2021) 34683303 10.3390/mi12101252 PMC8539913

[67] Buscaino, B., Zhang, M., Lončar, M., Kahn, J.M.: Design of efficient resonator-enhanced electro-optic frequency comb generators. J. Lightwave Technol. **38**(6), 1400–1413 (2020)

[68] Zhang, Y., Liu, Q., Mei, C., Zeng, D., Huang, Q., Zhang, X.: Proposal and demonstration of a controllable *Q* factor in directly coupled microring resonators for optical buffering applications. Photon. Res. **9**(10), 2006–2015 (2021)

[69] Elshaari, A.W., Aboketaf, A., Preble, S.F.: Controlled storage of light in silicon cavities. Opt. Expr. **18**(3), 3014–3022 (2010) 10.1364/OE.18.003014 20174132

[70] Vlasov, Y., Green, W.M.J., Xia, F.: High-throughput silicon nanophotonic wavelength-insensitive switch for on-chip optical networks. Nat. Photonics **2**(4), 242–246 (2008)

[71] Qavi, A.J., Washburn, A.L., Byeon, J.Y., Bailey, R.C.: Label-free technologies for quantitative multiparameter biological analysis. Anal. Bioanal. Chem. **394**(1), 121–135 (2009) 19221722 10.1007/s00216-009-2637-8 PMC2667559

[72] Deng, X., Yan, L., Jiang, H., Feng, X., Pan, W., Luo, B.: Polarization-insensitive and tunable silicon Mach-Zehnder wavelength filters with flat transmission passband. IEEE Photonics J. **10**(3), 1–7 (2018)

[73] Gerstel, O., Jinno, M., Lord, A., Yoo, S.B.: Elastic optical networking: a new dawn for the optical layer? IEEE Commun. Mag. **50**(2), s12–s20 (2012)

[74] Zhou, R., Pascual, M.D.G., Anandarajah, P.M., Shao, T., Smyth, F., Barry, L.P.: Flexible wavelength de-multiplexer for elastic optical networking. Opt. Lett. **41**(10), 2241–2244 (2016) 27176972 10.1364/OL.41.002241

[75] Ong, J.R., Kumar, R., Mookherjea, S.: Ultra-high-contrast and tunable-bandwidth filter using cascaded high-order silicon microring filters. IEEE Photonics Technol. Lett. **25**(16), 1543–1546 (2013)

[76] Dai, T., Shen, A., Wang, G., Wang, Y., Li, Y., Jiang, X., Yang, J.: Bandwidth and wavelength tunable optical passband filter based on silicon multiple microring resonators. Opt. Lett. **41**(20), 4807–4810 (2016) 28005898 10.1364/OL.41.004807

[77] Liu, L., Xue, W., Jin, X., Yue, J., Yu, Z., Zhou, L.: Bandwidth and wavelength tunable all-optical filter based on cascaded opto-mechanical microring resonators. IEEE Photonics J. **11**(1), 1–10 (2019)

[78] Poulopoulos, G., Giannoulis, G., Iliadis, N., Kalavrouziotis, D., Apostolopoulos, D., Avramopoulos, H.: Flexible filtering element on SOI with wide bandwidth tunability and full FSR tuning. J. Lightwave Technol. **37**(2), 300–306 (2019)

[79] Orlandi, P., Morichetti, F., Strain, M.J., Sorel, M., Bassi, P., Melloni, A.: Photonic integrated filter with widely tunable bandwidth. J. Lightwave Technol. **32**(5), 897–907 (2014)

[80] Jiang, X., Wu, J., Yang, Y., Pan, T., Mao, J., Liu, B., Liu, R., Zhang, Y., Qiu, C., Tremblay, C., Su, Y.: Wavelength and bandwidth-tunable silicon comb filter based on Sagnac loop mirrors with Mach−Zehnder interferometer couplers. Opt. Expr. **24**(3), 2183–2188 (2016) 10.1364/OE.24.002183 26906794

[81] Liao, S., Ding, Y., Peucheret, C., Yang, T., Dong, J., Zhang, X.: Integrated programmable photonic filter on the silicon-on-insulator platform. Opt. Expr. **22**(26), 31993–31998 (2014) 10.1364/OE.22.031993 25607167

[82] St-Yves, J., Bahrami, H., Jean, P., LaRochelle, S., Shi, W.: Widely bandwidth-tunable silicon filter with an unlimited free-spectral range. Opt. Lett. **40**(23), 5471–5474 (2015) 26625028 10.1364/OL.40.005471

[83] Jiang, J., Qiu, H., Wang, G., Li, Y., Dai, T., Wang, X., Yu, H., Yang, J., Jiang, X.: Broadband tunable filter based on the loop of multimode Bragg grating. Opt. Expr. **26**(1), 559–566 (2018) 10.1364/OE.26.000559 29328333

[84] Mora, J., Chen, L.R., Capmany, J.: Single-bandpass microwave photonic filter with tuning and reconfiguration capabilities. J. Lightwave Technol. **26**(15), 2663–2670 (2008)

[85] Jiang, Y., Shum, P.P., Zu, P., Zhou, J., Bai, G., Xu, J., Zhou, Z., Li, H., Wang, S.: A selectable multiband bandpass microwave photonic filter. IEEE Photonics J. **5**(3), 5500509 (2013)

[86] Zhang, Y., Hu, X., Chen, D., Wang, L., Li, M., Feng, P., Xiao, X., Yu, S.: Design and demonstration of ultra-high-Q silicon microring resonator based on a multi-mode ridge waveguide. Opt. Lett. **43**(7), 1586–1589 (2018) 29601036 10.1364/OL.43.001586

[87] Zhang, L., Hong, S., Wang, Y., Yan, H., Xie, Y., Chen, T., Zhang, M., Yu, Z., Shi, Y., Liu, L., Dai, D.: Ultralow-loss silicon photonics beyond the singlemode regime. Laser Photonics Rev. **16**(4), 2100292 (2022)

[88] Zhang, Y., Zhong, K., Zhou, X., Tsang, H.K.: Broadband high-Q multimode silicon concentric racetrack resonators for widely tunable Raman lasers. Nat. Commun. **13**(1), 3534 (2022) 35725566 10.1038/s41467-022-31244-0 PMC9209424

[89] Chen, L., Sherwood-Droz, N., Lipson, M.: Compact bandwidth-tunable microring resonators. Opt. Lett. **32**(22), 3361–3363 (2007) 18026308 10.1364/ol.32.003361

[90] Liu, M., Zhao, Y., Wang, X., Zhang, X., Gao, S., Dong, J., Cai, X.: Widely tunable fractional-order photonic differentiator using a Mach−Zenhder interferometer coupled microring resonator. Opt. Expr. **25**(26), 33305–33314 (2017)

[91] Xu, J., Zhang, Y., Guo, X., Huang, Q., Zhang, X., Su, Y.: Ultra-narrow passband-tunable filter based on a high-*Q* silicon racetrack resonator. Opt. Lett. **46**(22), 5575–5578 (2021) 34780409 10.1364/OL.443723

[92] Zhang, Y., Zhong, K., Tsang, H.K.: Compact multimode silicon racetrack resonators for high-efficiency tunable Raman lasers. Appl. Phys. Lett. **122**(8), 081101 (2023)

[93] Lakshmijayasimha, P.D., Kaszubowska-Anandarajah, A., Martin, E.P., Hammad, M.N., Landais, P., Anandarajah, P.M.: Characterization of a multifunctional active demultiplexer for optical frequency combs. Opt. Laser Technol. **134**, 106637 (2021)

[94] Bogaerts, W., Pérez, D., Capmany, J., Miller, D.A., Poon, J., Englund, D., Morichetti, F., Melloni, A.: Programmable photonic circuits. Nature **586**(7828), 207–216 (2020) 33028997 10.1038/s41586-020-2764-0

[95] Touch, J., Badawy, A.H., Sorger, V.J.: Optical computing. Nanophotonics **6**(3), 503–505 (2017)

[96] Zhang, L., Ji, R., Jia, L., Yang, L., Zhou, P., Tian, Y., Chen, P., Lu, Y., Jiang, Z., Liu, Y., Fang, Q., Yu, M.: Demonstration of directed XOR/XNOR logic gates using two cascaded microring resonators. Opt. Lett. **35**(10), 1620–1622 (2010) 20479828 10.1364/OL.35.001620

[97] Ying, Z., Feng, C., Zhao, Z., Soref, R., Pan, D., Chen, R.T.: Integrated multi-operand electro-optic logic gates for optical computing. Appl. Phys. Lett. **115**(17), 171104 (2019)

[98] Tian, Y., Zhao, Y., Chen, W., Guo, A., Li, D., Zhao, G., Liu, Z., Xiao, H., Liu, G., Yang, J.: Electro-optic directed XOR logic circuits based on parallel-cascaded micro-ring resonators. Opt. Express **23**(20), 26342–26355 (2015) 26480148 10.1364/OE.23.026342

[99] Tian, Y., Liu, Z., Xiao, H., Zhao, G., Liu, G., Yang, J., Ding, J., Zhang, L., Yang, L.: Experimental demonstration of a reconfigurable electro-optic directed logic circuit using cascaded carrier-injection micro-ring resonators. Sci. Rep. **7**(1), 6410 (2017) 28743874 10.1038/s41598-017-06736-5 PMC5527009

[100] Ying, Z., Wang, Z., Zhao, Z., Dhar, S., Pan, D.Z., Soref, R., Chen, R.T.: Comparison of microrings and microdisks for high-speed optical modulation in silicon photonics. Appl. Phys. Lett. **112**(11), 111108 (2018)

[101] Ying, Z., Wang, Z., Zhao, Z., Dhar, S., Pan, D.Z., Soref, R., Chen, R.T.: Silicon microdisk-based full adders for optical computing. Opt. Lett. **43**(5), 983–986 (2018) 29489761 10.1364/OL.43.000983

[102] Ying, Z., Feng, C., Zhao, Z., Dhar, S., Dalir, H., Gu, J., Chen, R.T.: Electronic-photonic arithmetic logic unit for high-speed computing. Nat. Commun. **11**(1), 2154 (2020) 32358492 10.1038/s41467-020-16057-3 PMC7195421

[103] Feng, C., Ying, Z., Zhao, Z., Gu, J., Pan, D.Z., Chen, R.T.: Wavelength-division-multiplexing (WDM)-based integrated electronic–photonic switching network (EPSN) for high-speed data processing and transportation: High-speed optical switching network. Nanophotonics **9**(15), 4579–4588 (2020)

[104] Feng, C., Ying, Z., Zhao, Z., Gu, J., Pan, D.Z., Chen, R.T.: Toward high-speed and energy-efficient computing: A WDM-based scalable on-chip silicon integrated optical comparator. Laser Photonics Rev. **15**(8), 2000275 (2021)

[105] Xu, J., Zhang, X., Zhang, Y., Dong, J., Liu, D., Huang, D.: Reconfigurable all-optical logic gates for multi-input differential phase-shift keying signals: design and experiments. J. Lightwave Technol. **27**(23), 5268–5275 (2009)

[106] Lei, L., Dong, J., Yu, Y., Tan, S., Zhang, X.: All-optical canonical logic units-based programmable logic array (CLUs-PLA) using semiconductor optical amplifiers. J. Lightwave Technol. **30**(22), 3532–3539 (2012)

[107] Qiu, J., Sun, K., Rochette, M., Chen, L.R.: Reconfigurable all-optical multilogic gate (XOR, AND, and OR) based on cross-phase modulation in a highly nonlinear fiber. IEEE Photonics Technol. Lett. **22**(16), 1199–1201 (2010)

[108] Dai, B., Shimizu, S., Wang, X., Wada, N.: Simultaneous all-optical half-adder and half-subtracter based on two semiconductor optical amplifiers. IEEE Photonics Technol. Lett. **25**(1), 91–93 (2013)

[109] Chen, X., Huo, L., Zhao, Z., Zhuang, L., Lou, C.: Reconfigurable all-optical logic gates using single semiconductor optical amplifier at 100-Gb/s. IEEE Photonics Technol. Lett. **28**(21), 2463–2466 (2016) 10.1364/OE.24.030245 28059300

[110] Dong, W., Lei, L., Chen, L., Yu, Y., Zhang, X.: All-optical 2×2-bit multiplier at 40 Gb/s based on canonical logic units-based programmable logic array (CLUs-PLA). J. Lightwave Technol. **38**(20), 5586–5594 (2020)

[111] Dong, W., Gu, W., Gao, X., Yu, Y., Dong, J., Lei, L., Zhang, X.: Simultaneous full set of three-input canonical logic units in a single nonlinear device for an all-optical programmable logic array. Opt. Expr. **30**(23), 41922–41932 (2022) 10.1364/OE.472746 36366656

[112] Hou, J., Chen, L., Dong, W., Zhang, X.: 40 Gb/s reconfigurable optical logic gates based on FWM in silicon waveguide. Opt. Expr. **24**(3), 2701–2711 (2016) 10.1364/OE.24.002701 26906841

[113] Dong, W., Huang, Z., Hou, J., Santos, R., Zhang, X.: Integrated all-optical programmable logic array based on semiconductor optical amplifiers. Opt. Lett. **43**(9), 2150–2153 (2018) 29714776 10.1364/OL.43.002150

[114] Cheng, Z., Dong, J., Zhang, X.: Ultracompact optical switch using a single semisymmetric Fano nanobeam cavity. Opt. Lett. **45**(8), 2363–2366 (2020) 32287233 10.1364/OL.383250

[115] Soref, R., De Leonardis, F., Passaro, V.M.: Compact resonant 2× 2 crossbar switch using three coupled waveguides with a central nanobeam. Opt. Expr. **29**(6), 8751–8762 (2021) 10.1364/OE.419126 33820316

[116] Shamir, J.: Parallel optical logic operations on reversible networks. Opt. Commun. **291**, 133–137 (2013)

[117] Miller, D.A.: Analyzing and generating multimode optical fields using self-configuring networks. Optica **7**(7), 794–801 (2020)

[118] Zhou, H., Zhao, Y., Wang, X., Gao, D., Dong, J., Zhang, X.: Self-configuring and reconfigurable silicon photonic signal processor. ACS Photonics **7**(3), 792–799 (2020)

[119] Shen, Y., Harris, N.C., Skirlo, S., Prabhu, M., Baehr-Jones, T., Hochberg, M., Sun, X., Zhao, S., Larochelle, H., Englund, D., Soljačić, M.: Deep learning with coherent nanophotonic circuits. Nat. Photonics **11**(7), 441–446 (2017)

[120] Dong, W., Hou, J., Zhang, X.: Investigation on expanding the computing capacity of optical programmable logic array based on canonical logic units. J. Lightwave Technol. **36**(18), 3949–3958 (2018)

[121] Lee, B.G., Biberman, A., Turner-Foster, A.C., Foster, M.A., Lipson, M., Gaeta, A.L., Bergman, K.: Demonstration of broadband wavelength conversion at 40 Gb/s in silicon waveguides. IEEE Photonics Technol. Lett. **21**(3), 182–184 (2009)

[122] Gao, X., Gu, W., Dong, W., Zhou, H., Lei, L., Chen, L., Yu, Y., Dong, J., Zhang, X.: Seven-channel all-optical reconfigurable canonical logic units multicasting at 40 Gb/s based on a nonlinearity-enhanced silicon waveguide. Opt. Expr. **30**(18), 32650–32659 (2022) 10.1364/OE.463665 36242321

[123] Moroney, N., Del Bino, L., Woodley, M.T., Ghalanos, G.N., Silver, J.M., Svela, A.Ø., Zhang, S., Del’Haye, P.: Logic gates based on interaction of counterpropagating light in microresonators. J. Lightwave Technol. **38**(6), 1414–1419 (2020)

[124] Essiambre, R.J., Kramer, G., Winzer, P.J., Foschini, G.J., Goebel, B.: Capacity limits of optical fiber networks. J. Lightwave Technol. **28**(4), 662–701 (2010)

[125] Liu, X., Chandrasekhar, S., Winzer, P.J.: Digital signal processing techniques enabling multi-Tb\/s superchannel transmission: an overview of recent advances in DSP-enabled superchannels. IEEE Signal Process. Mag. **31**(2), 16–24 (2014)

[126] Temprana, E., Myslivets, E., Kuo, B.P., Liu, L., Ataie, V., Alic, N., Radic, S.: Overcoming Kerr-induced capacity limit in optical fiber transmission. Science **348**(6242), 1445–1448 (2015) 26113716 10.1126/science.aab1781

[127] Ip, E.M., Kahn, J.M.: Fiber impairment compensation using coherent detection and digital signal processing. J. Lightwave Technol. **28**(4), 502–519 (2010)

[128] Tong, Z., Lundström, C., Andrekson, P.A., McKinstrie, C.J., Karlsson, M., Blessing, D.J., Tipsuwannakul, E., Puttnam, B.J., Toda, H., Grüner-Nielsen, L.: Towards ultrasensitive optical links enabled by low-noise phase-sensitive amplifiers. Nat. Photonics **5**(7), 430–436 (2011)

[129] Olsson, S.L., Corcoran, B., Lundström, C., Eriksson, T.A., Karlsson, M., Andrekson, P.A.: Phase-sensitive amplified transmission links for improved sensitivity and nonlinearity tolerance. J. Lightwave Technol. **33**(3), 710–721 (2015)

[130] Olsson, S.L., Karlsson, M., Andrekson, P.A.: Nonlinear phase noise mitigation in phase-sensitive amplified transmission systems. Opt. Expr. **23**(9), 11724–11740 (2015) 10.1364/OE.23.011724 25969263

[131] Umeki, T., Asobe, M., Takenouchi, H.: In-line phase sensitive amplifier based on PPLN waveguides. Opt. Expr. **21**(10), 12077–12084 (2013) 10.1364/OE.21.012077 23736428

[132] Umeki, T., Tadanaga, O., Asobe, M., Miyamoto, Y., Takenouchi, H.: First demonstration of high-order QAM signal amplification in PPLN-based phase sensitive amplifier. Opt. Expr. **22**(3), 2473–2482 (2014) 10.1364/OE.22.002473 24663539

[133] Lundström, C., Corcoran, B., Karlsson, M., Andrekson, P.A.: Phase and amplitude characteristics of a phase-sensitive amplifier operating in gain saturation. Opt. Expr. **20**(19), 21400–21412 (2012) 10.1364/OE.20.021400 23037263

[134] Andrekson, P.A., Karlsson, M.: Fiber-based phase-sensitive optical amplifiers and their applications. Adv. Opt. Photonics **12**(2), 367–428 (2020)

[135] Porzi, C., Bogoni, A., Contestabile, G.: Regeneration of DPSK signals in a saturated SOA. IEEE Photonics Technol. Lett. **24**(18), 1597–1599 (2012)

[136] Liebig, E., Sackey, I., Richter, T., Gajda, A., Peczek, A., Zimmermann, L., Petermann, K., Schubert, C.: Performance evaluation of a silicon waveguide for phase regeneration of a QPSK signal. J. Lightwave Technol. **35**(6), 1149–1156 (2017)

[137] Olsson, S.L., Eliasson, H., Astra, E., Karlsson, M., Andrekson, P.A.: Long-haul optical transmission link using low-noise phase-sensitive amplifiers. Nat. Commun. **9**(1), 2513 (2018) 29955056 10.1038/s41467-018-04956-5 PMC6023869

[138] Ettabib, M.A., Bottrill, K., Parmigiani, F., Kapsalis, A., Bogris, A., Brun, M., Labeye, P., Nicoletti, S., Hammani, K., Syvridis, D., Richardson, D.J., Petropoulos, P.: All-optical phase regeneration with record PSA extinction ratio in a low-birefringence silicon germanium waveguide. J. Lightwave Technol. **34**(17), 3993–3998 (2016)

[139] Karlsson, M.: Transmission systems with low noise phase-sensitive parametric amplifiers. J. Lightwave Technol. **34**(5), 1411–1423 (2016)

[140] Boggio, J.C., Marconi, J.D., Fragnito, H.L.: Experimental and numerical investigation of the SBS-threshold increase in an optical fiber by applying strain distributions. J. Lightwave Technol. **23**(11), 3808–3814 (2005)

[141] Anderson, B., Robin, C., Flores, A., Dajani, I.: Experimental study of SBS suppression via white noise phase modulation. In: Fiber Lasers XI: technology, systems, and applications. 8961, 362–368 SPIE. (2014)

[142] Zhao, P., Ye, Z., Karlsson, M., Torres-Company, V., Andrekson, P.A.: Low-noise phase-sensitive parametric amplifiers based on integrated silicon-nitride-waveguides for optical signal processing. J. Lightwave Technol. **40**(6), 1847–1854 (2022)

[143] Vazimali, M.G., Fathpour, S.: Applications of thin-film lithium niobate in nonlinear integrated photonics. Adv. Photonics **4**(3), 034001 (2022)

[144] Bottrill, K.R.H., Kakarla, R., Parmigiani, F., Venkitesh, D., Petropoulos, P.: Phase regeneration of QPSK signal in SOA using single-stage, wavelength converting PSA. IEEE Photonics Technol. Lett. **28**(2), 205–208 (2016)

[145] Croussore, K.A., Li, G.: Phase-regenerative wavelength conversion for BPSK and DPSK signals. IEEE Photonics Technol. Lett. **21**(2), 70–72 (2009)

[146] Guan, P., Da Ros, F., Lillieholm, M., Kjøller, N.K., Hu, H., Røge, K.M., Galili, M., Morioka, T., Oxenløwe, L.K.: Scalable WDM phase regeneration in a single phase-sensitive amplifier through optical time lenses. Nat. Commun. **9**(1), 1049 (2018) 29535308 10.1038/s41467-018-03458-8 PMC5849695

[147] Long, Y., Wang, A., Zhou, L., Wang, J.: All-optical wavelength conversion and signal regeneration of PAM-4 signal using a silicon waveguide. Opt. Expr. **24**(7), 7158–7167 (2016) 10.1364/OE.24.007158 27137008

[148] Salem, R., Foster, M.A., Turner, A.C., Geraghty, D.F., Lipson, M., Gaeta, A.L.: Signal regeneration using low-power four-wave mixing on silicon chip. Nat. Photonics **2**(1), 35–38 (2008)

[149] Morichetti, F., Canciamilla, A., Ferrari, C., Samarelli, A., Sorel, M., Melloni, A.: Travelling-wave resonant four-wave mixing breaks the limits of cavity-enhanced all-optical wavelength conversion. Nat. Commun. **2**(1), 296 (2011) 21540838 10.1038/ncomms1294 PMC3112537

[150] Zeng, X., Gentry, C.M., Popović, M.A.: Four-wave mixing in silicon coupled-cavity resonators with port-selective, orthogonal supermode excitation. Opt. Lett. **40**(9), 2120–2123 (2015) 25927800 10.1364/OL.40.002120

[151] Kim, C., Lu, X., Kong, D., Chen, N., Chen, Y., Oxenløwe, L.K., Yvind, K., Zhang, X., Yang, L., Pu, M., Xu, J.: Parity-time symmetry enabled ultra-efficient nonlinear optical signal processing. eLight **4**, 6 (2024) 38585278 10.1186/s43593-024-00062-w PMC10995095

[152] Xia, Y., Yang, S., Niu, J., Fu, X., Yang, L.: Strict non-blocking four-port optical router for mesh photonic network-on-chip. J. Semicond. **43**(9), 092301 (2022)

[153] Tao, Z., Tao, Y., Jin, M., Qin, J., Chen, R., Shen, B., Wu, Y., Shu, H., Yu, S., Wang, X.: Highly reconfigurable silicon integrated microwave photonic filter towards next-generation wireless communication. Photon. Res. **11**(5), 682–694 (2023)

[154] Spyropoulou, M., Pleros, N., Vyrsokinos, K., Apostolopoulos, D., Bougioukos, M., Petrantonakis, D., Miliou, A., Avramopoulos, H.: 40 Gb/s NRZ wavelength conversion using a differentially-biased SOA-MZI: theory and experiment. J. Lightwave Technol. **29**(10), 1489–1499 (2011)

[155] Zhuang, L., Roeloffzen, C.G., Hoekman, M., Boller, K.J., Lowery, A.J.: Programmable photonic signal processor chip for radiofrequency applications. Optica **2**(10), 854–859 (2015)

[156] Pérez, D., Gasulla, I., Crudgington, L., Thomson, D.J., Khokhar, A.Z., Li, K., Cao, W., Mashanovich, G.Z., Capmany, J.: Multipurpose silicon photonics signal processor core. Nat. Commun. **8**(1), 636 (2017) 28935924 10.1038/s41467-017-00714-1 PMC5608755

[157] Liu, W., Li, M., Guzzon, R.S., Norberg, E.J., Parker, J.S., Lu, M., Coldren, L.A., Yao, J.: A fully reconfigurable photonic integrated signal processor. Nat. Photonics **10**(3), 190–195 (2016)

[158] Zhang, W., Yao, J.: A fully reconfigurable waveguide Bragg grating for programmable photonic signal processing. Nat. Commun. **9**(1), 1396 (2018) 29643383 10.1038/s41467-018-03738-3 PMC5895633

[159] Zhang, W., Yao, J.: Photonic integrated field-programmable disk array signal processor. Nat. Commun. **11**(1), 406 (2020) 31964890 10.1038/s41467-019-14249-0 PMC6972927

[160] Tan, M., Xu, J., Liu, S., Feng, J., Zhang, H., Yao, C., Chen, S., Guo, H., Han, G., Wen, Z., Chen, B., He, Y., Zheng, X., Ming, D., Tu, Y., Fu, Q., Qi, N., Li, D., Geng, L., Wen, S., Yang, F., He, H., Liu, F., Xue, H., Wang, Y., Qiu, C., Mi, G., Li, Y., Chang, T., Lai, M., Zhang, L., Hao, Q., Qin, M.: Co-packaged optics (CPO): status, challenges, and solutions. Front Optoelectron. **16**(1), 1 (2023) 36939942 10.1007/s12200-022-00055-y PMC10027985

[161] Li, T., Hou, J., Yan, J., Liu, R., Yang, H., Sun, Z.: Chiplet heterogeneous integration technology—status and challenges. Electronics (Basel) **9**(4), 670 (2020)

[162] Ohira, K., Kobayashi, K., Iizuka, N., Yoshida, H., Ezaki, M., Uemura, H., Kojima, A., Nakamura, K., Furuyama, H., Shibata, H.: On-chip optical interconnection by using integrated III–V laser diode and photodetector with silicon waveguide. Opt. Expr. **18**(15), 15440–15447 (2010) 10.1364/OE.18.015440 20720923

[163] Hatori, N., Shimizu, T., Okano, M., Ishizaka, M., Yamamoto, T., Urino, Y., Mori, M., Nakamura, T., Arakawa, Y.: A hybrid integrated light source on a silicon platform using a trident spot-size converter. J. Lightwave Technol. **32**(7), 1329–1336 (2014)

[164] De Valicourt, G., Chang, C.M., Lee, J., Eggleston, M.S., Zhu, C., Sinsky, J.H., Kim, K., Dong, P., Maho, A., Brenot, R., Chen, Y.K.: Integrated hybrid wavelength-tunable III–V/silicon transmitter based on a ring-assisted Mach-Zehnder interferometer modulator. J. Lightwave Technol. **36**(2), 204–209 (2018)

[165] Puckett, M.W., Krueger, N.A.: Broadband, ultrahigh efficiency fiber-to-chip coupling via multilayer nanophotonics. Appl. Opt. **60**(15), 4340–4344 (2021) 34143123 10.1364/AO.417177

[166] Padmaraju, K., Bergman, K.: Resolving the thermal challenges for silicon microring resonator devices. Nanophotonics **3**(4–5), 269–281 (2014)

[167] Chen, S., Shi, Y., He, S., Dai, D.: Low-loss and broadband 2× 2 silicon thermo-optic Mach-Zehnder switch with bent directional couplers. Opt. Lett. **41**(4), 836–839 (2016) 26872201 10.1364/OL.41.000836

[168] Van Campenhout, J., Green, W.M., Vlasov, Y.A.: Design of a digital, ultra-broadband electro-optic switch for reconfigurable optical networks-on-chip. Opt. Express **17**(26), 23793–23808 (2009) 20052090 10.1364/OE.17.023793

[169] Birks, T.A., Russell, P.S.J., Culverhouse, D.O.: The acousto-optic effect in single-mode fiber tapers and couplers. J. Lightwave Technol. **14**(11), 2519–2529 (1996)

[170] Freiser, M.: A survey of magnetooptic effects. IEEE Trans. Magn. **4**(2), 152–161 (1968)

[171] Cocorullo, G., Rendina, I.: Thermo-optical modulation at 1.5 μm in silicon etalon. Electron. Lett. **28**(1), 83–85 (1992)

[172] Holland, M.G.: Analysis of lattice thermal conductivity. Phys. Rev. **132**(6), 2461–2471 (1963)

[173] Gao, F., Wang, Y., Cao, G., Jia, X., Zhang, F.: Improvement of sidewall surface roughness in silicon-on-insulator rib waveguides. Appl. Phys. B **81**(5), 691–694 (2005)

[174] Bellegarde, C., Pargon, E., Sciancalepore, C., Petit-Etienne, C., Hugues, V., Robin-Brosse, D., Hartmann, J.M., Lyan, P.: Improvement of sidewall roughness of submicron SOI waveguides by hydrogen plasma and annealing. IEEE Photonics Technol. Lett. **30**(7), 591–594 (2018)

[175] Sparacin, D.K., Spector, S.J., Kimerling, L.C.: Silicon waveguide sidewall smoothing by wet chemical oxidation. J. Lightwave Technol. **23**(8), 2455–2461 (2005)

[176] Wang, X., Zhou, L., Li, R., Xie, J., Lu, L., Wu, K., Chen, J.: Continuously tunable ultra-thin silicon waveguide optical delay line. Optica **4**(5), 507–515 (2017)

[177] Wen, H.S., Cui, J.B., Zhou, H., Chen, Y.F., Jin, Y., Xu, B.R., Zhai, K.P., Sun, J.Z., Guo, Y.Y., Wu, Y.R., Chen, W., Chen, W., Wang, X., Zhu, N.H., Lu, G.W., Ji, G.J., Zhou, D.C., Cheng, Y.K., Yang, D., Li, M.: 100 Gb/s NRZ OOK signal regeneration using four-wave mixing in a silicon waveguide with reverse-biased pin junction. Opt. Expr. **30**(21), 38077–38094 (2022) 10.1364/OE.471162 36258380

[178] Zhao, W., Peng, Y., Cao, X., Zhao, S., Liu, R., Wei, Y., Liu, D., Yi, X., Han, S., Wan, Y., Li, K., Wu, G., Wang, J., Shi, Y., Dai, D.: 96-Channel on-chip reconfigurable optical add-drop multiplexer for multidimensional multiplexing systems. Nanophotonics **11**(18), 4299–4313 (2022) 39634537 10.1515/nanoph-2022-0319 PMC11501234

[179] Li, M., Yin, P., Liu, Z., Dong, F., Sui, L., Ma, W., Wang, T.: Enhanced four-wave mixing in borophene-microfiber waveguides at telecom C-band. Appl. Opt. **61**(5), 1261–1267 (2022) 35201179 10.1364/AO.447664

[180] Long, Y., Gui, C., Wang, A., Hu, X., Zhu, L., Zhou, L., Wang, J.: All-optical three-input simultaneous multicasted quaternary addition/subtraction using non-degenerate FWM in a silicon waveguide and 20 Gibt/s QPSK signal. In: Optical fiber communication conference, Th2A–6. Optica Publishing Group (2016)

[181] He, A., Guo, X., Wang, T., Su, Y.: Ultracompact fiber-to-chip metamaterial edge coupler. ACS Photonics **8**(11), 3226–3233 (2021)

[182] Zhai, K., Jin, Y., Chen, Y., Chen, S., Wang, X., Wen, H., Zhu, N.: Thermal analysis and hybrid packaging design of SOI based all optical signal processing chip. In: 2021 Asia Communications and Photonics Conference (ACP), 1–3. IEEE (2021)

